# Supplementary material for: Rehabilitation cooperation and person-centred dialogue meeting for patients sick-listed for common mental disorders: 12 months follow-up of sick leave days, symptoms of depression, anxiety, stress and work ability – a pragmatic cluster randomised controlled trial from the CO-WORK-CARE project
Source: BMJ Open. 2023 Jun 9;13(6):e074137. doi: 10.1136/bmjopen-2023-074137 (PMC10277141; doi:10.1136/bmjopen-2023-074137)

BMJ Publishing Group Limited (BMJ) disclaims all liability and responsibility arising from any reliance placed on this supplemental material which has been supplied by the author(s)

Supplemental Table S1. Pragmatic characteristics of trial elements in the Co-Work-Care trial

(from https://rethinkingclinicaltrials.org/chapters/pragmatic-clinical-trial/pragmatic-elements-an-introduction-to-precis-2/)

**Trial element**	**Pragmatic characteristics in Co-Work-Care trial**
Research question	Whether enhanced and early cooperation plus early person centred dialogue is more effective in reducing total duration of sick-leave for primary care patients with reduced function because of CMD
Setting	Embedded in the routine primary care setting
Participants	Primary care patients with reduced function because of CMD
Intervention and comparator	Compares two **real-world treatments** using flexible protocols – care manager contact plus enhanced and early cooperation and early person centred dialogue vs care manager contact as usual
Outcomes	Endpoints **clinically relevant** to participants, funders, communities, and healthcare providers
Clinical importance	Purposely designed for making healthcare decisions in settings in which the intervention will be implemented and be used. Important clinical questions can be answered directly from trial results

Björkelund C, *et al. BMJ Open* 2023; 13:e074137. doi: 10.1136/bmjopen-2023-074137

Supplemental Figure S1

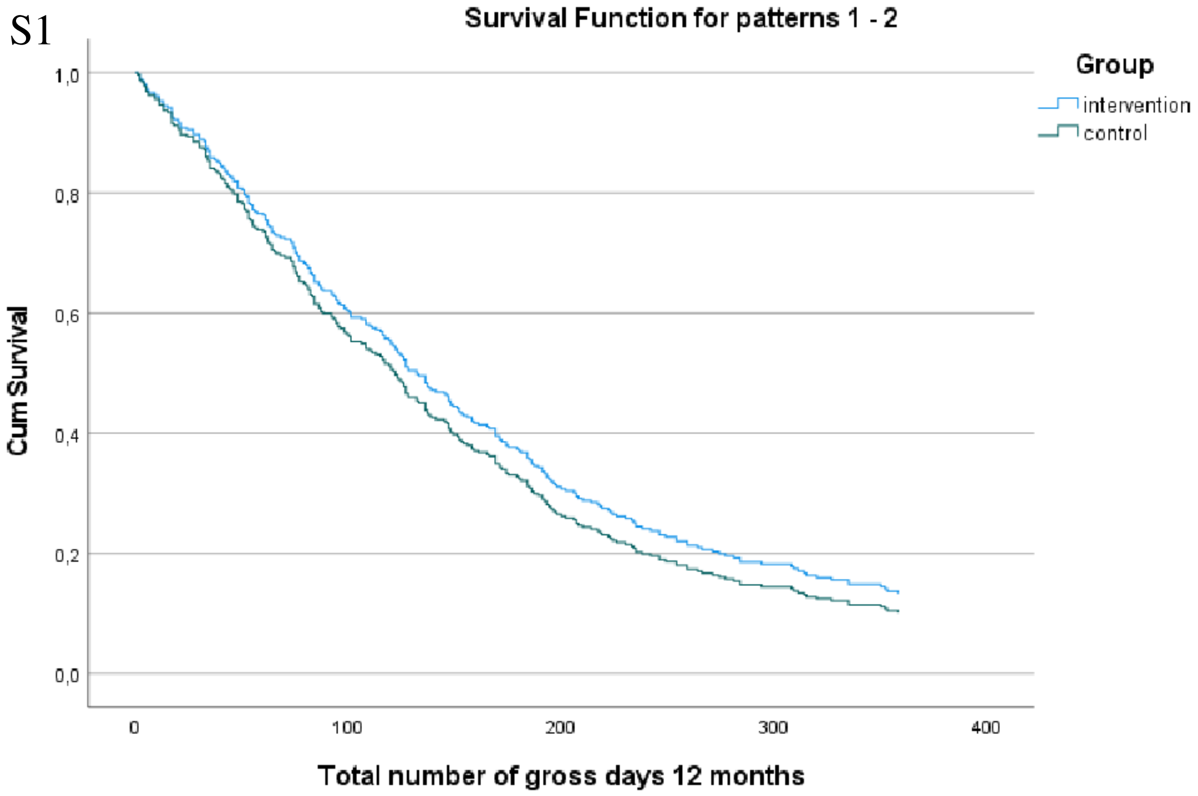

Supplemental Figure S1. Survival pattern of cumulative return to work (full or part time sick-leave to no sick-leave), gross number of days on sick-leave 12 months. Cox regression analysis controlled for age, gender and education. No significant difference between intervention (blue) and control (green) group RTW (hazard ratio=0.881, 95% CI 0.688 to 1.128).

Supplemental Figure S2 (below). Course of MADRS-S (a), GAD-7(b), KEDS (c), WAI (d), and EQ-5D (e) from baseline to 12 months follow up (adjusted means). Mixed model analysis, adjusted for clustering, age, sex, education, antidepressants at inclusion.

S2 a)

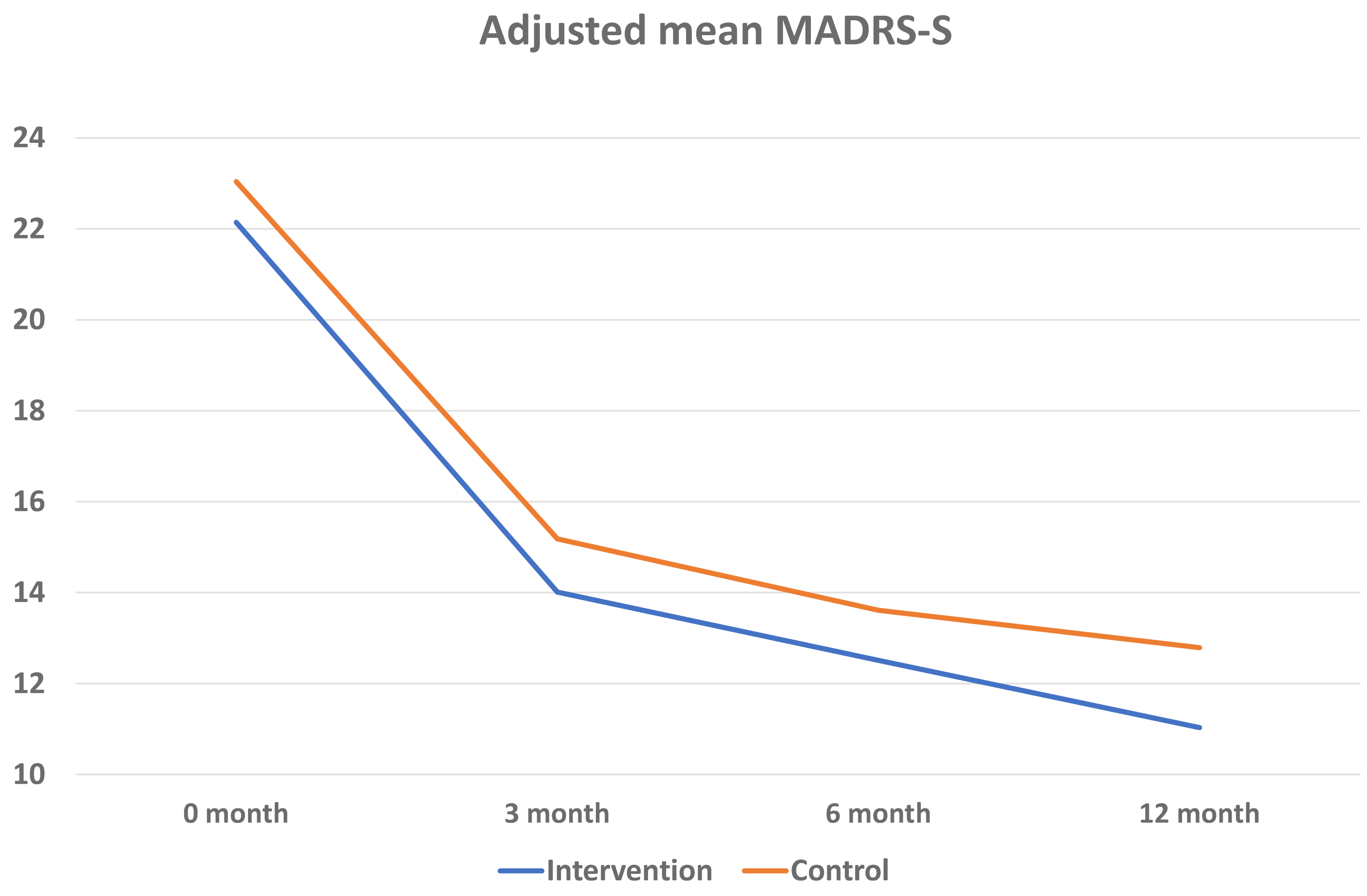
Adjusted mean MADRS-S
24
22
20
18
16
14
12
10
0 month
3 month
6 month
12 month
Intervention
Control

S2 b)

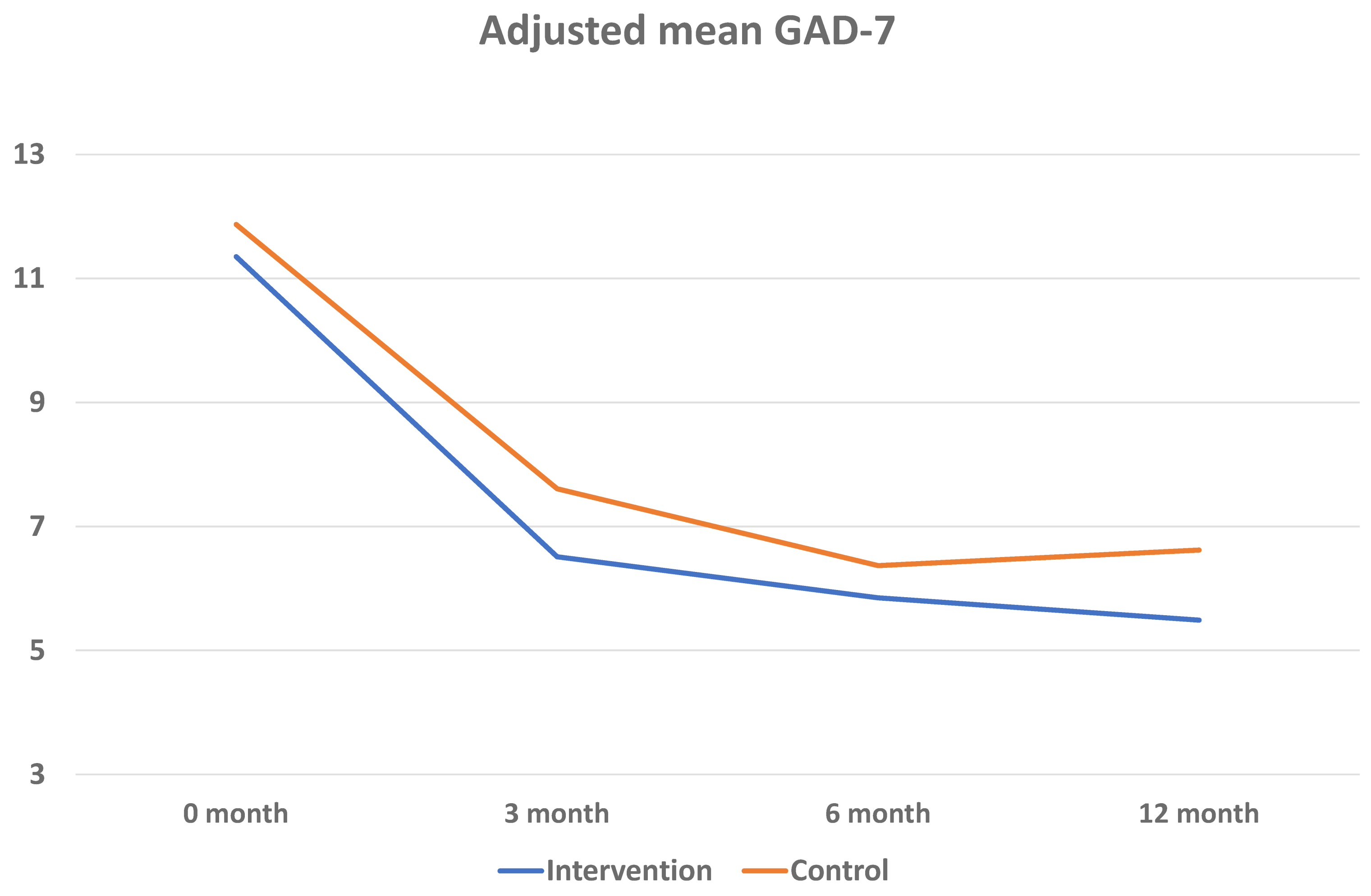
Adjusted mean GAD-7
13
11
9
7
5
3
0 month
3 month
6 month
12 month
Intervention
Control

S2 c)

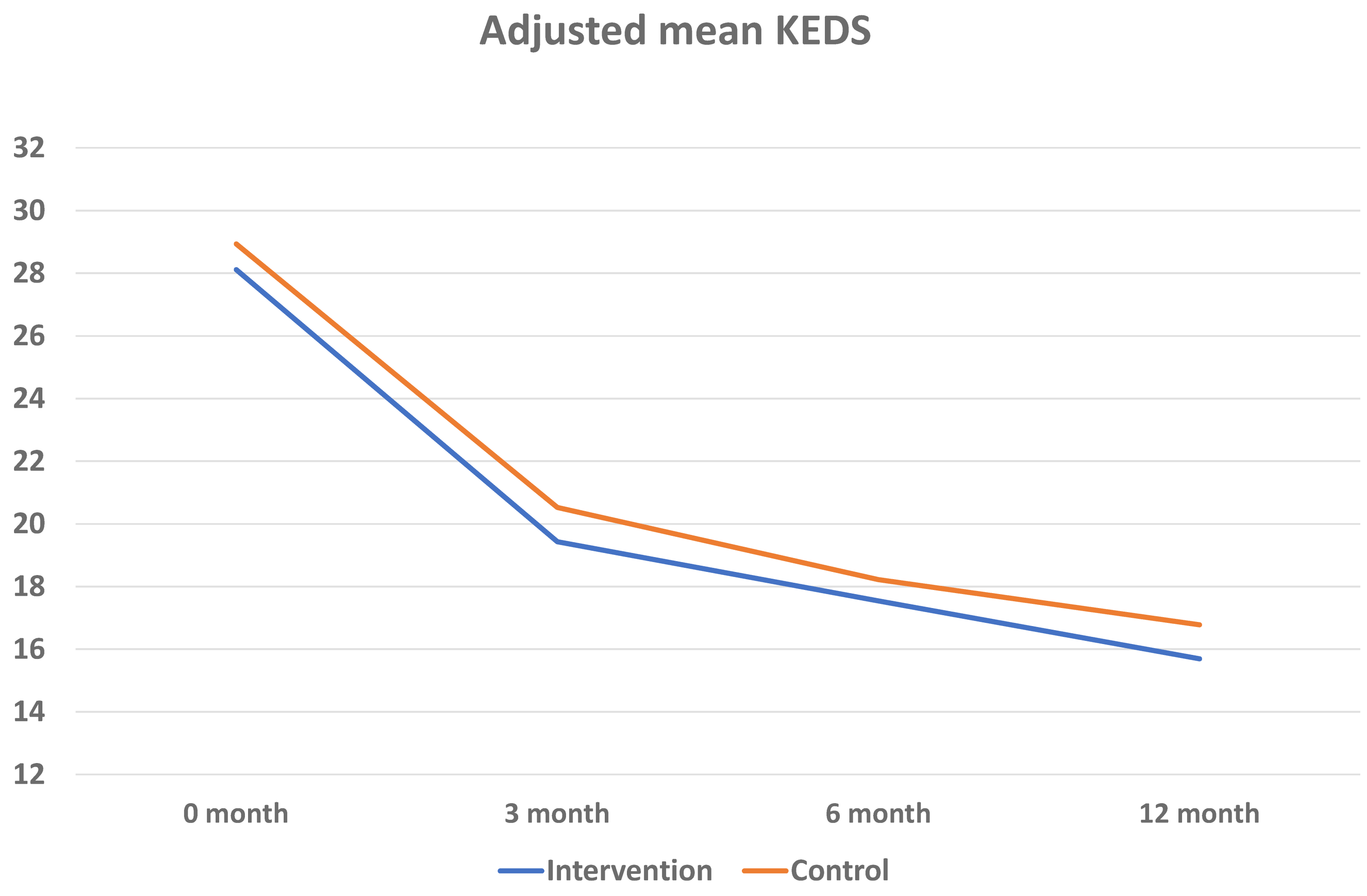
Adjusted mean KEDS
32
30
28
26
24
22
20
18
16
14
12
0 month
3 month
6 month
12 month
Intervention
Control

S2 d)

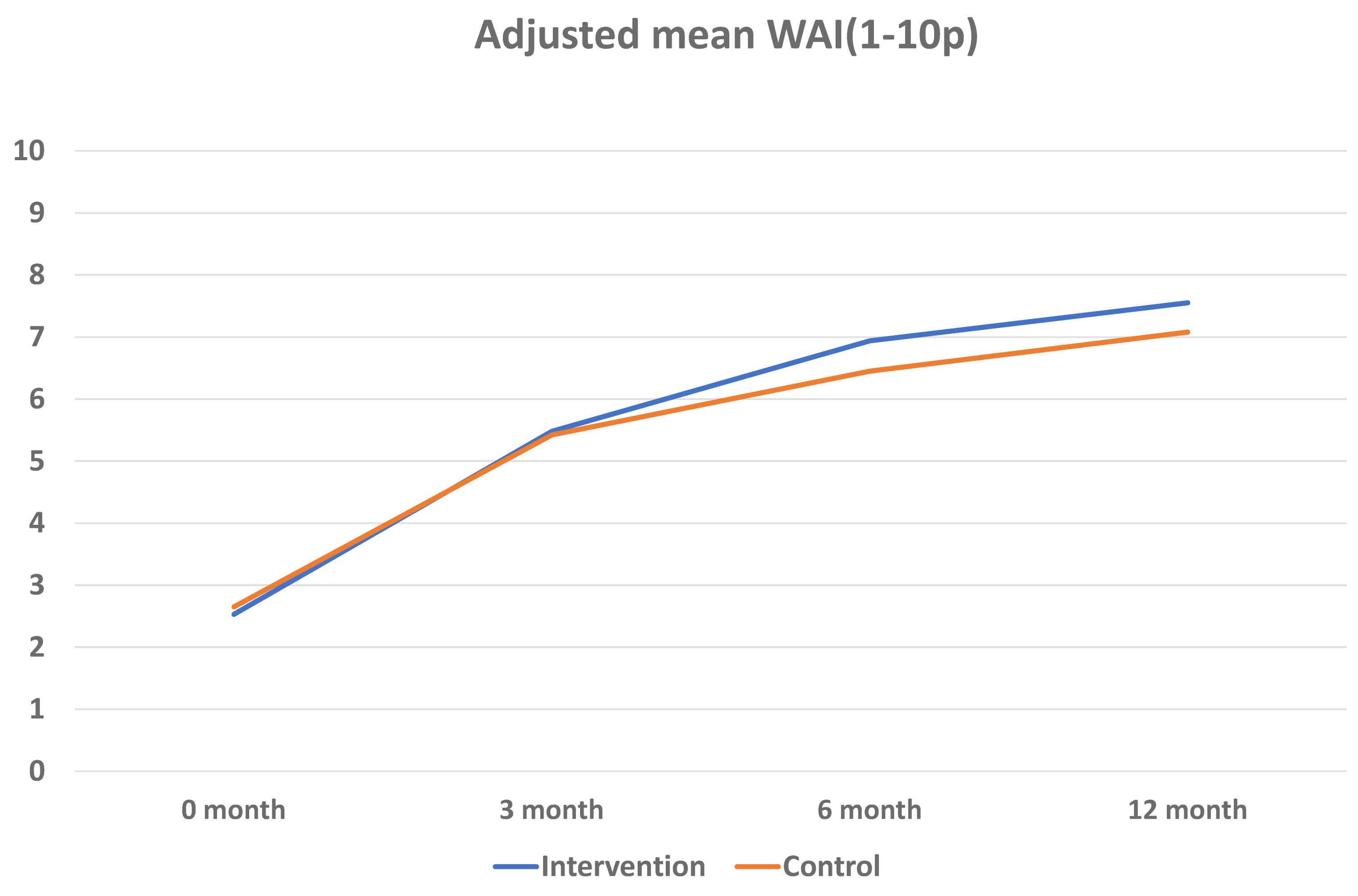
Adjusted mean WAI(1-10p)
10
9
8
7
6
5
4
3
2
1
0
0 month
3 month
6 month
12 month
Intervention
Control

S2 e)

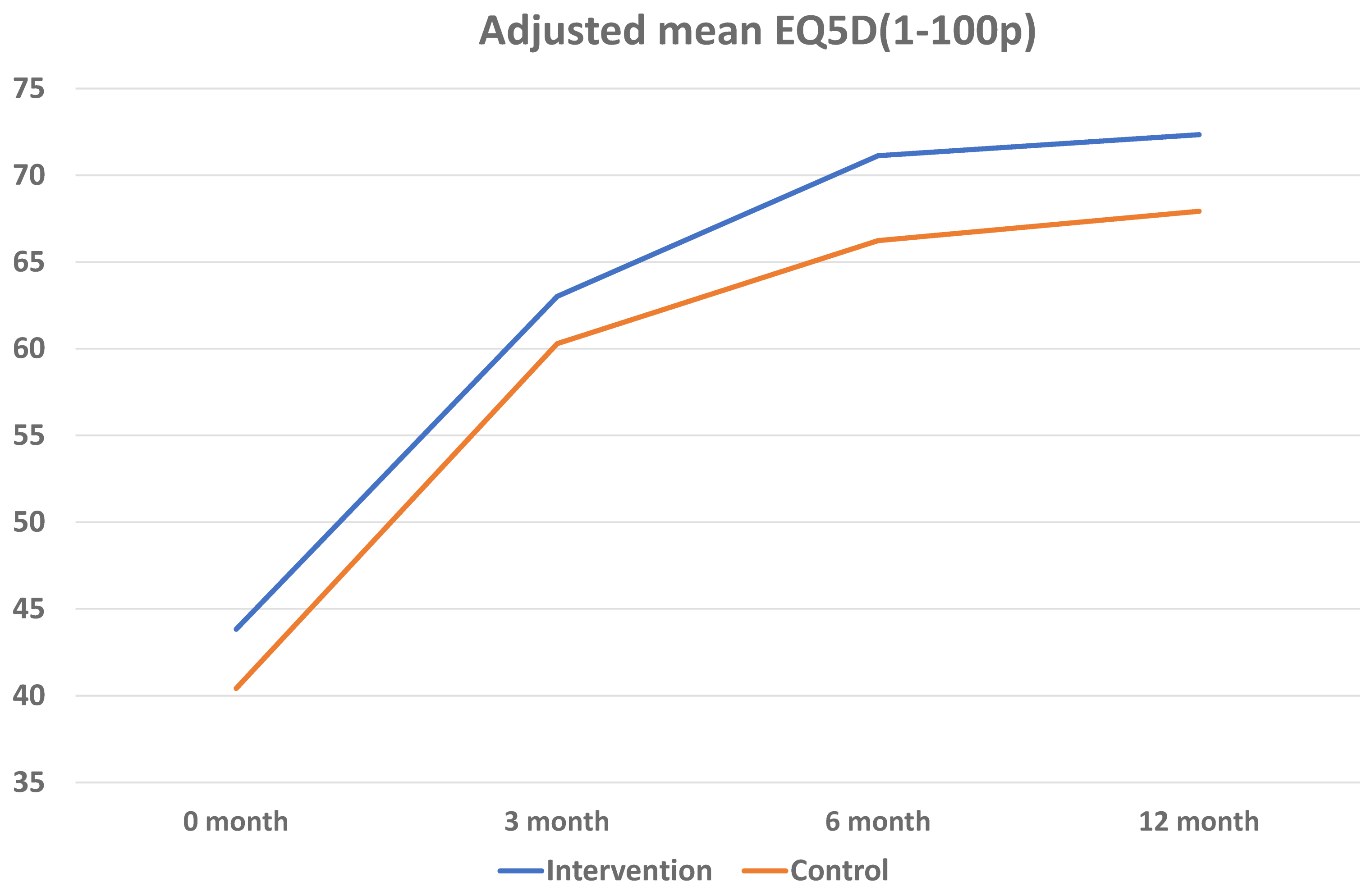
Adjusted mean EQ5D(1-100p)
75
70
65
60
55
50
45
40
35
0 month
3 month
6 month
12 month
Intervention
Control

Supplemental Figure S3 (below). Course of MADRS-S, GAD-7, KEDS for patients with moderate/high levels (n= 284) from baseline to 12 months follow up (adjusted means). Mixed model analysis, adjusted for clustering, age, sex, education, and antidepressants at inclusion.

1) All patients with MADRS-S $\geq$ 20: significant difference at 12 months;

2) All patients with GAD$\geq$10: significant difference at 12 months;

3) All patients with KEDS$\geq$ 30: no significant differences.

S3 1)

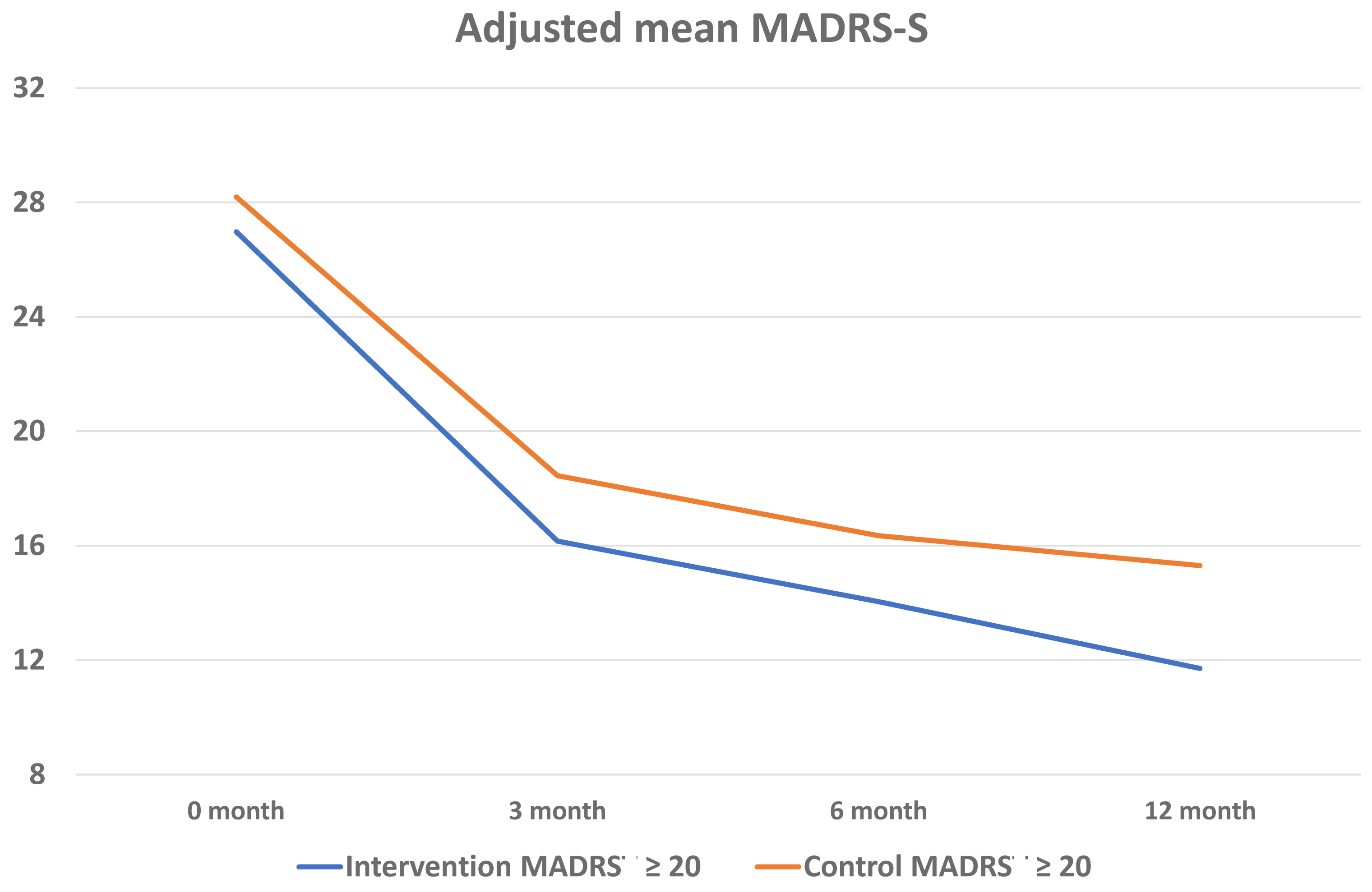
Adjusted mean MADRS-S
32
28
24
20
16
12
8
0 month
3 month
6 month
12 month
Intervention MADRS ≥ 20
Control MADRS ≥ 20

S3 2)

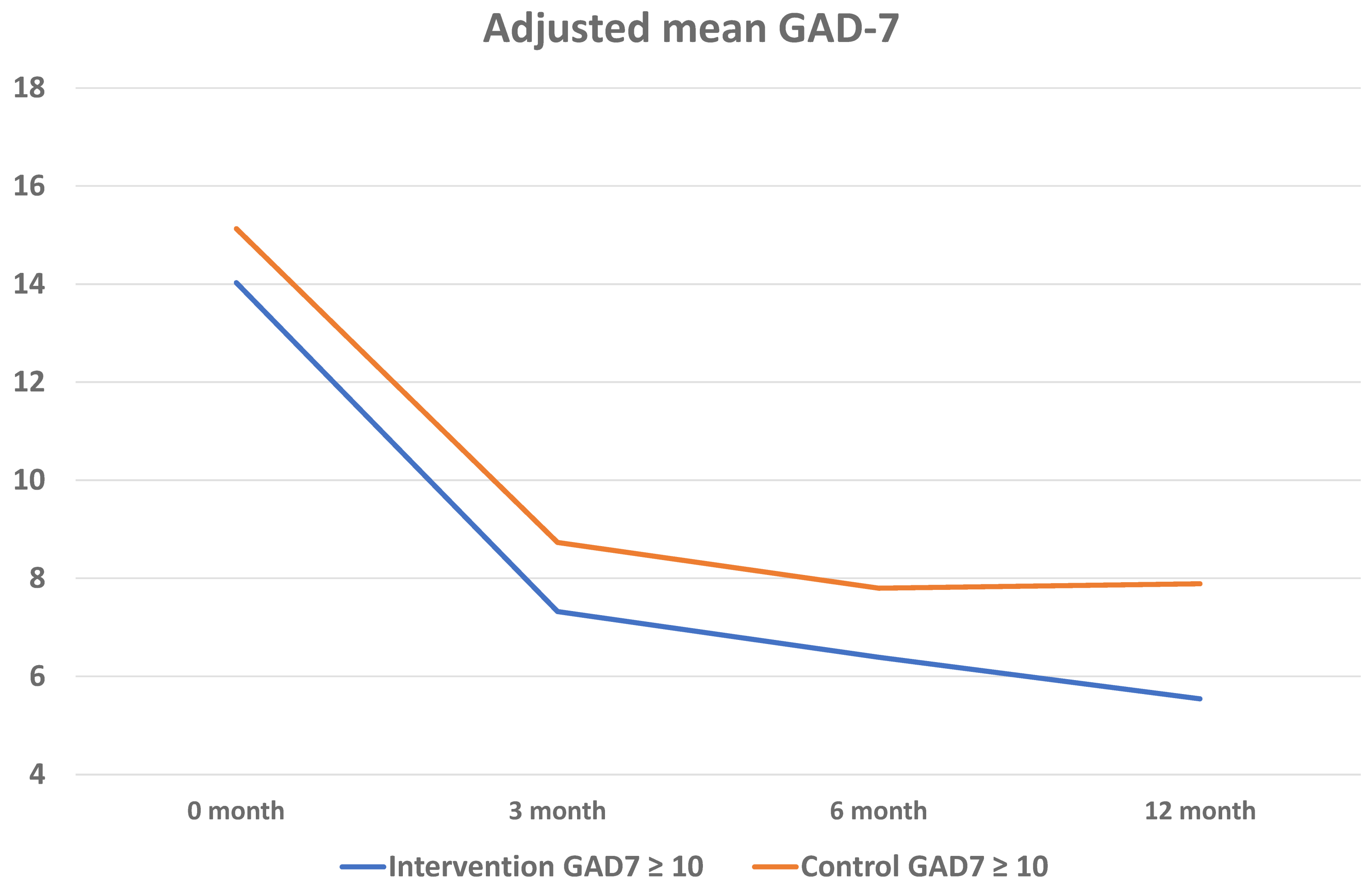
Adjusted mean GAD-7
18
16
14
12
10
8
6
4
0 month
3 month
6 month
12 month
Intervention GAD7 ≥ 10
Control GAD7 ≥ 10

BMJ Publishing Group Limited (BMJ) disclaims all liability and responsibility arising from any reliance placed on this supplemental material which has been supplied by the author(s)

S3 3)

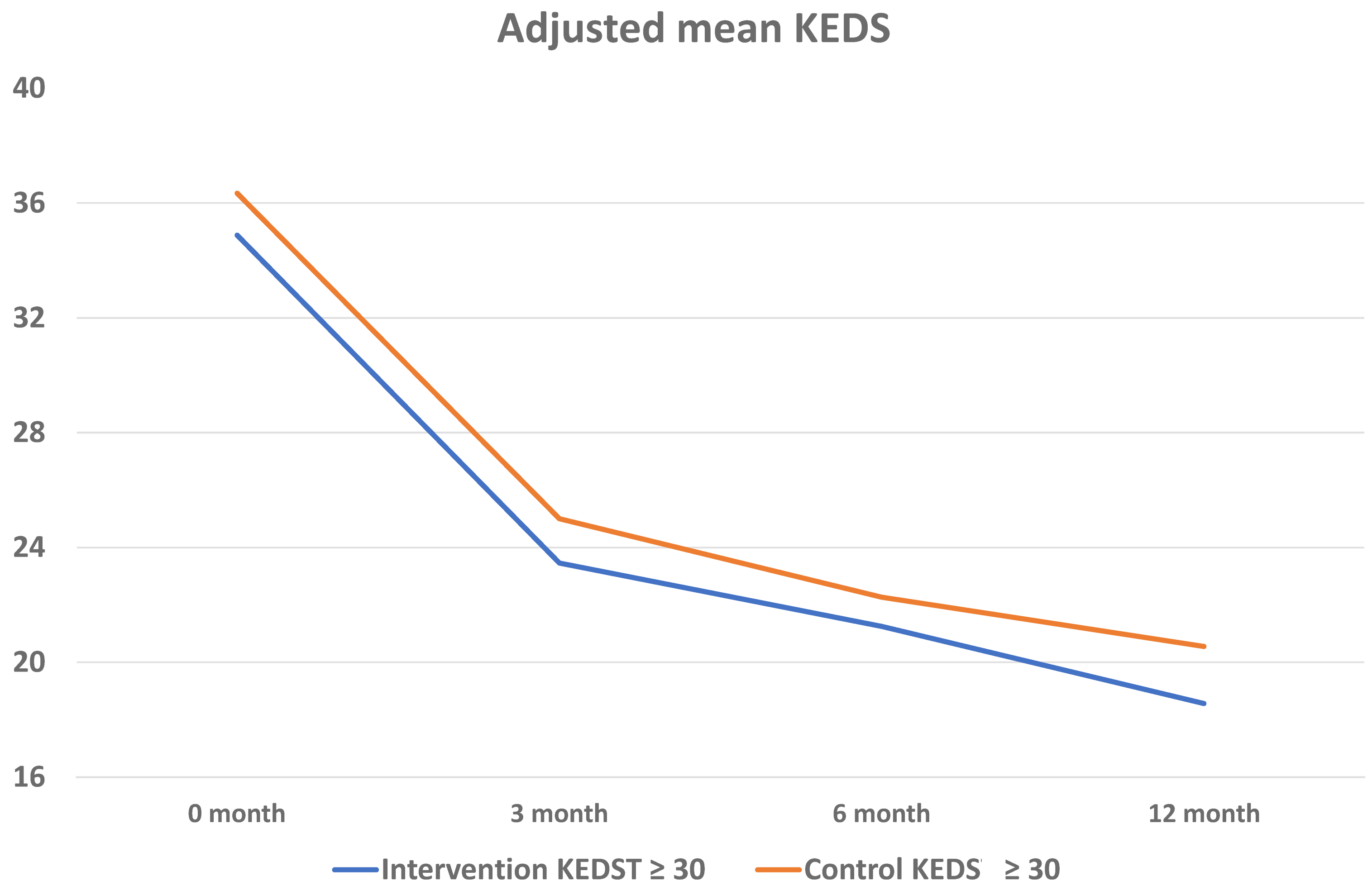

Supplement: Supplementary data [file bmjopen-2023-074137supp001.pdf]
