# Supplementary material for: Rehabilitation cooperation and person-centred dialogue meeting for patients sick-listed for common mental disorders: 12 months follow-up of sick leave days, symptoms of depression, anxiety, stress and work ability – a pragmatic cluster randomised controlled trial from the CO-WORK-CARE project
Source: BMJ Open. 2023 Jun 9;13(6):e074137. doi: 10.1136/bmjopen-2023-074137 (PMC10277141; doi:10.1136/bmjopen-2023-074137)
Supplement: Author's manuscript [file bmjopen-2023-074137.draft_revisions.pdf]

BMJ Open

BMJ Open is committed to open peer review. As part of this commitment we make the peer review history of every article we publish publicly available.

When an article is published we post the peer reviewers' comments and the authors' responses online. We also post the versions of the paper that were used during peer review. These are the versions that the peer review comments apply to.

The versions of the paper that follow are the versions that were submitted during the peer review process. They are not the versions of record or the final published versions. They should not be cited or distributed as the published version of this manuscript.

BMJ Open is an open access journal and the full, final, typeset and author-corrected version of record of the manuscript is available on our site with no access controls, subscription charges or pay-per-view fees (http://bmjopen.bmj.com).

If you have any questions on BMJ Open's open peer review process please email info.bmjopen@bmj.com

BMJ Open

Rehabilitation cooperation and person-centred dialogue meeting for patients sick-listed for common mental disorders - 12 months follow-up of sick-leave days, symptoms of depression, anxiety, stress and work ability. A pragmatic cluster-randomised controlled trial from the CO-WORK-CARE project

Journal:	*BMJ Open*
Manuscript ID	bmjopen-2023-074137
Article Type:	Original research
Date Submitted by the Author:	29-Mar-2023

Complete List of Authors:	Björkelund, Cecilia; University of Gothenburg Sahlgrenska Academy, Primary Health Care/School of Public Health and Community Medicine<br>Saxvik, Ausra; University of Gothenburg Institute of Medicine, Primary Health Care/ School of Public Health and Community Medicine<br>Svenningsson, Irene; University of Gothenburg Institute of Medicine, Primary Health Care/School of Public Health and Community Medicine<br>Petersson, Eva-Lisa; University of Gothenburg Institute of Medicine, School of Public Health and Community Medicine<br>Wiegner, Lilian; University of Gothenburg Institute of Medicine, School of Public Health and Community Medicine<br>Larsson, Maria; Institute of Neuroscience and Physiology, Sahlgrenska Academy, University of Gothenburg, Section for rehabilitation and health<br>Törnbom, Karin; University of Gothenburg, Primary Care/School of Public Health and Community Medicine; University of Gothenburg, Department of Social Work<br>Wikberg, Carl; University of Gothenburg, Primary Health Care Department of Public Health and Community Medicine<br>Ariai, Nashmil; University of Gothenburg, Primary Health Care Department of Public Health and Community Medicine<br>Nejati, Shabnam; University of Gothenburg, Primay Health Care/School of Public Health and Community Medicine<br>Hensing, Gunnel; University of Gothenburg Institute of Medicine, School of Public Health and Community Medicine<br>Hange, Dominique; University of Gothenburg, Primary Health Care/School of Public Health and Community Medicine

Keywords:	Primary Care < Primary Health Care, Depression & mood disorders < PSYCHIATRY, Anxiety disorders < PSYCHIATRY

SCHOLARONE™
Manuscripts

I, the Submitting Author has the right to grant and does grant on behalf of all authors of the Work (as defined in the below author licence), an exclusive licence and/or a non-exclusive licence for contributions from authors who are: i) UK Crown employees; ii) where BMJ has agreed a CC-BY licence shall apply, and/or iii) in accordance with the terms applicable for US Federal Government officers or employees acting as part of their official duties; on a worldwide, perpetual, irrevocable, royalty-free basis to BMJ Publishing Group Ltd ("BMJ") its licensees and where the relevant Journal is co-owned by BMJ to the co-owners of the Journal, to publish the Work in this journal and any other BMJ products and to exploit all rights, as set out in our licence.

The Submitting Author accepts and understands that any supply made under these terms is made by BMJ to the Submitting Author unless you are acting as an employee on behalf of your employer or a postgraduate student of an affiliated institution which is paying any applicable article publishing charge ("APC") for Open Access articles. Where the Submitting Author wishes to make the Work available on an Open Access basis (and intends to pay the relevant APC), the terms of reuse of such Open Access shall be governed by a Creative Commons licence – details of these licences and which Creative Commons licence will apply to this Work are set out in our licence referred to above.

Other than as permitted in any relevant BMJ Author's Self Archiving Policies, I confirm this Work has not been accepted for publication elsewhere, is not being considered for publication elsewhere and does not duplicate material already published. I confirm all authors consent to publication of this Work and authorise the granting of this licence.

For peer review only

Rehabilitation cooperation and person-centred dialogue meeting for patients sick-listed for common mental disorders - 12 months follow-up of sick-leave days, symptoms of depression, anxiety, stress and work ability. A pragmatic cluster-randomised controlled trial from the CO-WORK-CARE project

Cecilia Björkelund [1,2], Ausra Saxvik [1], Irene Svenningsson [1,2], Eva-Lisa Petersson [1,2], Lilian Wiegner [1,3], Maria EH Larsson [2,4], Karin Törnbom [1,5], Carl Wikberg [1,2], Nashmil Ariai [1,2], Shabnam Nejati [1,2], Gunnel Hensing [6], Dominique Hange [1,2]

[1] Primary Health Care/Department of Public Health and Community Medicine, Institute of Medicine, Sahlgrenska Academy, University of Gothenburg

[2] Research, Education, Development & Innovation, Primary Health Care, Region Västra Götaland, Sweden

[3] Institute of Stress Medicine, Region Västra Götaland, Sweden

[4] Unit of Physiotherapy/ Department of Health and Rehabilitation, Institute of Neuroscience and Physiology, Sahlgrenska Academy, University of Gothenburg, Gothenburg, Sweden

[5] Department of Social Work, University of Gothenburg, Gothenburg, Sweden

[6] Social Medicine/ Department of Public Health and Community Medicine, Institute of Medicine, Sahlgrenska Academy, University of Gothenburg

Corresponding author

Cecilia Björkelund, Primary Health Care/Department of Public Health and Community Medicine

Box 453

41530 Gothenburg, Sweden cecilia.bjorkelund@allmed.gu.se

For peer review only

For peer review only - http://bmjopen.bmj.com/site/about/guidelines.xhtml

Abstract

Objectives To study whether early and enhanced cooperation within the primary care centres combined with workplace cooperation via a person-centred employer dialogue meeting can reduce days on sick-leave compared with usual care manager contact for patients on sick-leave because of common mental disorders (CMD). Secondary aim: to study lapse of CMD symptoms, perceived work ability (WAI) and quality of life (QoL) during12 months.

Design Pragmatic cluster-randomised controlled trial, randomisation at primary care centre (PCC) level.

Setting 28 PCCs in Region Västra Götaland, Sweden, with care manager organisation.

Participants 30 PCCs were invited, 28 (93%) accepted invitation (14 intervention, 14 control) and recruited 341 patients newly sick-listed because of CMD (n=185 at intervention, n=156 at control PCCs).

Intervention. Complex intervention consisting of i) early cooperation among GP, care manager and a rehabilitation coordinator, plus ii) a person-centred dialogue meeting between patient and employer within 3 months. Control group: regular contact with care manager.

Main outcome measures 12 months net and gross number of sick-leave days at group level. Secondary outcomes: 12 months depression, anxiety, stress symptoms, perceived work ability (WAI) and Quality of Life (EQ-5D).

Results No significant differences were found between intervention and control groups concerning days of sick-leave (intervention net days of sick-leave mean 102.48 (SE 13.76) vs control 96.29 (SE 12.38) p=0.73), return to work (hazard ratio=0.881, 95% CI 0.688 to 1.128), or CMD symptoms, WAI or EQ-5D after 12 months.

Conclusions It is not possible to speed up CMD patients' return to work or to reduce sick-leave time by early and enhanced coordination among GP, care manager and a rehabilitation

For peer review only - http://bmjopen.bmj.com/site/about/guidelines.xhtml

coordinator, combined with early workplace contact over and above what "usual" care manager contact during 3 months provides.

Strengths and limitations of this study

- The Co-Work-Care trial was accomplished entirely in primary care.
- The intervention was adapted to a level possible to conduct in a Swedish primary care context with low level of resources for direct workplace intervention and cooperation.
- The intervention model was person-centred and emphasised the individual's perceptions of workplace conditions facilitating own return-to-work.
- Covid19 could have had possible impact on the last months of follow-up for 12 months sick-leave duration, however, this effect should have been comparable in the control group.
- The complexity of the intervention based on the individual patient's preferences, needs and values, a structured cooperation among GP, care manager and rehabilitation coordinator and a person-centred dialogue meeting with employer could be called into question concerning possible biasing factors.

Trial registration ClinicalTrials.gov Identifier: NCT03250026 First Posted: August 15, 2017. Start of recruitment of PCCs: September, 2017. Start of inclusion of patients: December, 2017

For peer review only - http://bmjopen.bmj.com/site/about/guidelines.xhtml

Background

The largest diagnostic group among sick-listed women and men in Sweden is common mental disorders (CMD), i.e., depression, anxiety syndromes and stress-related mental disorder (adjustment disorder, exhaustion disorder) (1). The yearly cumulative incidence is approximately 3% of the labour force (1). Likewise, in other parts of Europe CMD are common among persons who are sick-listed, and work-related stress is increasingly identified as a contributing cause (2,3,4,5). In fact, more than 50% of new sick-leave cases in Sweden have adjustment disorder or exhaustion disorder (ICD F43) as their certification diagnosis (1, 3), while depression disorder has decreased as certification diagnosis (1). Many of these individuals return to work after treatment, but for some return to work is slower. Knowledge of the most decisive factors for continuing sick-leave is to a great extent lacking for CMD patients today (6), but reasons include symptom severity and difficulties to adapt workplace or work tasks (6,7). Long sick-leave periods (>12 weeks) are common in Sweden, and most intervention studies have so far failed to show marked positive effects on return to work (RTW), except for interventions including increased contact with the workplace (7, 8). Prolonged RTW can lead to a more permanent exclusion from the labour market, and better methods are needed to prevent an excessively long sick-leave period that could possibly be counter-productive.

Since the largest proportion of individuals with mental illness seek primary care, usually with symptoms that manifest in depressive and/or anxiety symptoms, primary health care centres are a relevant arena for interventions (9). An organisation guided by collaborative care principles, in which a care manager coordinates care at the primary care centre (PCC) by maintaining a close and regular contact with patients and aligns efforts for their individual needs, has shown good results regarding mental and physical health improvements (10, 11, 12). In a recent systematic review, a care manager organisation at the PCC was one of few

For peer review only

factors found to increase RTW frequency when compared to already existing forms of organisation and actions (12). Other outcomes related to team-based interventions at the PCC were increased quality of care, reduction of symptoms and increased work ability, function and quality of life (9, 10, 11, 12, 13, 14). A care manager organisation seems to enhance care approaches that allow complexity, person-centredness and interaction (10,15). The collaborative care organisation handles person-centredness and complexity via the care manager, who also facilitates interaction with other psycho-social competencies, especially a psychologist/psychotherapist for the individual patient (in the Swedish study around 45-50% (10)), but also a counsellor, physiotherapist and other contacts at the PCC when needed. However, beneficial effects have primarily been shown for primary care patients with depressive illness. Thus far, evidence is lacking both concerning the entire CMD diagnosis spectrum and especially for those with a symptom burden and work ability reduction high enough to necessitate sick-leave.

In an earlier Swedish study, PRIM-CARE RCT (ClinicalTrials.gov Identifier: NCT02378272), a care manager intervention at the primary care centre improved return to work and reduced sick-leave for patients with depression compared to care as usual (10). Consequently, a wide implementation of a care manager organisation at interested PCCs has been going on since 2015 in the Region Västra Götaland, Sweden. The present Co-Work-Care trial was initiated to examine whether a care manager contact combined with early structured enhanced cooperation among the care manager, rehabilitation coordinator and general practitioner (GP) at the PCC plus a person-centred dialogue meeting with the patient’s employer would further increase and shorten RTW for CMD patients on sick-leave compared to those with “usual” care manager contact.

For peer review only - http://bmjopen.bmj.com/site/about/guidelines.xhtml

Objectives

The primary aim was to study whether an early structured enhanced cooperation among the care manager, rehabilitation coordinator and GP combined with workplace cooperation via a person-centered dialogue meeting with the patient's employer can reduce sick-leave time and increase RTW and work ability compared with care manager contact "as usual". A secondary aim was to study lapse of depression, anxiety and stress-related symptoms as well as perceived work ability and quality of life during 12 months. Thus, we regarded it as important also to examine the lapse of mental symptoms to study the effects of the intervention on the patient's mental state. As the intervention was based on structured enhanced cooperation between several staff members within the PCC, randomisation at PCC level was chosen.

Methods

Study setting and context

As described above, an implementation of the care manager function at interested PCCs has been ongoing since 2015 in the Region Västra Götaland, Sweden (inhabitants ~ 1.7 million; ~ 200 PCCs). The first part of the implementation was conducted as a randomised controlled trial (RCT) (ClinicalTrials.gov Identifier: NCT02378272) (10). Since the RCT showed positive effects concerning depression course, quality of life (QoL) and RTW (10, 14, 15), a care manager (mostly a registered nurse) for patients with CMD including stress-related disorder (adjustment disorder + exhaustion disorder) was implemented at most PCCs in the region (at around 160 PCCs). The implementation of a care manager collaborative care organisation for a team-based care was also recommended for national implementation in the Swedish guidelines for care of depression and anxiety syndromes (16).

For peer review only - http://bmjopen.bmj.com/site/about/guidelines.xhtml

In Sweden, primary care is organised in primary care centres (PCCs), which (besides several GPs) also have specially educated nurses, primary care psychologists, counsellors and physiotherapists, often working in team around the patient. The PCCs are publicly or privately driven, serving around 2,000-30,000 listed patients (around 1 GP and 2 nurses per 2,000 listed). All PCCs have, for example, a specially educated nurse who works part-time with diabetes patient care together with the GP, preferably via individually adjusted health promotion activities, while also guaranteeing accessibility and continuity for the diabetic patient. In a similar manner, the care manager's assignment is to work part-time with CMD patients to guarantee accessibility and continuity for the CMD patient and promote psycho-pedagogical support to the patient during the illness. A care manager academic education, financed via the Region Västra Götaland county council, is mandatory (Care Manager for CMD, 7.5 higher education credits).

During the same time as the care manager organisation was implemented, an implementation of a function to coordinate the sick-leave and rehabilitation process, i.e. a rehabilitation coordinator, was also carried out at all PCCs in the region. A rehabilitation coordinator was initiated in 2016 via a national project with special governmental funding to the regions to offer coordinating work to patients on sick-leave to promote return to work. A rehabilitation coordinator has an education in social insurance laws and regulations and vocational rehabilitation and serves as a regulation knowledge broker for the PCCs and a support for the patient's RTW process and workplace cooperation. The education standards vary between regions and organisations. In primary care, the rehabilitation coordinator often serves several PCCs in contrast to care managers who are part of the PCCs' ordinary personnel and working part of their duty as care managers. At some PCCs enrolled in this trial (11/28=39 %; 5/14 intervention PCCs, 6/14 control PCCs) the care manager also served as rehabilitation coordinator.

For peer review only

For peer review only - http://bmjopen.bmj.com/site/about/guidelines.xhtml

Trial design

The design was a pragmatic cluster-randomised controlled trial with complex intervention and thus randomisation was performed at PCC level (supplemental Table S1). The study adheres to Consort guidelines with extension to cluster randomized trials and the Swedish Ethics Review Board approved the study.

Participants

Invitation to PCCs to participate in a randomised controlled trial started in September 2017, and 30 PCCs in the Västra Götaland Region with an established organisation consisting of a care manager and affiliated rehabilitation coordinator were invited via the regional primary care R&D support organisation to take part in the CO-WORK-CARE study. Of the invited 30 PCCs, 28 (93%) PCCs accepted the invitation. All PCCs were informed about the trial both via information visits to every PCC before study start and by written confirmation which was obtained from every PCC, both intervention and control PCCs. All PCCs participated in educational activities concerning study protocol (figure 1, upper part). Thus, at all PCCs in the RCT, intervention as well as control, a care manager was working part-time at the PCC and a rehabilitation coordinator was affiliated.

Randomisation and masking

Randomisation was conducted at PCC level. A total of 28 primary care centres were included and divided into two strata, rural (n=14) and urban (n=14). The centres in each stratum were then divided into seven blocks of two centres each. One centre in each block was randomised to the intervention group (n=14) and one to the control group (n=14) by the Primary Health Care R&D Region Västra Götaland, Sweden (figure 1). Cluster randomisation was chosen to avoid the treatment contamination between the intervention and control groups that would

For peer review only

For peer review only - http://bmjopen.bmj.com/site/about/guidelines.xhtml

occur if patients at centres were individually randomised. No masking was possible, either concerning personnel at PCCs or research personnel.

Patients

All newly attending patients at intervention and control PCCs who were judged in need of new sick-leave due to mild/moderate depression (ICD F32, F33), anxiety syndrome (ICD F40, F41, F48) and/or stress-related mental disorder (ICD F43) (excluding post-traumatic stress disorder) were invited by the GP or the care manager to participate in the trial. Diagnosis was established by the GP, who made out the sickness certificate, and the diagnosis was confirmed by use of PRIME-MD, a diagnostic tool for depression disorder especially for primary care (17), and by results from the KEDS assessment instrument (not diagnostic) (18,19).

Exclusion criteria

Patients diagnosed with serious depression, bipolar disorder, psychosis, addiction, pregnancy > 1st trimester, cognitive impairment, post-traumatic stress syndrome or not speaking/understanding Swedish were excluded.

Intervention

The CO-WORK-CARE complex intervention consisted of i) early structured cooperation among care manager, rehabilitation coordinator and GP, and ii) a person-centred dialogue meeting between patient and employer (immediate supervisor) with the rehabilitation coordinator as facilitator.

The care for the patient was based on the patient's ongoing sick-leave, the sick-leave diagnosis, the individual care plan (which the care manager and the patient initially drew up together) and the course of the CMD symptoms. In both intervention and control arms the individual care plan was drawn up between care manager and patient, but at intervention PCCs the rehabilitation coordinator was included from start, and the care manager, the

For peer review only - http://bmjopen.bmj.com/site/about/guidelines.xhtml

rehabilitation coordinator and the GP continuously had contact about the lapse of the patient's illness and progress, see table 1. The intervention also consisted of a person-centred dialogue meeting between the employer and employee with the rehabilitation coordinator as the guide and facilitator, effectuated within 3 months from study start, as early as possible based on the patient's condition and opinion.

Table 1. Care Process in the Co-Work-Care trial. Grey parts common for control and intervention PCCs, green parts solely for intervention PCCs.

Actor	GP	Care Manager	Rehabilitation coordinator	Dialogue meeting
**Action**	**Diagnoses CMD**	**Continuous contact from start sick-leave certification**	**Initiates return to work**	**Within 12 weeks**
**First contact**	Sick-leave certification >14 days with CMD diagnosis.<br>Somatic examination<br><br>Information to care manager (and other staff when assessed needed)	Care plan together with patient based on interview and anamnesis.<br><br>Assessment with<br>MADRS-S<br>GAD7<br>KEDS<br><br>Information to other staff when therapy needed	Interview with patient when care manager indicates patient ready to start rehabilitation process.<br><br>Time for dialogue meeting agreed upon with patient.<br>Dialogue meeting decided.	Person-centred dialogue meeting between patient and employer;<br>Rehabilitation coordinator as the dialogue moderator.<br>Patient explains her/his view on return to work process
**Follow up**	Evaluates need of further sick-leave in regular reappointments	Telephone follow-up week 1,2,4,8,12<br><br>When patient ready: work questions discussed	Telephone contact with employer for interview about patient's work situation and information about dialogue meeting scope	
**Ongoing**	*Structured cooperation between GP, care manager and rehabilitation coordinator*			

For peer review only - http://bmjopen.bmj.com/site/about/guidelines.xhtml

The purpose of the person-centred dialogue meeting was above all to make it possible for the patient to inform the employer about the patient's own conception of factors that could facilitate a return to work. The dialogue meeting was a simplified dialogue meeting applied to primary care context, adapted from a more extensive protocol by Karlson and Östberg (20). The dialogue meeting was person-centred and focused on the needs of the individual patient and the patient's preferences, needs and values in the return-to-work (RTW) process (21). A person-centred dialogue meeting between the employer and the patient is not utilised in the Swedish workplace rehabilitation. Regulations state that a meeting among the workplace representative, the Social Insurance Agency, the PCC representative (most often GP, sometimes also rehabilitation coordinator) and the patient should be initiated after around 3 months of (full-time) sickness absence. This regulated meeting is not a person-centred meeting where the patient has the opportunity to describe her/his experience of the illness and thoughts about how to return to work at the workplace. In the CO-WORK-CARE study's person-centred dialogue meeting (where only three participants are present), the rehab coordinator was the mentor and guarantee for keeping the patient in the centre.

In all patient care, there was also a cooperation with the general practitioner (GP) and with the PCC's psycho-social team, but the structured and early care manager/rehabilitation coordinator /GP cooperation as well as the person-centred dialogue meeting with the employer was the added value in the intervention.

Procedures

Intervention PCCs

For the intervention PCCs an extended instruction about the complex intervention with early cooperation and person-centred dialogue meeting was conducted (8 hour session for the entire medical care personnel group including rehabilitation coordinator). During the RCT, each intervention PCC was regularly supported by a research team specialised

For peer review only - http://bmjopen.bmj.com/site/about/guidelines.xhtml

nurse/counsellor to secure adherence to the complex intervention. The complex intervention with early cooperation and person-centred dialogue meeting was added to the care manager care-as-usual function provided at the control PCCs, which already included engagement of psychologist/psychotherapist as well as other members of the psychosocial team according to national guidelines and focus on the patient's symptom development and preferences. Early cooperation was carried out by i) information from the care manager to the rehabilitation coordinator about the patient's status and care planning concerning workplace contact in conjunction with the care manager's and patient's initial meeting, and ii) in addition to care-as-usual team meetings with the psycho-social team when psychologist/other staff were engaged, regular meetings among the care manager, GP and rehabilitation coordinator when the care manager indicated that the patient was ready to start rehabilitation process (table 1).

Control PCCs

At the control PCCs, care as usual (CAU) consisted of: structured care manager function at the PCC with care manager contact with the patient during 3 months, as described in the PRIM-CARE trial (10). The care manager function included an initial 1 hour meeting between patient and care manager, where a care plan was established adapted to the patient's condition and preferences, and an agreement about the communication between patient and care manager during the next 12 weeks, mainly contacts by telephone, was drawn up (Table 1). The care manager contact was a way of increasing accessibility and continuity for the patient and was provided in addition to other therapeutic or pharmacologic treatment according to the patient's needs (10). This involved the engagement of a psychologist/ psychotherapist in around every second patient, as well as other members of the psychosocial team when needed (10). Contacts with GP and other competencies were provided according to the needs of the individual patient. Contact with rehabilitation coordinator was often only made in case of long sick-list duration (usually >2 months).

For peer review only - http://bmjopen.bmj.com/site/about/guidelines.xhtml

Control PCCs were also supported concerning patient recruitment and follow-up.

Patient and public involvement

Patients and/or the public were not involved in the design, conduct, or reporting, or dissemination plans of this research.

Outcomes

Primary outcomes: Number of sick-leave days (collected via the Micro-Data for Analysis of the Social Insurance System (MiDAS database)) after 12 months at group level (intervention vs control patients) for comparison. Proportion of patients who returned to full time work, time until full RTW, number/proportion of patients on sick-leave at 3, 6 and 12 months.

Secondary outcomes: Patient-related outcome measures with self-assessment instruments MADRS-S (22), GAD-7 (23), KEDS (18), EQ-5D (24) and WAI (25) course. Remission according to MADRS-S (≤12), GAD ≤ 4, KEDS (≤18), frequency of antidepressant therapy, frequency of psychotherapeutic contacts.

Self-assessment instruments used for PROMs

Depression was measured with the *Montgomery Asberg Depression Rating Scale-Self* (MADRS-S), which includes rating of mood, feelings of unease, sleep, appetite, ability to concentrate, initiative, emotional involvement, pessimism and zest for life (22). Every item is rated from 0 to 6 points with a total of 54 points where points 0-12 are classified as no depression, 13-19 points as mild depression, 20-34 points as moderate depression and >34 points as severe depression (22). We included individuals with ≤ 34 points.

For peer review only - http://bmjopen.bmj.com/site/about/guidelines.xhtml

Anxiety was measured by the *General Anxiety Disorders Scale-7* (GAD-7) (23). The GAD-7 consists of 7 items. Each question gives 0-3 points with a total of 21 points, classified as follows: >4 as mild GAD, >9 as moderate GAD and >14 as severe GAD (23).

Exhaustion disorder was measured by the *Karolinska Exhaustion Disorder Scale* (KEDS) (18). KEDS is a self-rating scale which consists of nine items, each ranging from 0 to 6 points with a scale range of 0-54, where points >18 indicate an increased risk for exhaustion disorders (18).

To measure health-related quality of life, the *EuroQoL-5D* (EQ-5D) five-dimensional questionnaire was used, measuring health status with one question for each of the following five dimensions: mobility, self-care, usual activities, pain/discomfort, and anxiety/depression (24).

Work ability was measured by the *Work Ability Index* (WAI) (25). WAI is a summary measure of seven items (range 7-49): current work ability, work ability in relation to the demands of the job, number of current diseases, estimated work impairment, sick-leave during the past year, own prognosis of work ability and mental resources (25).

Statistics

Standard statistical methods were used for descriptive statistics. Continuous variables were analysed by independent sample t-test or Mann-Whitney U test and categorical variables and frequencies by Pearson chi-square test. Mixed model analysis with repeated measures was used to compare means of intra-individual change in depressive, anxiety and stress symptoms and QoL scores between the intervention group and the control group. These analyses were adjusted for cluster randomisation of PCC, and pre-specified variables age, sex, education, and antidepressants at inclusion. GLM univariate analyses were performed for comparison of

For peer review only - http://bmjopen.bmj.com/site/about/guidelines.xhtml

For peer review only

total number of 12 months sick-leave days between intervention and control group and were adjusted for cluster randomisation of PCC, age, sex, education and antidepressants at inclusion. Cox regression analysis was done to study RTW between the intervention and the control group during 12 months of sick-leave with control of the variables age, sex and education.

The statistical analyses were made using statistical software SPSS, version 28 and SAS, version 9.4. Statistical significance was set at $p<0.05$. No multiple adjustments were considered.

Power Calculation

Based on a recently accomplished RCT study (10) with randomisation at the PCC level, where a significant difference in RTW could be identified in a group of 83 vs 88 patients on sick-leave at baseline in intervention and control groups, we made the assumption that 150 patients in each group would be sufficient for reaching a power of 80% to detect a difference of 3 units between the two groups with a significance level of 10%. The underlying assumption was a standard deviation in the group of 10 units, a within-subject correlation of 0.4, and a within-cluster correlation of 0.1, i.e. a design effect of 1.9 to correct for having a cluster analysis. The underlying assumption was also that there was a time limit for including patients in an RCT based both on PCCs' capacity to participate in a complex intervention and maintain adherence to the intervention, as well as a time limit considering changes in society and population, and thus inclusion should not exceed 2.5 years.

To be able to conduct analyses for the group of patients with stress-related mental disorder (adjustment disorder, exhaustion disorder), patients with F43 as main or contributing diagnosis were oversampled during 2020-02-01 to 2020-06-30.

For peer review only - http://bmjopen.bmj.com/site/about/guidelines.xhtml

Results

Inclusion of patients started December 2017 and was completed end of June 2020. In total 499 patients recently on sick-leave (<7 weeks) because of depression (F32, F33), anxiety syndrome (F40, F41, F48) and/or stress-related mental disorder (adjustment disorder, exhaustion disorder) (F43) were invited (Fig 1 flowchart). At the intervention PCCs, 308 patients were initially invited, but 40 did not meet inclusion criteria, 83 declined to participate and 185 agreed to participate (89%). At the control PCCs, 191 patients were initially invited, but 13 did not meet inclusion criteria, 22 declined to participate and 156 accepted to participate (88%). In total, 341 patients participated, 54% in intervention and 46% in control group.

Table 2 shows baseline data for intervention and control groups. There were differences concerning age (lower mean age control group) and percentage of men (higher in control group) as well as sedentary level of leisure time physical activity (higher level sedentary in control group).

At the intervention PCCs, early cooperation among care managers, rehabilitation coordinators and the patients' GPs was carried out. Collaboration with team members such as psychotherapists, medical social workers, physiotherapists and occupational therapists was carried out when this was deemed relevant/needed. Out of the185 intervention group patients, 102 (55%) had a person-centred dialogue meeting with the employer within 3 months, in most cases at the PCC. The reason for not having a dialogue meeting was mainly early return to work. There was a significant difference in mean number of days on sick-leave between patients who had and patients who did not have a dialogue meeting: mean number of days on sick-leave, 192 vs 117 (patients with vs patients with no dialogue meeting), $p<0.001$.

For peer review only - http://bmjopen.bmj.com/site/about/guidelines.xhtml

Table 2. Baseline data for intervention (n=185) and control group (n=156) patients, total=341 patients, included in the CO-WORK-CARE trial (December 2017 until June 2020).

	Total patient group		Intervention group		Control group	
	**n**	**%**	**n**	**%**	**n**	**%**
**Women**	275	80.6	157	84.9	118	75.6
**Men**	66	19.4	28	15.1	38	24.4
**Occupation Working**	328	96.8	178	96.2	150	97.4
**Studying**	1	0.3	0	0	1	0.6
**In search of work/other**	10	2.9	7	3.8	3	1.9
**Hours of work Full-time**	299	87.9	164	88.6	135	87.1
**Other (25-75%)**	41	12.1	21	11.4	20	12.9
**Marital status Cohabiting**	250	74.0	142	78	108	69.2
**Single**	88	26.0	40	22	48	30.8
**Born Outside Nordic Country**	32	9.4	14	7.6	18	11.5
**Educational level Primary**	20	5.9	13	7	7	4.5
**Secondary**	194	56.9	105	56.8	89	57.0
**University/ college**	127	37.2	67	36.2	60	38.5
**Physical activity leisure time sedentary**	55	16.3	23	12.6	32	20.8
**Smoking Yes+ sometimes**	77	22.6	36	19.5	41	26.5
**Alcohol high (>- 8 p AUDIT)**	27	9.0	16	9.8	11	8.1
**Low socioeconomic status**	178	58.6	96	57.1	82	60.3
**Antidepressant medication**	110	32.3	63	34.1	47	30.1
**Sick-leave last year, self-reported (Yes)**	132	40.9	78	43.8	54	37.2
**Sick- leave diagnosis Depression (F32, F33)**	64	18.8	39	21.1	25	16
**Anxiety syndrome (F40, F41, F48)**	48	14.1	22	11.9	26	16.7
**Stress related mental disorder (F43)**	200	58.7	111	60.0	89	57.1
	**mean**	**SD**	**mean**	**SD**	**mean**	**SD**
**Age**	41.3	11.2	44.2	10.9	37.9	10.7
**MADRS-S**	22.3	8.0	21.7	8.0	22.9	8.1
**GAD-7**	11.6	4.9	11.2	4.7	12.2	5.1
**KEDS**	28.7	8.7	28.2	8.5	29.3	9.1
**EQ5D**	42.8	17.3	43.8	17.1	41.5	17.6
**WAI**	2.56	2.4	2.43	2.4	2.71	2.4

For peer review only - http://bmjopen.bmj.com/site/about/guidelines.xhtml

Vocational outcomes - primary outcome

Analysis of gross and net number of days on sick-leave for intervention and control groups in the Co-Work-Care trial for CMD diagnoses during12 months follow-up showed that there was no significant difference concerning net number of sick-leave days between intervention and control groups (Table 3). The analyses were conducted both for sick-leave due to CMD and for sick-leave due to all diagnoses (mental as well as somatic) during 12 months. All these analyses showed non-significant results for sick-leave days but with shorter duration for control group patients. We also analysed total number of sick-leave days for patients with mild CMD only (n=57), as well as patients with moderate-high CMD symptoms only (n=284), both of which analyses did not show any significant differences concerning sick-leave days (table 3).

Table 3. Gross and net number of days on sick leave for intervention and control groups in the CO-WORK-CARE trial for CMD diagnose (depression and anxiety disorders + adjustment and exhaustion disorders), all diagnoses, and for net number of days for mild and moderate-high CMD diagnoses, respectively. Mixed model analysis adjusted for clustering and age, sex, education, and antidepressants at inclusion Comparison of total number of 12 months sick leave days between intervention and control group.

	**Intervention**		**Control**		
	Mean	SE	Mean	SE	p
GROSS Total number of days all CMD diagnoses	147.30	16.96	125.96	15.16	0.331
NET Total number of days CMD diagnoses	102.48	13.76	96.29	12.38	0.726

For peer review only - http://bmjopen.bmj.com/site/about/guidelines.xhtml

GROSS Total number of days all diagnoses	153.66	16.24	135.82	14.38	0.391
NET Total number of days all diagnoses)	108.98	13.08	104.99	11.68	0.810
NET Total number of days mild CMD diagnoses (n=57)	82.44	20.55	80.97	21.05	0.959
NET Total number of days moderate-high CMD diagnoses (n=284)	115.12	14.12	110.69	12.63	0.803

Supplemental Figure S1 shows survival plot from Cox regression analysis of cumulative return to work (full time or part time sick-leave to no sick-leave), gross number of days on sick-leave 12 months, controlled for age, gender and education. There was no significant difference between intervention and control groups, hazard ratio (HR)=0.881 with 95% CI 0.688 to 1.128.

Secondary outcomes (PROMs)

There were no significant differences in levels at 3, 6 and 12 months between the intervention and control groups concerning depression (MADRS-S), anxiety (GAD-7), or stress (KEDS) symptoms, WAI or QoL (EQ-5D) (table 4, Supplemental Figure S2). For participants with medium/high levels of depression (MADRS-S) and anxiety (GAD-7) symptoms, there were significant differences at 12 months between intervention and control groups, with lower levels in the intervention group. However, there were no differences at all concerning stress symptoms (KEDS) (table 4, Supplemental Figure S3).

For peer review only - http://bmjopen.bmj.com/site/about/guidelines.xhtml

Table 4. Start to 3, 6 and 12 months follow up of differences of mean values between intervention and control group of MADRS-S, GAD-7, KEDS, EQ-5D, and WAI in the CO-WORK-CARE trial, all patients, and patients with low and moderate high levels, respectively. Mixed model analysis adjusted for clustering, age, sex, education, and antidepressants at inclusion.

	Intervention Mean (SE)	Control Mean (SE)	Mean difference (SE)	95% CI	p
MADRS-S at start	22.14 (0.81)	23.04 (0.81)	-0.896 (1.10)	(-3.126, 1.335)	0.421
MADRS-S 3 months	14.01 (0.86)	15.18 (0.88)	-1.17 (1.18)	(-3.549, 1.199)	0.326
MADRS-S 6 months	12.51 (0.86)	13.61 (0.90)	-1.10 (1.19)	(-3.490, 1.292)	0.361
MADRS-S 12 months	11.03 (0.87)	12.79 (0.89)	-1.76 (1.19)	(-4.158, 0.631)	0.146
GAD-7 at start	11.35 (0.44)	11.87 (0.45)	-0.524 (0.597)	(-1.712, 0.664)	0.383
GAD-7 3 months	6.51 (0.47)	7.61 (0.50)	-1,10 (0.66)	(-2.405, 0.200)	0.096
GAD-7 6 months	5.85 (0.43)	6.37 (0.46)	-0.52 (0.59)	(-1.689, 0.658)	0.385
GAD-7 12 months	5.49 (0.46)	6.62 (0.49)	-1.13 (0.64)	(-2.402, 0.140)	0.081
KEDS at start	28.11 (1.09)	28.93 (1.06)	-0.823 (1.47)	(-3.814, 2.167)	0.579
KEDS 3 months	19.43 (1.15)	20.52 (1.16)	-1.082 (1.59)	(-4.275, 2.110)	0.498
KEDS 6 months	17.54 (1.17)	18.22 (1.18)	-0.679 (1.62)	(-3.926, 2.568)	0.676
KEDS 12 months	15.69 (1.19)	16.78 (1.20)	-1.095 (1.65)	(-4.399, 2.209)	0.509
EQ-5D at start	43.83 (1.89)	40.42 (1.86)	3.41 (2.54)	(-1.748, 8.569)	0.188
EQ-5D 3 months	63.02 (2.10)	60.29 (2.15)	2.73 (2.91)	(-3.086, 8.552)	0.351
EQ-5D 6 months	71.13 (2.08)	66.23 (2.15)	4.90 (2.88)	(-0.866, 10.669)	0.094

For peer review only - http://bmjopen.bmj.com/site/about/guidelines.xhtml

EQ-5D 12 months	72.34 (2.08)	67.92 (2.12)	4.43 (2.87)	(-1.309, 10.168)	0.128
WAI at start	2.53 (0.29)	2.65 (0.28)	-0.12 (0.39)	(-0.901, 0.663)	0.761
WAI 3 months	5.48 (0.32)	5.42 (0.33)	0.06 (0.44)	(-0.833, 0.944)	0.901
WAI 6 months	6.94 (0.31)	6.45 (0.31)	0.49 (0.43)	(-0.360, 1.340)	0.254
WAI 12 months	7.55 (0.31)	7.08 (0.31)	0.47 (0.43)	(-0.376, 1.323)	0.269
MADRS-S>19 at start	26.97 (0.63)	28.19 (0.62)	-1.21 (0.82)	(-2.880, 0.457)	0.150
MADRS-S>19, 3 months	16.16 (0.91)	18.44 (0.96)	-2.28 (1.28)	(-4.822, 0.255)	0.078
MADRS-S>19, 6 months	14.04 (0.96)	16.35 (1.05)	-2.31 (1.37)	(-5.016, 0.396)	0.094
MADRS-S>19, 12 months	11.71 (0.92)	15.30 (1.30)	-3.59 (1.31)	(-6.189, -0.993)	**0.007**
GAD-7>9 at start	14.03 (0.37)	15.13 (0.37)	-1.10 (0.597)	(-2.089, -0.112)	**0.030**
GAD-7>9, 3 months	7.32 (0.58)	8.73 (0.63)	-1,42 (0.83)	(-3.069, 0.230)	0.091
GAD-7>9, 6 months	6.39 (0.53)	7.80 (0.58)	-1.41 (0.76)	(-2.908, 0.094)	0.066
GAD-7>9, 12 months	5.54 (0.58)	7.89 (0.63)	-2.34 (0.83)	(-3.985, -0.696)	**0.006**
KEDS>29 at start	34.88 (0.73)	36.34 (0.70)	-1.46 (0.93)	(-3.365, 0.445)	0.128
KEDS>29, 3 months	23.46 (1.14)	25.00 (1.19)	-1.537 (1.60)	(-4.704, 1.631)	0.339
KEDS>29, 6 months	21.25 (1.23)	22.27 (1.28)	-1.02 (1.73)	(-4.436, 2.404)	0.558
KEDS>29, 12 months	18.56 (1.23)	20.55 (1.30)	-1.99 (1.73)	(-5.428, 1.447)	0.254

Bold figures, p<0.05

For peer review only - http://bmjopen.bmj.com/site/about/guidelines.xhtml

Table 5 shows participants on sick-leave at baseline, 3, 6 and 12 months. At 3 months, there was a significant difference between intervention and control groups with lower percentage of patients on sick-leave in the control group, but no significant differences at 6 and 12 months. Table 5 also shows comparisons between participants in intervention and control groups with mild and moderate-high levels, respectively, concerning depression symptoms, anxiety symptoms and stress symptoms at respective point in time.

Percentage of patients on antidepressants and patients receiving psychotherapy contact did not show any significant differences at 3, 6 or 12 months (Table 5).

Subgroup analyses

Analysis including only patients that participated in a person-centred dialogue meeting (n=102) compared to control group did not reveal different results from comparison of the total intervention and control groups.

Harmful events

No harmful events, suicide attempts or psychiatric hospital care were registered for patients included in the CO-WORK-CARE trial during the 12 months observation time.

For peer review only - http://bmjopen.bmj.com/site/about/guidelines.xhtml

Table 5. Number and percentage of participants in intervention and control groups with mild and moderate-high levels, respectively, of depression symptoms, anxiety symptoms and stress symptoms according to MADRS-S, GAD-7 and KEDS. Levels at baseline, 3, 6, and 12 months, as well as number and percentage of participants on sick-leave at respective point in time, on antidepressants and receiving psychotherapy contact. Bold figures indicate significant difference between intervention and control groups.

	Baseline		3 months		6 months		12 months	
	Intervention	Control	Intervention	Control	Intervention	Control	Intervention	Control
	n (%)	n (%)	n (%)	n (%)	n (%)	n (%)	n (%)	n (%)
On sick-leave	184 (99.5)	156 (100)	**114 (62.0)**	**80 (51.3)**	66 (35.9)	46 (29.5)	28 (15.2)	24 (15.4)
MADRS-S>12	160 (87.4)	143 (91.7)	**72 (46.5)**	**75 (58.6)**	62 (42.5)	54 (48.6)	49 (34.3)	49 (43.4)
MADRS-S>19	116 (63.4)	100 (64.1)	39 (25.2)	35 (27.3)	27 (18.5)	25 (22.5)	**15 (10.5)**	**23 (20.4)**
GAD>4	165 (92.2)	140 (92.1)	91 (59.9)	90 (70.9)	87 (59.6)’	71 (63.4)	73 (51.8)	61 (53.5)
GAD>9	120 (67.0)	99 (65.1)	33 (21.7)	40 (31.5)	**21 (14.4)**	**28 (25.0)**	**19 (13.5)**	**31 (27.2)**
KEDS>18	162 (88.5)	132 (86.5)	82 (52.2)	77 (60.2)	68 (46.6)	52 (46.0)	52 (36.4)	44 (38.9)
KEDS>29	94 (51.4)	83 (54.6)	26 (16.6)	27 (21.1)	17 (11.6)	18 (15.9)	11 (7.7)	12 (10.6)
Anti-depressant use	63 (34.1)	47 (30.1)	60 (38.2)	50 (39.1)	54 (36.5)	41 (36.0)	49 (34.0)	40 (35.1)
Psychotherapy contact	-	-	70 (44.6)	71(55.0)	58 (39.2)	39 (34.2)	28 (19.4)	15 (13.2)

For peer review only

For peer review only - http://bmjopen.bmj.com/site/about/guidelines.xhtml

Discussion

Main findings

The main finding was that there were no differences in sick-leave days between intervention and control groups. A structured early cooperation among care manager, GP and rehabilitation coordinator at the PCC combined with a person-centred dialogue meeting between the sick-listed person and employer, with the rehabilitation coordinator as a moderator, did not lead to shortened sick-leave time in comparison with usual care manager contact only. Patients with CMD diagnoses – both when sick-leave days were studied for CMD diagnoses only as well as total sick-leave for all diagnoses – showed no significant differences concerning sick-leave days.

However, for patients with moderate to high symptom levels of depression and anxiety there were also significant differences in levels of CMD depression and/or anxiety symptoms at 12 months between intervention and control groups, but no difference at all concerning stress symptoms. This indicates that a closer cooperation and a planned person-centred dialogue meeting could be beneficial for the mental well-being of persons with moderate-high symptom levels of depression/anxiety, as indicated by significantly lower levels of depression and anxiety symptoms after 12 months. This is an important finding, as individuals with severe symptoms of depression and anxiety may be the most difficult to help and have been shown to have the longest sick-leave period among patients with CMD (6, 7).

Strengths and weaknesses

The strengths of the present study are several. The trial was accomplished entirely in primary care. All participating PCC personnel as well as research personnel had very good contextual knowledge, and the intervention was adapted to a level possible to conduct in a Swedish

For peer review only - http://bmjopen.bmj.com/site/about/guidelines.xhtml

primary care context with low level of resources for direct workplace intervention and cooperation. The intervention model was person-centred and emphasised the individual's perceptions of workplace conditions facilitating own RTW. We succeeded in achieving the planned sample size of patients, despite the occurrence of the Covid19 epidemic during the last 3 months before the 12 months follow-up was finalised for the latest included participants.

Among limitations, the possible impact Covid19 could have had on the last months of follow-up for RTW and 12 months sick-leave duration should be mentioned. However, this effect should have been comparable in the control group. Another limitation was that only around 55% of patients in the intervention group took part in the person-centred dialogue meeting. However, a major reason for this was that the dialogue meeting was not deemed relevant or needed for the patients with early return to work in the fast rehabilitation process, and this was mostly due to the patient's own preference. The complexity of the intervention based on the individual patient's preferences, needs and values, a structured cooperation among GP, care manager and rehabilitation coordinator and a person-centred dialogue meeting with employer could be called into question concerning possible biasing factors. However, primary care is a complex clinical context where the handling of care problems is guided by the individual patient's needs as the starting point, and the pragmatic design of the trial compensates at least partly for multiple biasing factors.

A further limitation was that the trial was only possible to carry out in a primary health care system where a collaborative care model and team-based care were implemented. However, the collaborative care model with a care manager function has also been implemented in several European countries such as the UK, Netherlands, and Italy, in addition to Canada and the US (26). The care manager organisation implemented in the Region Västra Götaland has

For peer review only - http://bmjopen.bmj.com/site/about/guidelines.xhtml

been judged to represent a collaborative care programme for depression and anxiety disorder that makes possible personalised care planning and shared decision making (27).

Other studies

Several complex interventions, of which some mainly in the primary care context, aiming at increasing RTW and reducing sick-leave time have been performed. Bakker et al already in 2007 published a cluster-randomised controlled educational trial consisting of a minimal intervention for patients with stress-related mental disorders on sick-leave in primary care (MISS) that aimed at reducing sick-leave and preventing chronicity of symptoms (7). No superior effect of MISS was found concerning duration of sick-leave or severity of symptoms compared to care as usual (GP contact at PCCs) after 12 months (7). The latest published RCT on interventions aimed at integrating vocational rehabilitation and mental healthcare in people with depression and anxiety, where an integrated intervention both in healthcare and at the workplace was compared to improved mental care and care as usual, showed no increased RTW, although a higher proportion in work at 12 months was shown for the integrated intervention (28). The authors concluded that the goal of a very fast return to work is not necessarily beneficial, and that integration gave slightly better outcomes, but the clinical significance, i.e. health advantage, was questionable (28).

Interpretation of findings

The results of the trial indicate that providing more intense contacts with care and the workplace for patients with CMD with a symptom burden and reduction in work ability high enough to cause need of sick-leave did not lead to earlier return to full time work participation compared to the usual level of support to the patient provided by the care manager in a collaborative care organisation together with access to psychologic and other

For peer review only - http://bmjopen.bmj.com/site/about/guidelines.xhtml

therapeutic competences at the PCC. It seems that it is not possible to hurry up the processes that these patients need. Building a person-centred care process based on accessibility, continuity and close contact with a care manager makes it possible to adjust the care and timing to the individual patent's needs. The level of support and care that the care manager provides for the patient during the initial 3 months of the CMD illness seems 'good enough'. Extra care efforts on top of this, as provided in this intervention, do not seem to improve the patient's state. It is possible that a more thorough dialogue with greater competency is needed or, alternatively, repeated meetings to achieve a faster RTW. The person-centred dialogue meeting may achieve a better understanding on the part of the employer for the worker's health situation but a change in the work situation might not be possible anyway.

RCT trials have shown effectiveness of collaborative care and care management in patients with depression, and the CO-WORK-CARE trial further develops the collaborative care/care manager intervention with structured early cooperation between the PCC competencies, including the GP and rehabilitation personnel, and patients on sick-leave because of depression, anxiety and stress-related mental disorder. Care management was the basis of care in both arms, while the early and structured cooperation among the care manager, rehabilitation coordinator and the GP coupled with the person-centred dialogue meeting with employer the was the added value of the intervention. The effects of the structured cooperation were only discernible on the patient level. Early and structured cooperation coupled with a dialogue meeting can thus be said to be more effective in the long run for patients concerning their moderate-serious depression and anxiety symptoms than usual care manager contact, but not at all more effective concerning sick-leave duration. Interview studies of patients' and personnel's perceptions of working according to the CO-WORK-CARE model reveal that patients perceived the person-centred dialogue meeting with the employer as positive (29), and care managers and rehabilitation coordinators appreciated the

For peer review only - http://bmjopen.bmj.com/site/about/guidelines.xhtml

cooperation (30). The doctors in particular regarded the CO-WORK-CARE model as one that facilitated their often complex and frustrating work situation, and they perceived that they gained better control and support for the care of the patient through the close collaboration (31).

Implications

A good enough way to help the patient back to work in a salutogenic way seems primarily to be to provide accessibility, continuity, close contact and emphasis on person-centred care, to follow the patient continuously and to await start of workplace contact until the patient is ready for this.

For peer review only

Acknowledgements

We want to thank all primary care centres in Västra Götaland who have been engaged in the CO-WORK-CARE study for their engagement and participation.

Data sharing statement

Data are not publicly available due to Swedish law but are available from the authors on reasonable request. The data are stored at the Gothenburg University, Arvid Wallgrens backe 7, 40530 Göteborg, Sweden. Contact details: Cecilia Björkelund Orcid iD: 0000- 0003- 4083- 7342.

Competing interests

All authors have completed the Unified Competing Interest form (available on request from the corresponding author) and declare: no support from any organisation for the submitted work ;no financial relationships with any organisations that might have an interest in the submitted work in the

For peer review only - http://bmjopen.bmj.com/site/about/guidelines.xhtml

previous three years; no other relationships or activities that could appear to have influenced the submitted work.

Contributors

CB was the principal investigator and guarantor of the project. CB, E-LP, IS, DH and GH participated in the design of the work. CB, E-LP, IS, DH and NA handled the data and had the main responsibility for conducting the analyses, GH, SN, IJ contributed to conduction of additional analyses. CB, AS, GH, E-LP, IS, DH, IJ, NA, SN, KT, ML, CW and LW were responsible for the writing of the paper. All authors contributed to the interpretation of data and critical text revision and read and approved the final version of this manuscript. The corresponding author attests that all listed authors meet authorship criteria and that no others meeting the criteria have been omitted.

Transparency declaration

The manuscript's guarantor affirms that the manuscript is an honest, accurate, and transparent account of the study being reported; that no important aspects of the study have been omitted; and that any discrepancies from the study as planned and registered have been explained.

Copyright

The Corresponding Author has the right to grant on behalf of all authors and does grant on behalf of all authors, an exclusive licence and/or a non-exclusive licence for contributions from authors who are: i) UK Crown employees; ii) where BMJ has agreed a CC-BY licence shall apply, and/or iii) in accordance with the terms applicable for US Federal Government officers or employees acting as part of their official duties; on a worldwide, perpetual, irrevocable, royalty-free basis to BMJ Publishing Group Ltd ("BMJ") its licensees.

The Submitting Author accepts and understands that any supply made under these terms is made by BMJ to the Submitting Author unless you are acting as an employee on behalf of your employer or a postgraduate student of an affiliated institution which is paying any applicable article publishing charge ("APC") for Open Access articles. Where the Submitting Author wishes to make the Work available on an Open Access basis (and intends to pay the relevant APC), the terms of reuse of such Open Access shall be governed by a Creative Commons licence – details of these licences and which licence will apply to this Work are set out in our licence referred to above.

For peer review only

Ethical approval

This study conforms to the principles outlined in the Declaration of Helsinki. All participants were handed written and oral information about the purpose of the study, confidentiality and voluntary nature of participation as well as right to withdraw from the study at any time. Informed consent was obtained from all participants.

Written permission for conduct of study was obtained from the head of the regional primary healthcare authority and all participating primary care centres. Ethical approval was obtained from the Regional Ethical Review Board Gothenburg, Sweden Dnr: 459-17; T745-18.

Funding

This work was supported by Grants from Forte Dnr 2016-07412, Dnr 2018-01266 and grants from the Swedish state under the agreement between the Swedish government and the county councils, the ALF agreement (ALFGBG-722441, ALFGBG-965520**).** The funding organisations have no role in the planning, execution or analyses of the trial.

ORCID iDs

Cecilia Björkelund http://orcid.org/0000-0003-4083-7342

Irene Svenningsson http://orcid.org/0000-0002-7421-8171

Dominique Hange http://orcid.org/0000-0003-1114-444

For peer review only - http://bmjopen.bmj.com/site/about/guidelines.xhtml

References

1. Försäkringskassan. Sjukfrånvaro i psykiatriska diagnoser. En registerstudie av Sveriges arbetande befolkning i åldern 20–69 år. Socialförsäkringsrapport 2020:8. (Swedish Social Insurance Agency. Mental disorder sick leave – A register study of the Swedish working population in ages 20 to 69 years. Social Insurance Report 2020.8. English summary) Available at https://www.forsakringskassan.se/download/18.7fc616c01814e179a9f329/1656660446139/sjukfranvaro-i-psykiatriska-diagnoser-socialforsakringsrapport-2020-8.pdf. (Accessed 27th March 2023)
2. WHO methods and data sources for global burden of disease estimates 2000-2019. WHO Geneva December 2020. https://cdn.who.int/media/docs/default-source/gho-documents/global-health-estimates/ghe2019_daly-methods.pdf. (Accessed 27th March 2023)
3. Bakker IM, Terluin B, van Marwijk HWJ, van der Windt D. et al. A Cluster-Randomised Trial Evaluating an Intervention for Patients with Stress-Related Mental Disorders and Sick Leave in Primary Care. PLOS Clin Trial 2007;2(6):e26.
4. McCrone P, Dhanasiri S, Patel A, Knapp M, Lawton -Smith S. Paying the price. The cost of mental health in England to 2026. Kings Fund 2008. Available at : https://www.kingsfund.org.uk/sites/default/files/Paying-the-Price-the-cost-of-mental-health-care-England-2026-McCrone-Dhanasiri-Patel-Knapp-Lawton-Smith-Kings-Fund-May-2008_0.pdf. (Accessed 27th March 2023)
5. Hassard J, Teoh R H, Visockaite G, Dewe P, Cox T. The cost of work-related stress to society: A systematic review. Journal of Occupational Health Psychology. 2018; 23(1): 1–17.
6. Vingård E. Psykisk ohälsa, arbetsliv och sjukfrånvaro. (Mental Health, working life and sick leave. In Swedish). Forte. ISBN: 978-91-88561-31-2 https://forte.se/app/uploads/2015/04/psykisk-ohalsa-arbetsliv.pdf. (Accessed 27th March 2023)
7. Nystuen P, Hagen KB, Herrin J. Mental health problems as a cause of long term sick leave in the Norwegian workforce. SJPH 2001;29:175-182.
8. Salomonsson S, Santoft F, Lindsäter E, et al. Cognitive-behavioural therapy and return-to-work intervention for patients on sick leave due to common mental disorders: a randomised controlled trial. Occup Environ Med. 2017 Dec;74(12):905-912.
9. Lejtzen N, Sundquist J, Sundquist K, Li X. Depression and anxiety in Swedish primary health care: prevalence, incidence, and risk factors. Eur Arch Psychiatry Clin Neurosci. 2014;264(3):235-45
10. Björkelund C, Svenningsson I, Hange D, et al. Clinical effectiveness of care managers in collaborative care for patients with depression in Swedish primary health care: a pragmatic cluster randomized controlled trial. BMC Family Practice. 2018;19:28.
11. Huffman JC, Niazi SK, Rundell JR, Sharpe M, Katon WJ. Essential articles on collaborative care models for the treatment of psychiatric disorders in medical settings: a publication by the academy of psychosomatic medicine research and evidence-based practice committee. Psychosomatics. 2014;55(2):109-22
12. Nieuwenhuijsen K, Verbeek JH, Neumeyer-Gromen A, Verhoeven AC, Bültmann U, Faber B. Interventions to improve return to work in depressed people. Cochrane Database of Systematic Reviews 2020, Issue 10. Art. No.: CD006237.
13. Arends I, Bruinvels DJ, Redebergen DS, et al. Interventions to facilitate return to work in adults with adjustment disorders. Cochrane Database of Systematic Reviews 2012, Issue 12. Art. No CD006389. https://doi.org/10.1002/14651858.CD006389.pub2 (27th March 2023).
14. Petersson EL, Hange D, Udo C, Björkelund C, Svenningsson I. Long-term effect of a care manager on work ability for patients with depression - the PRIM-CARE RCT. Work. 2022;72(2):601-609.
15. Udo C, Svenningsson I, Björkelund C, Hange D, Jerlock M, Petersson E-L. An interview study of the care manager function – Opening the door to continuity of care for patients with depression in primary care. Nursing Open. 2019;6(3):974-82.

For peer review only - http://bmjopen.bmj.com/site/about/guidelines.xhtml

16. National Board of Health and Welfare. National guidelines for depression and anxiety syndromes. (In Swedish). https://www.socialstyrelsen.se/globalassets/sharepoint-dokument/artikelkatalog/nationella-riktlinjer/2021-4-7339.pdf (Accessed 27th March 202327th).
17. Spitzer RL, Williams JB, Kroenke K, et al. Utility of a new procedure for diagnosing mental disorders in primary care. The PRIME-MD 1000 study. JAMA 1994; 272:1749-1756.)
18. Besèr A, Sorjonen K, Wahlberg K, Peterson U, Nygren Å, Åsberg M. Construction and evaluation of a self rating scale for stress-induced Exhaustion Disorder, the Karolinska Exhaustion Disorder Scale. Scandinavian Journal of Psychology. 2013;55: 72–82.)
19. Wiegner L, Hange D, Björkelund C, Ahlborg Jr G. Prevalence of perceived stress and associations to symptoms of exhaustion, depression and anxiety in a working age population seeking primary care – an observational study. BMC Family Practice 2015 16:38 DOI 10.1186/s12875-015-0252-7.
20. Karlson B, Östberg K. ADA ArbetsplatsDialog för Arbetsåtergång. (Place of work dialogue for return to work. In Swedish) Användarmanual v.1.3. Lund: Avd för arbets- och miljömedicin. Lunds universitet; 2014.
21. Fors A, Ekman I, Taft C, et al. Person-centred care after acute coronary syndrome, from hospital to primary care - A randomised controlled trial. Int J Cardiol. 2015;187:693-9).
22. Montgomery SA, Asberg M. A new depression scale designed to be sensitive to change. The British Journal of Psychiatry. 1979;134:382-9.
23. Spitzer RL, Kroenke K, Williams JB, et al. A brief measure for assessing generalized anxiety disorder: the GAD-7. Arch Intern Med 2006;166(10):1092-7.
24. EuroQol Group, EuroQol - a new facility for the mesurement of health-related quality of life. The EuroQol Group, Health Policy, 16 (19990) 199-208.
25. Ilmarinen J. The Work Ability Index (WAI). Occup Med. 2007;57(2):160.
26. Sighinolfi C, Nespeca C, Menchetti M, Levantesi P, Belvederi Murri M, Berardi D. Collaborative care for depression in European countries: a systematic review and meta-analysis. J Psychosom Res. 2014 Oct;77(4):247-63.
27. Menear M, Girard A, Dugas M, Gervais M, Gilbert M, Gagnon M-P. Personalized care planning and shared decision making in collaborative care programs for depression and anxiety disorders: A systematic review. PLoS One. 2022 Jun 10;17(6): e0268649
28. Hoff A, Poulsen RM, Fisker J, et al. Integrating vocational rehabilitation and mental healthcare to improve the return-to-work process for people on sick leave with depression or anxiety: results from a three-arm, parallel randomised trial. Occup Environ Med. 2022 Feb;79(2):134-142.
29. Saxvik A, Törnbom K, Petersson EL, et al. Experiences of patients with common mental disorders concerning team-based primary care and a person-centered dialogue meeting: An intervention to promote return to work. PLoS One. 2022 Jul 8;17(7):e0271180.
30. Petersson EL, Törnbom K, Hange D, et al. The experiences of care managers and rehabilitation coordinators of a primary care intervention to promote return to work for patients with common mental disorders: a qualitative study. BMC Fam Pract. 2020 Dec 18;21(1):272.
31. Saxvik A, Svenningsson I, Tornbom K, et al. "Gaining control through close collaboration" - GPs' experiences of a Collaborative Care Model for patients with Common Mental Disorders who need sick leave certification. BJGP Open. 2022:BJGPO.2022.0042.

For peer review only - http://bmjopen.bmj.com/site/about/guidelines.xhtml

Figure legends

Figure1. Flow chart for the CO-WORK-CARE trial; inclusion of primary care centres (upper part) and inclusion of patients, subdivided by intervention and control identification of primary care centres (lower part).

Supplemental Figure S1. Survival pattern of cumulative return to work (full or part time sick-leave to no sick-leave), gross number of days on sick-leave 12 months. Cox regression analysis controlled for age, gender and education. No significant difference between intervention (blue) and control (green) group RTW (hazard ratio=0.881, 95% CI 0.688 to 1.128).

Supplemental Figure S2. Course of MADRS-S (a), GAD-7(b), KEDS (c), WAI (d), and EQ-5D (e) from baseline to 12 months follow up (adjusted means). Mixed model analysis, adjusted for clustering, age, sex, education, antidepressants at inclusion.

Supplemental Figure S3. Course of MADRS-S, GAD-7, KEDS for patients with moderate/ high levels (n= 284) from baseline to 12 months follow up (adjusted means). Mixed model analysis, adjusted for clustering, age, sex, education, and antidepressants at inclusion. a) All patients with MADRS-S $\geq$ 20: significant difference at 12 months; b) All patients with GAD$\geq$10: significant difference at 12 months; c) All patients with KEDS$\geq$ 30: no significant differences.

For peer review only

Fig 1

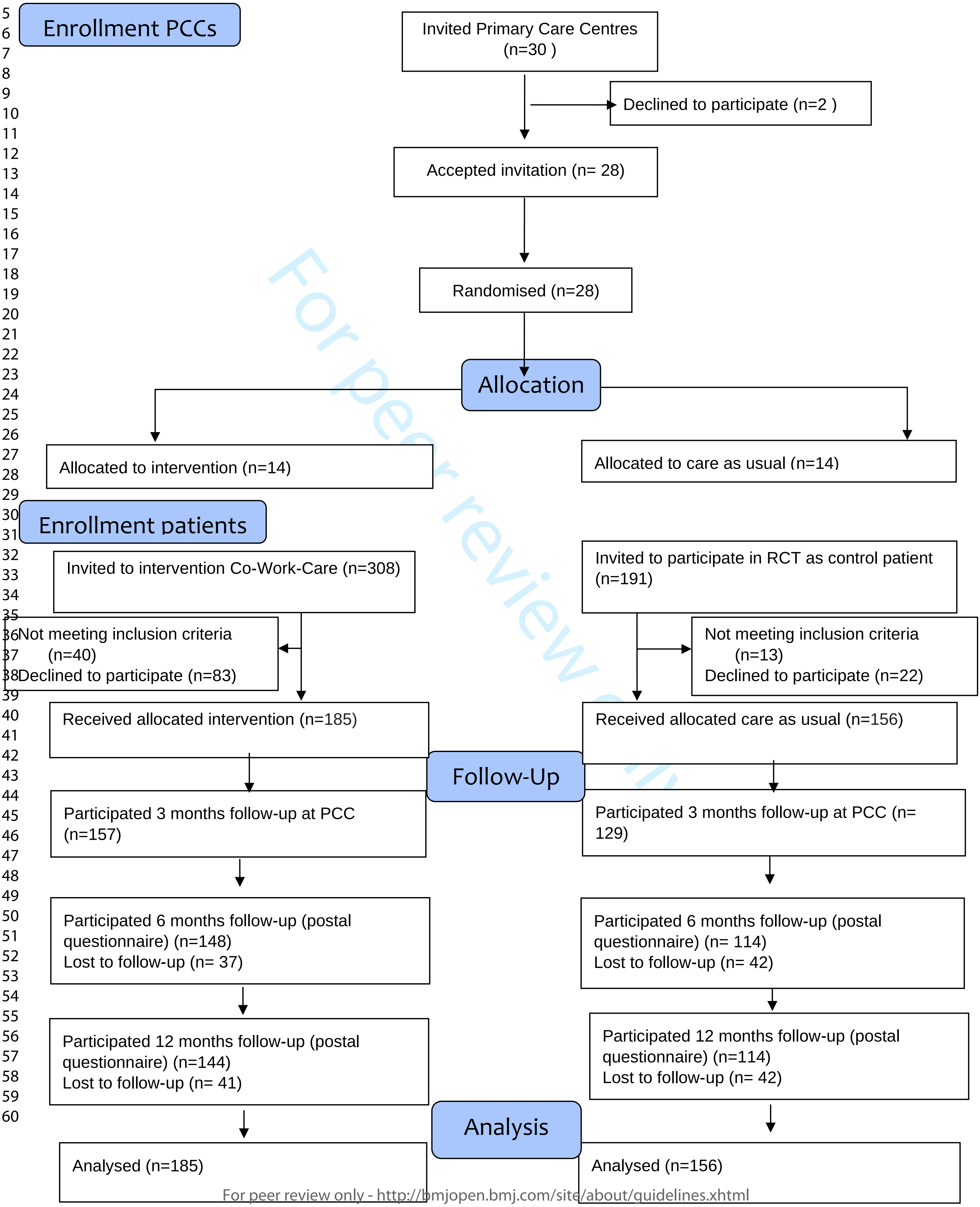

For peer review only

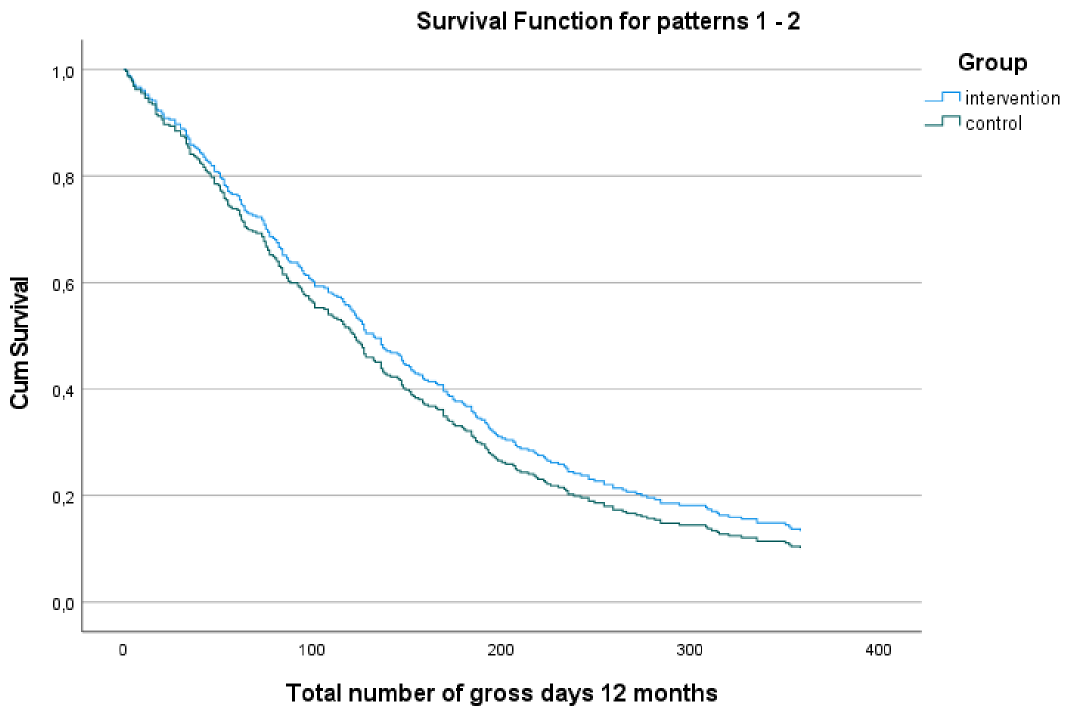

For peer review only

a)

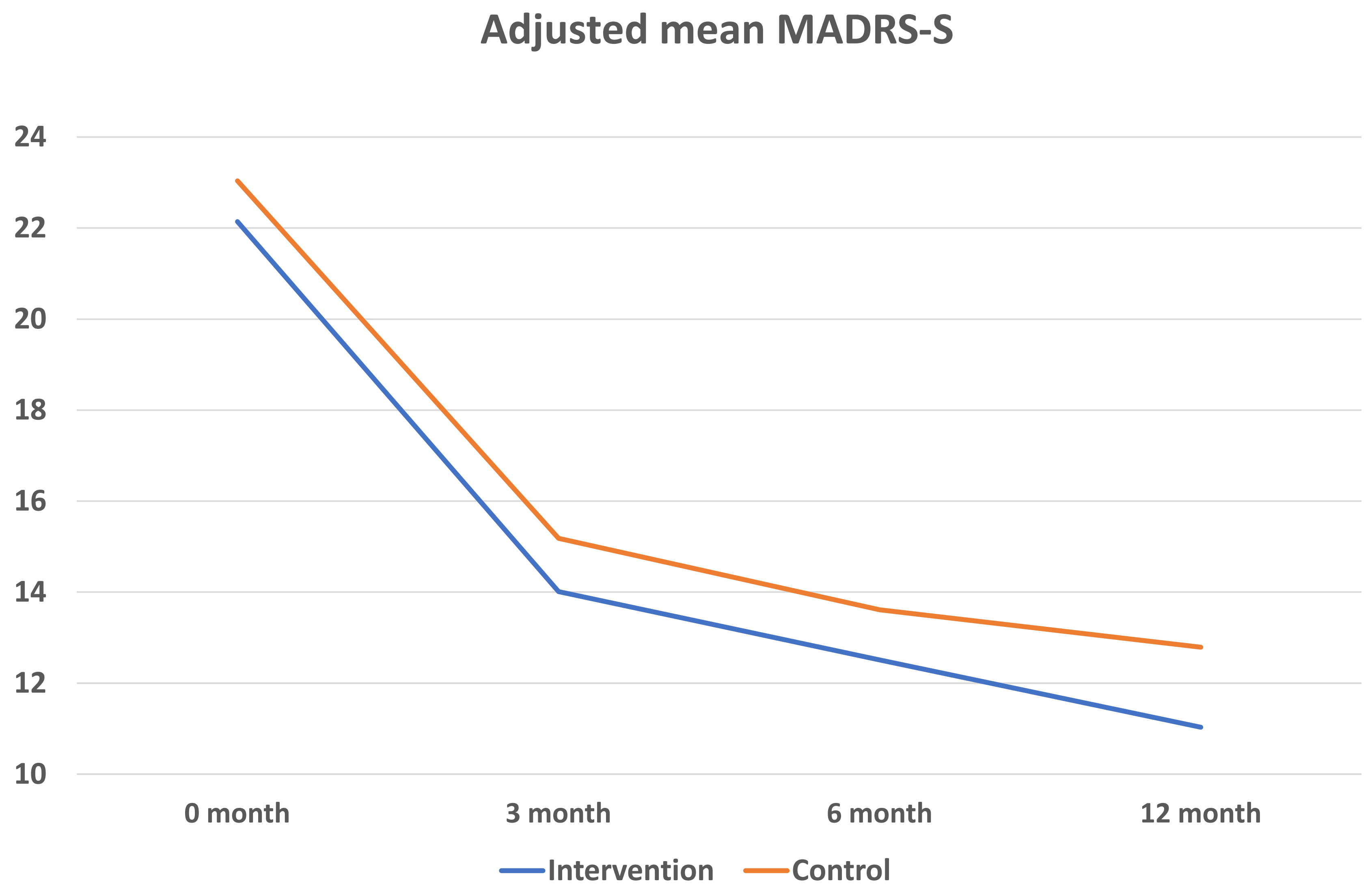
Adjusted mean MADRS-S
24
22
20
18
16
14
12
10
0 month
3 month
6 month
12 month
Intervention
Control

b)

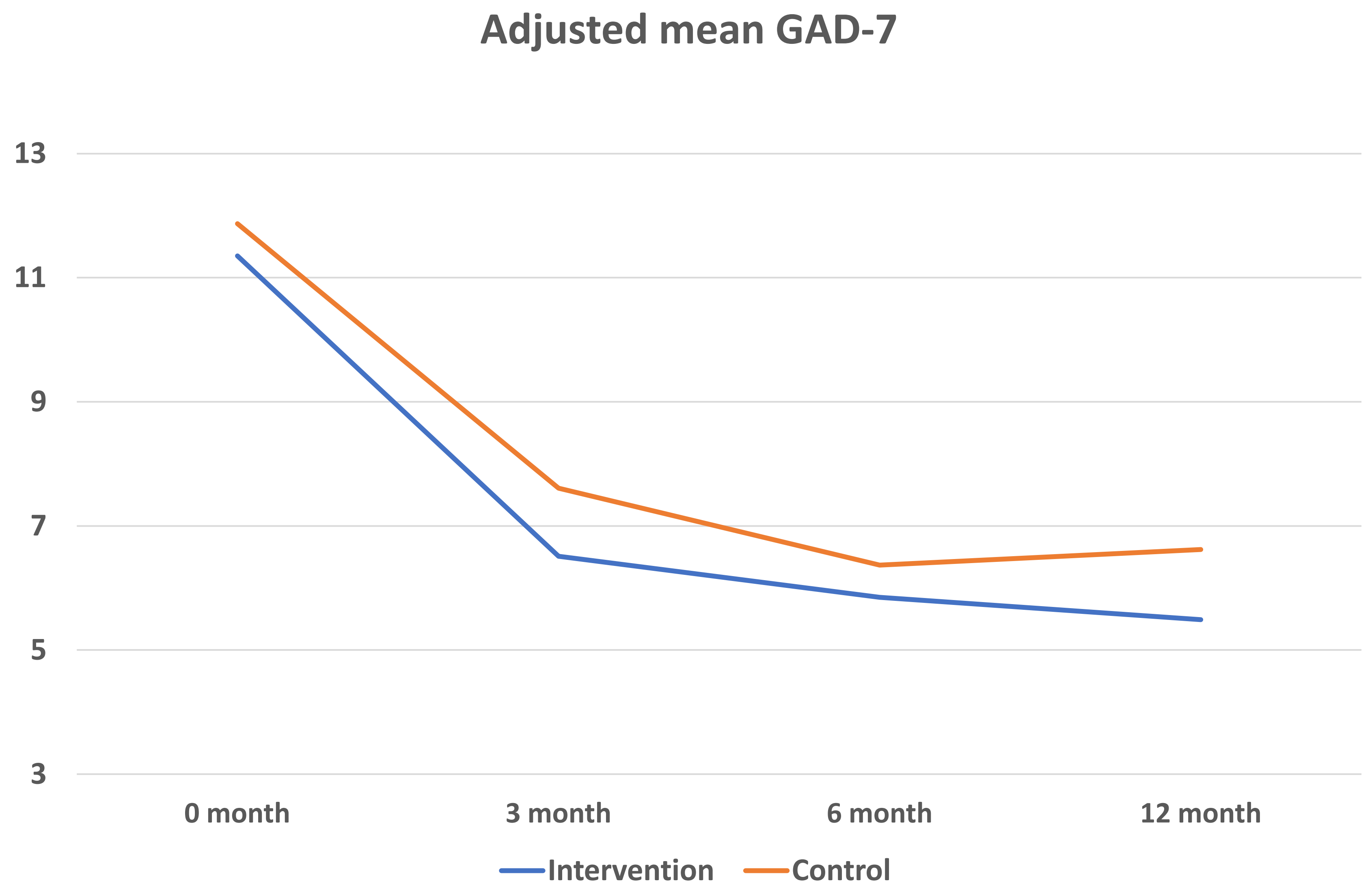
Adjusted mean GAD-7
13
11
9
7
5
3
0 month
3 month
6 month
12 month
Intervention
Control

c)

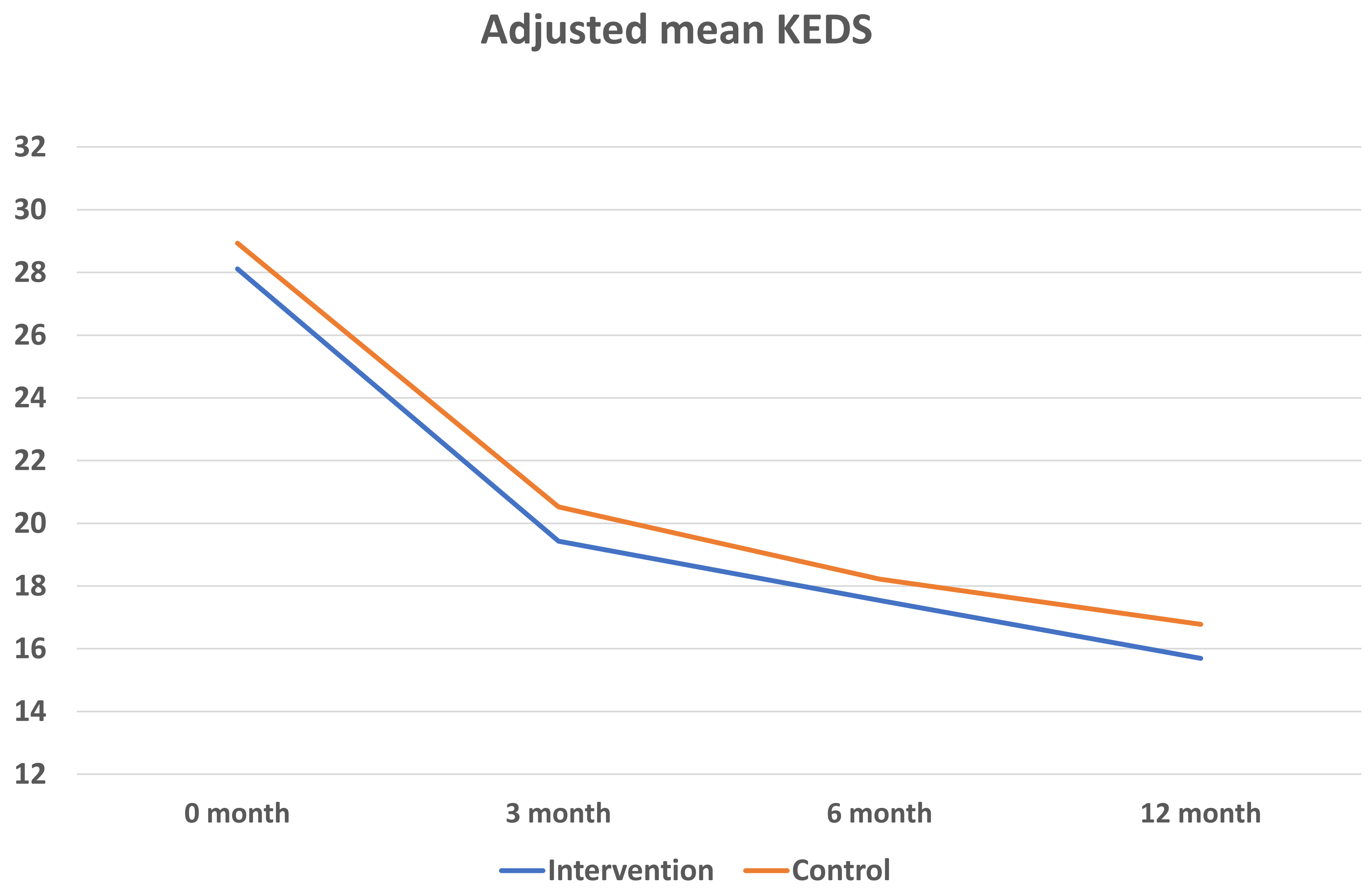
Adjusted mean KEDS
32
30
28
26
24
22
20
18
16
14
12
0 month
3 month
6 month
12 month
Intervention
Control

d)

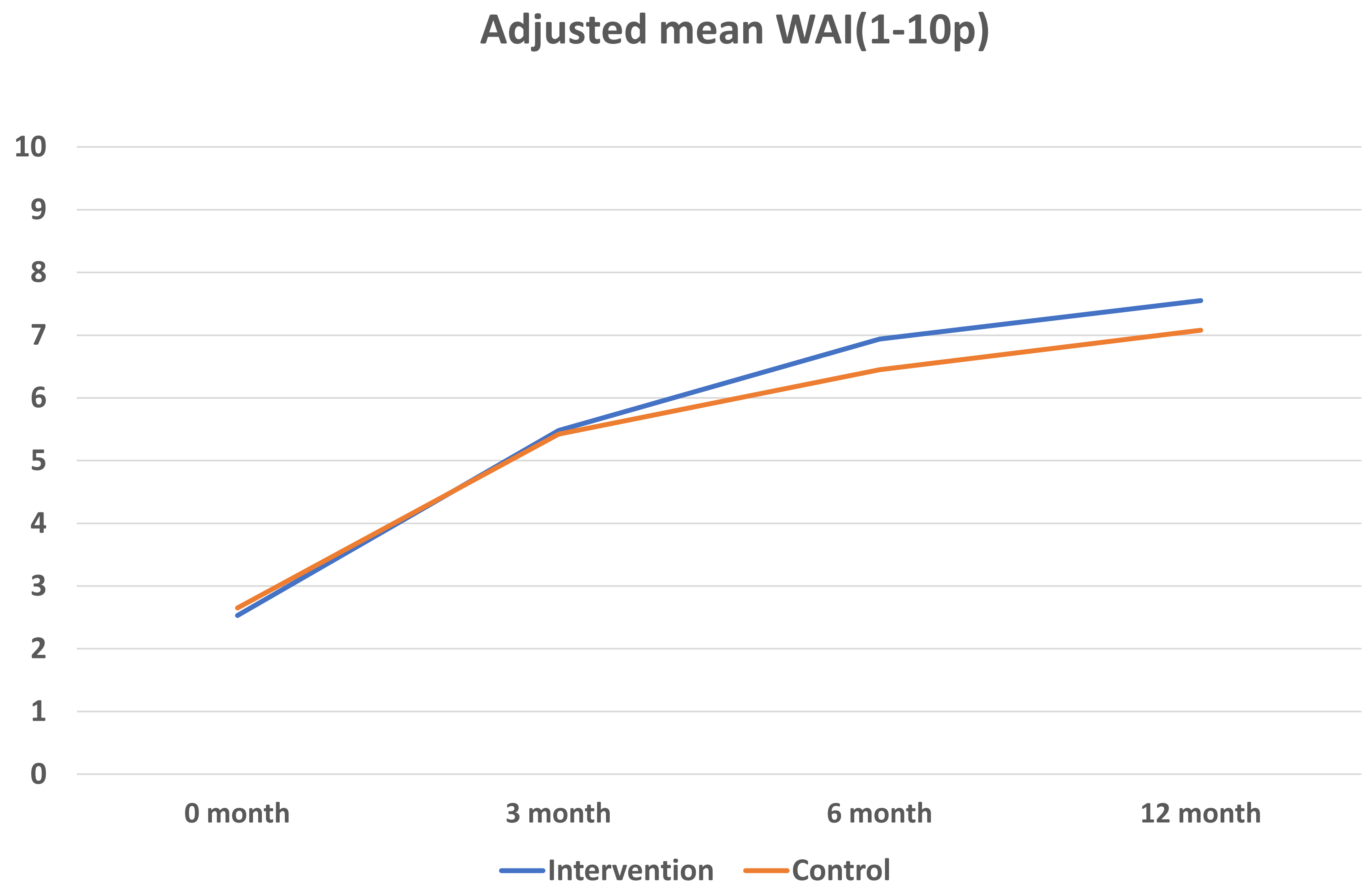
Adjusted mean WAI(1-10p)
10
9
8
7
6
5
4
3
2
1
0
0 month
3 month
6 month
12 month
Intervention
Control

e)

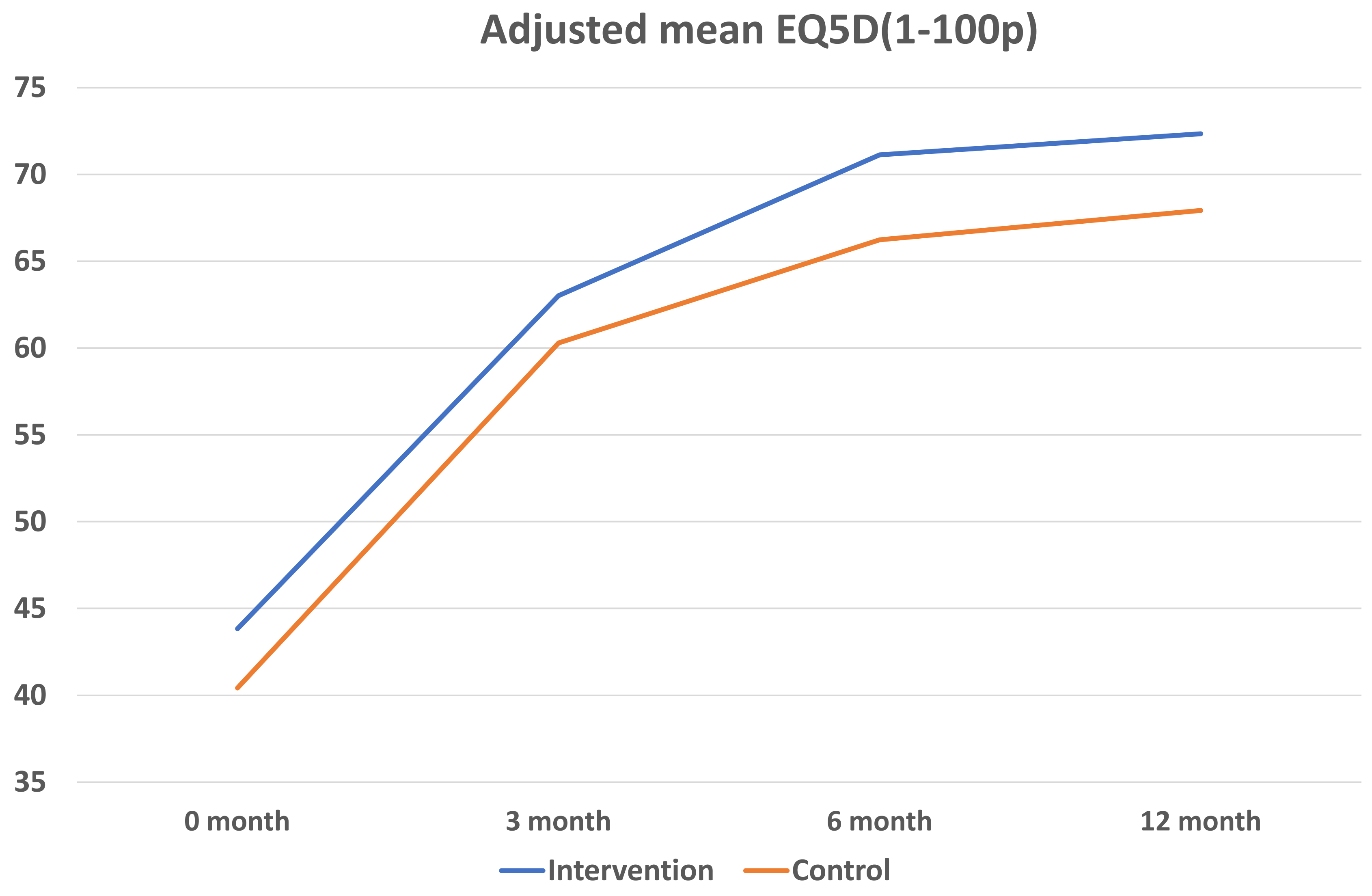
Adjusted mean EQ5D(1-100p)
75
70
65
60
55
50
45
40
35
0 month
3 month
6 month
12 month
Intervention
Control

a)

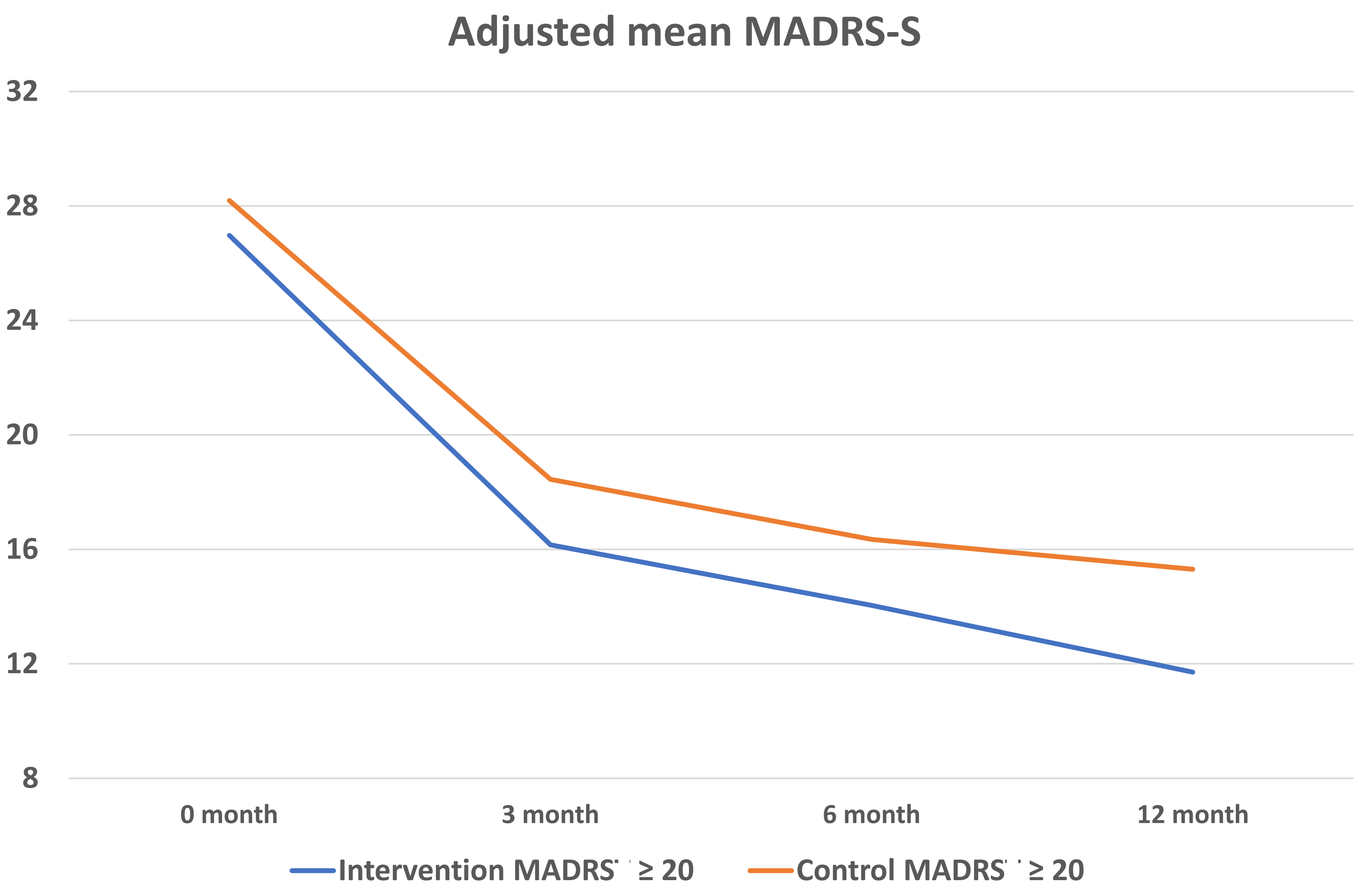
Adjusted mean MADRS-S
32
28
24
20
16
12
8
0 month
3 month
6 month
12 month
Intervention MADRS ≥ 20
Control MADRS ≥ 20

b)

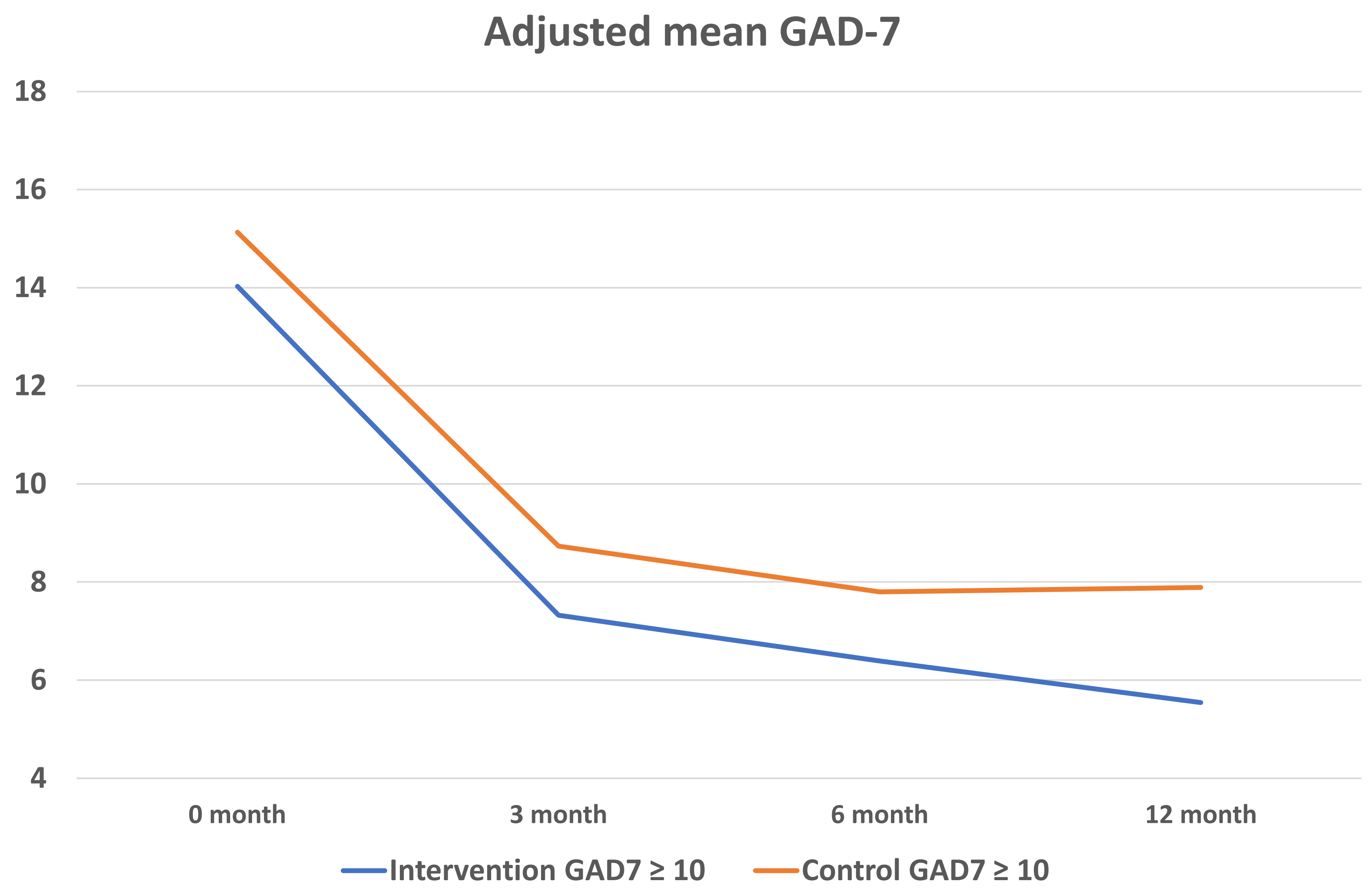
Adjusted mean GAD-7
18
16
14
12
10
8
6
4
0 month
3 month
6 month
12 month
Intervention GAD7 ≥ 10
Control GAD7 ≥ 10

c)

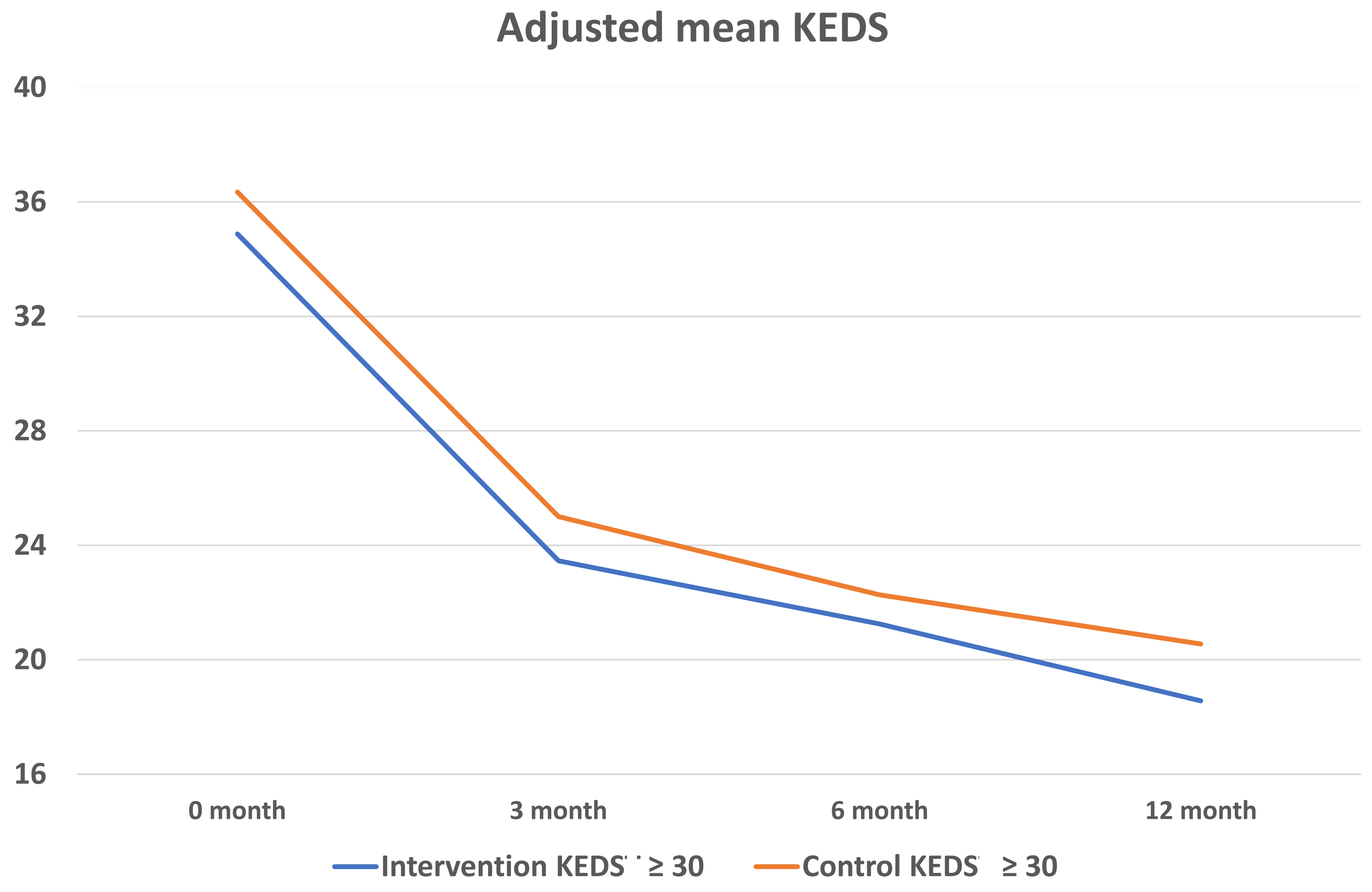
Adjusted mean KEDS
40
36
32
28
24
20
16
0 month
3 month
6 month
12 month
Intervention KEDS ≥ 30
Control KEDS ≥ 30

Supplementary table S-1. Pragmatic characteristics of trial elements in the Co-Work-Care trial (from https://rethinkingclinicaltrials.org/chapters/pragmatic-clinical-trial/pragmatic-elements-an-introduction-to-precis-2/)

**Trial element**	**Pragmatic characteristics in Co-Work-Care trial**
Research question	Whether enhanced and early cooperation plus early person centred dialogue is more effective in reducing total duration of sick-leave for primary care patients with reduced function because of CMD
Setting	Embedded in the routine primary care setting
Participants	Primary care patients with reduced function because of CMD
Intervention and comparator	Compares two **real-world treatments** using flexible protocols – care manager contact plus enhanced and early cooperation and early person centred dialogue vs care manager contact as usual
Outcomes	Endpoints **clinically relevant** to participants, funders, communities, and healthcare providers
Clinical importance	Purposely designed for making healthcare decisions in settings in which the intervention will be implemented and be used. Important clinical questions can be answered directly from trial results

For peer review only

CONSORT 2010 checklist of information to include when reporting a randomised trial*

Section/Topic	Item No	Checklist item	Reported on page No
**Title and abstract**			
	1a	Identification as a randomised trial in the title	1
	1b	Structured summary of trial design, methods, results, and conclusions (for specific guidance see CONSORT for abstracts)	2,3
**Introduction**			
Background and objectives	2a	Scientific background and explanation of rationale	4-6
	2b	Specific objectives or hypotheses	6
**Methods**			
Trial design	3a	Description of trial design (such as parallel, factorial) including allocation ratio	7-8
	3b	Important changes to methods after trial commencement (such as eligibility criteria), with reasons	
Participants	4a	Eligibility criteria for participants	8
	4b	Settings and locations where the data were collected	8
Interventions	5	The interventions for each group with sufficient details to allow replication, including how and when they were actually administered	8-10
Outcomes	6a	Completely defined pre-specified primary and secondary outcome measures, including how and when they were assessed	13
	6b	Any changes to trial outcomes after the trial commenced, with reasons	
Sample size	7a	How sample size was determined	15
	7b	When applicable, explanation of any interim analyses and stopping guidelines	
Randomisation:			
Sequence generation	8a	Method used to generate the random allocation sequence	8
	8b	Type of randomisation; details of any restriction (such as blocking and block size)	8-9
Allocation concealment mechanism	9	Mechanism used to implement the random allocation sequence (such as sequentially numbered containers), describing any steps taken to conceal the sequence until interventions were assigned	8-9
Implementation	10	Who generated the random allocation sequence, who enrolled participants, and who assigned participants to interventions	8, 11,12
Blinding	11a	If done, who was blinded after assignment to interventions (for example, participants, care providers, those	9

For peer review only

For peer review only - http://bmjopen.bmj.com/site/about/guidelines.xhtml

		assessing outcomes) and how	
	11b	If relevant, description of the similarity of interventions	8, 11,12, Table 1
Statistical methods	12a	Statistical methods used to compare groups for primary and secondary outcomes	14-15
	12b	Methods for additional analyses, such as subgroup analyses and adjusted analyses	14-15
**Results**			
Participant flow (a diagram is strongly recommended)	13a	For each group, the numbers of participants who were randomly assigned, received intended treatment, and were analysed for the primary outcome	16, Fig 1,
	13b	For each group, losses and exclusions after randomisation, together with reasons	Fig 1
Recruitment	14a	Dates defining the periods of recruitment and follow-up	16
	14b	Why the trial ended or was stopped	16
Baseline data	15	A table showing baseline demographic and clinical characteristics for each group	Table 2
Numbers analysed	16	For each group, number of participants (denominator) included in each analysis and whether the analysis was by original assigned groups	Table 2, Table 4
Outcomes and estimation	17a	For each primary and secondary outcome, results for each group, and the estimated effect size and its precision (such as 95% confidence interval)	Fig 2, Table 3 Table 5
	17b	For binary outcomes, presentation of both absolute and relative effect sizes is recommended	
Ancillary analyses	18	Results of any other analyses performed, including subgroup analyses and adjusted analyses, distinguishing pre-specified from exploratory	Supplement. Fig S1
Harms	19	All important harms or unintended effects in each group (for specific guidance see CONSORT for harms)	22
**Discussion**			
Limitations	20	Trial limitations, addressing sources of potential bias, imprecision, and, if relevant, multiplicity of analyses	25-26
Generalisability	21	Generalisability (external validity, applicability) of the trial findings	25
Interpretation	22	Interpretation consistent with results, balancing benefits and harms, and considering other relevant evidence	26,27
**Other information**			
Registration	23	Registration number and name of trial registry	3
Protocol	24	Where the full trial protocol can be accessed, if available	28
Funding	25	Sources of funding and other support (such as supply of drugs), role of funders	30

*We strongly recommend reading this statement in conjunction with the CONSORT 2010 Explanation and Elaboration for important clarifications on all the items. If relevant, we also recommend reading CONSORT extensions for cluster randomised trials, non-inferiority and equivalence trials, non-pharmacological treatments, herbal interventions, and pragmatic trials. Additional extensions are forthcoming: for those and for up to date references relevant to this checklist, see www.consort-statement.org.

For peer review only - http://bmjopen.bmj.com/site/about/guidelines.xhtml

For peer review only

For peer review only

For peer review only - http://bmjopen.bmj.com/site/about/guidelines.xhtml

Table 1

Checklist of items to include when reporting a cluster randomised trial (adaptations from standard guidelines in italic)

Paper section and topic	Item	Descriptor	page
**Title and abstract**			2
Design	1*	How participants were allocated to interventions (eg random allocation, randomised, or randomly assigned), specifying that allocation was based on clusters	2
**Introduction**			
Background	2*	Scientific background and explanation of rationale, including the rationale for using a cluster design	6
**Methods**			
Participants	3*	Eligibility criteria for participants and clusters and the settings and locations where the data were collected	8
Interventions	4*	Precise details of the interventions intended for each group, whether they pertain to the individual level, the cluster level, or both, and how and when they were actually administered	8-10
Objectives	5*	Specific objectives and hypotheses and whether they pertain to the individual level, the cluster level, or both	6
Outcomes	6*	Report clearly defined primary and secondary outcome measures, whether they pertain to the individual level, the cluster level, or both, and, when applicable, any methods used to enhance the quality of measurements (eg multiple observations, training of assessors)	13
Sample size	7*	How total sample size was determined (including method of calculation, number of clusters, cluster size, a coefficient of intracluster correlation (ICC or k), and an indication of its uncertainty) and, when applicable, explanation of any interim analyses and stopping rules	15
Randomisation:			
Sequence generation	8*	Method used to generate the random allocation sequence, including details of any restriction (eg blocking, stratification, matching)	8
Allocation concealment	9*	Method used to implement the random allocation sequence, specifying that allocation was based on clusters rather than individuals and clarifying whether the sequence was concealed until interventions were assigned	8-9
Implementation	10	Who generated the allocation sequence, who enrolled participants, and who assigned participants to their groups	11,12
Blinding (masking)	11	Whether participants, those administering the interventions, and those assessing the outcomes were blinded to group assignment. If done, how the success of blinding was evaluated	9
Statistical methods	12*	Statistical methods used to compare groups for primary outcome(s) indicating how clustering was taken into account, methods for additional analyses, such as subgroup analyses and adjusted analyses	14-15
**Results**			
Participant flow	13*	Flow of clusters and individual participants through each stage (a diagram is strongly recommended). Specifically, for each group report the numbers of clusters and participants randomly assigned, receiving intended treatment, completing the study protocol, and analysed for the primary outcome. Describe protocol deviations from study as planned, together with reasons	Fig 1
Recruitment	14	Dates defining the periods of recruitment and follow up	16

For peer review only - http://bmjopen.bmj.com/site/about/guidelines.xhtml

Paper section and topic	Item	Descriptor	page
Baseline data	15*	Baseline information for each group for the individual and cluster levels as applicable	Table 2
Numbers analysed	16*	Number of clusters and participants (denominator) in each group included in each analysis and whether the analysis was by intention to treat. State the results in absolute numbers when feasible (eg 10/20 not 50%)	Fig 1 Table 2 and 4
Outcomes and estimation	17*	For each primary and secondary outcome, a summary of results for each group for the individual or cluster level as applicable, and the estimated effect size and its precision (eg 95% confidence interval) and a coefficient of intracluster correlation (ICC or k) for each primary outcome.	Fig 2, Table 3 and 5
Ancillary analyses	18	Address multiplicity by reporting any other analyses performed, including subgroup analyses and adjusted analyses, indicating those prespecified and those exploratory	Suppl.fig S-1
Adverse events	19	All important adverse events or side effects in each intervention group	22
**Discussion**			
Interpretation	20	Interpretation of the results, taking into account study hypotheses, sources of potential bias or imprecision and the dangers associated with multiplicity of analyses and outcomes.	25, 26
Generalisability	21*	Generalisability (external validity) to individuals and/or clusters (as relevant) of the trial findings	25
Overall evidence	22	General interpretation of the results in the context of current evidence	27

- ↲* Addition to CONSORT guidelines 20012

For peer review only

BMJ Open

Rehabilitation cooperation and person-centred dialogue meeting for patients sick-listed for common mental disorders - 12 months follow-up of sick-leave days, symptoms of depression, anxiety, stress and work ability. A pragmatic cluster-randomised controlled trial from the CO-WORK-CARE project

Journal:	*BMJ Open*
Manuscript ID	bmjopen-2023-074137.R1
Article Type:	Original research
Date Submitted by the Author:	10-May-2023
Complete List of Authors:	Björkelund, Cecilia; University of Gothenburg Sahlgrenska Academy, Primary Health Care/School of Public Health and Community Medicine<br>Saxvik, Ausra; University of Gothenburg Institute of Medicine, Primary Health Care/ School of Public Health and Community Medicine<br>Svenningsson, Irene; University of Gothenburg Institute of Medicine, Primary Health Care/School of Public Health and Community Medicine<br>Petersson, Eva-Lisa; University of Gothenburg Institute of Medicine, School of Public Health and Community Medicine<br>Wiegner, Lilian; University of Gothenburg Institute of Medicine, School of Public Health and Community Medicine<br>Larsson, Maria; Institute of Neuroscience and Physiology, Sahlgrenska Academy, University of Gothenburg, Section for rehabilitation and health<br>Törnbom, Karin; University of Gothenburg, Primary Care/School of Public Health and Community Medicine; University of Gothenburg, Department of Social Work<br>Wikberg, Carl; University of Gothenburg, Primary Health Care Department of Public Health and Community Medicine<br>Ariai, Nashmil; University of Gothenburg, Primary Health Care Department of Public Health and Community Medicine<br>Nejati, Shabnam; University of Gothenburg, Primay Health Care/School of Public Health and Community Medicine<br>Hensing, Gunnel; University of Gothenburg Institute of Medicine, School of Public Health and Community Medicine<br>Hange, Dominique; University of Gothenburg, Primary Health Care/School of Public Health and Community Medicine
**Primary Subject Heading**:	General practice / Family practice
Secondary Subject Heading:	Mental health, Patient-centred medicine
Keywords:	Depression & mood disorders < PSYCHIATRY, Anxiety disorders < PSYCHIATRY, Primary Health Care, Clinical Trial, Patient-Centered Care, REHABILITATION MEDICINE

SCHOLARONE™
Manuscripts

I, the Submitting Author has the right to grant and does grant on behalf of all authors of the Work (as defined in the below author licence), an exclusive licence and/or a non-exclusive licence for contributions from authors who are: i) UK Crown employees; ii) where BMJ has agreed a CC-BY licence shall apply, and/or iii) in accordance with the terms applicable for US Federal Government officers or employees acting as part of their official duties; on a worldwide, perpetual, irrevocable, royalty-free basis to BMJ Publishing Group Ltd ("BMJ") its licensees and where the relevant Journal is co-owned by BMJ to the co-owners of the Journal, to publish the Work in this journal and any other BMJ products and to exploit all rights, as set out in our licence.

The Submitting Author accepts and understands that any supply made under these terms is made by BMJ to the Submitting Author unless you are acting as an employee on behalf of your employer or a postgraduate student of an affiliated institution which is paying any applicable article publishing charge ("APC") for Open Access articles. Where the Submitting Author wishes to make the Work available on an Open Access basis (and intends to pay the relevant APC), the terms of reuse of such Open Access shall be governed by a Creative Commons licence – details of these licences and which Creative Commons licence will apply to this Work are set out in our licence referred to above.

Other than as permitted in any relevant BMJ Author's Self Archiving Policies, I confirm this Work has not been accepted for publication elsewhere, is not being considered for publication elsewhere and does not duplicate material already published. I confirm all authors consent to publication of this Work and authorise the granting of this licence.

For peer review only

Rehabilitation cooperation and person-centred dialogue meeting for patients sick-listed for common mental disorders - 12 months follow-up of sick-leave days, symptoms of depression, anxiety, stress and work ability. A pragmatic cluster-randomised controlled trial from the CO-WORK-CARE project

Cecilia Björkelund [1,2], Ausra Saxvik [1], Irene Svenningsson [1,2], Eva-Lisa Petersson [1,2], Lilian Wiegner [1,3], Maria EH Larsson [2,4], Karin Törnbom [1,5], Carl Wikberg [1,2], Nashmil Ariai [1,2], Shabnam Nejati [1,2], Gunnel Hensing [6], Dominique Hange [1,2]

[1] Primary Health Care/Department of Public Health and Community Medicine, Institute of Medicine, Sahlgrenska Academy, University of Gothenburg

[2] Research, Education, Development & Innovation, Primary Health Care, Region Västra Götaland, Sweden

[3] Institute of Stress Medicine, Region Västra Götaland, Sweden

[4] Unit of Physiotherapy/ Department of Health and Rehabilitation, Institute of Neuroscience and Physiology, Sahlgrenska Academy, University of Gothenburg, Gothenburg, Sweden

[5] Department of Social Work, University of Gothenburg, Gothenburg, Sweden

[6] Social Medicine/ Department of Public Health and Community Medicine, Institute of Medicine, Sahlgrenska Academy, University of Gothenburg

Corresponding author

Cecilia Björkelund, Primary Health Care/Department of Public Health and Community Medicine

Box 453

41530 Gothenburg, Sweden cecilia.bjorkelund@allmed.gu.se

For peer review only - http://bmjopen.bmj.com/site/about/guidelines.xhtml

Abstract

Objectives To study whether early and enhanced cooperation within the primary care centres combined with workplace cooperation via a person-centred employer dialogue meeting can reduce days on sick-leave compared with usual care manager contact for patients on sick-leave because of common mental disorders (CMD). Secondary aim: to study lapse of CMD symptoms, perceived work ability (WAI) and quality of life (QoL) during12 months.

Design Pragmatic cluster-randomised controlled trial, randomisation at primary care centre (PCC) level.

Setting 28 PCCs in Region Västra Götaland, Sweden, with care manager organisation.

Participants 30 PCCs were invited, 28 (93%) accepted invitation (14 intervention, 14 control) and recruited 341 patients newly sick-listed because of CMD (n=185 at intervention, n=156 at control PCCs).

Intervention. Complex intervention consisting of i) early cooperation among GP, care manager and a rehabilitation coordinator, plus ii) a person-centred dialogue meeting between patient and employer within 3 months. Control group: regular contact with care manager.

Main outcome measures 12 months net and gross number of sick-leave days at group level. Secondary outcomes: 12 months depression, anxiety, stress symptoms, perceived work ability (WAI) and Quality of Life (EQ-5D).

Results No significant differences were found between intervention and control groups concerning days of sick-leave (intervention net days of sick-leave mean 102.48 (SE 13.76) vs control 96.29 (SE 12.38) p=0.73), return to work (hazard ratio=0.881, 95% CI 0.688 to 1.128), or CMD symptoms, WAI or EQ-5D after 12 months.

Conclusions It is not possible to speed up CMD patients' return to work or to reduce sick-leave time by early and enhanced coordination among GP, care manager and a rehabilitation

For peer review only - http://bmjopen.bmj.com/site/about/guidelines.xhtml

coordinator, combined with early workplace contact over and above what "usual" care manager contact during 3 months provides.

Strengths and limitations of this study

- The Co-Work-Care trial was accomplished entirely in primary care.
- The intervention was adapted to a level possible to conduct in a Swedish primary care context with low level of resources for direct workplace intervention and cooperation.
- The intervention model was person-centred and emphasised the individual's perceptions of workplace conditions facilitating own return-to-work.
- Covid19 could have had possible impact on the last months of follow-up for 12 months sick-leave duration, however, this effect should have been comparable in the control group.
- The complexity of the intervention based on the individual patient's preferences, needs and values, a structured cooperation among GP, care manager and rehabilitation coordinator and a person-centred dialogue meeting with employer could be called into question concerning possible biasing factors.

Trial registration ClinicalTrials.gov Identifier: NCT03250026 First Posted: August 15, 2017. Start of recruitment of PCCs: September, 2017. Start of inclusion of patients: December, 2017

For peer review only - http://bmjopen.bmj.com/site/about/guidelines.xhtml

Background

The largest diagnostic group among sick-listed women and men in Sweden is common mental disorders (CMD), i.e., depression, anxiety syndromes and stress-related mental disorder (adjustment disorder, exhaustion disorder) (1). The yearly cumulative incidence is approximately 3% of the labour force (1). Likewise, in other parts of Europe CMD are common among persons who are sick-listed, and work-related stress is increasingly identified as a contributing cause (2,3,4,5). In fact, more than 50% of new sick-leave cases in Sweden have adjustment disorder or exhaustion disorder (ICD F43) as their certification diagnosis (1, 3), while depression disorder has decreased as certification diagnosis (1). Many of these individuals return to work after treatment, but for some return to work is slower. Knowledge of the most decisive factors for continuing sick-leave is to a great extent lacking for CMD patients today (6), but reasons include symptom severity and difficulties to adapt workplace or work tasks (6,7). Long sick-leave periods (>12 weeks) are common in Sweden, and most intervention studies have so far failed to show marked positive effects on return to work (RTW), except for interventions including increased contact with the workplace (7, 8). Prolonged RTW can lead to a more permanent exclusion from the labour market, and better methods are needed to prevent an excessively long sick-leave period that could possibly be counter-productive.

Since the largest proportion of individuals with mental illness seek primary care, usually with symptoms that manifest in depressive and/or anxiety symptoms, primary health care centres are a relevant arena for interventions (9). An organisation guided by collaborative care principles, in which a care manager coordinates care at the primary care centre (PCC) by maintaining a close and regular contact with patients and aligns efforts for their individual needs, has shown good results regarding mental and physical health improvements (10, 11, 12). In a recent systematic review, a care manager organisation at the PCC was one of few

For peer review only

factors found to increase RTW frequency when compared to already existing forms of organisation and actions (12). Other outcomes related to team-based interventions at the PCC were increased quality of care, reduction of symptoms and increased work ability, function and quality of life (9, 10, 11, 12, 13, 14). A care manager organisation seems to enhance care approaches that allow complexity, person-centredness and interaction (10,15). The collaborative care organisation handles person-centredness and complexity via the care manager, who also facilitates interaction with other psycho-social competencies, especially a psychologist/psychotherapist for the individual patient (in the Swedish study around 45-50% (10)), but also a counsellor, physiotherapist and other contacts at the PCC when needed. However, beneficial effects have primarily been shown for primary care patients with depressive illness. Thus far, evidence is lacking both concerning the entire CMD diagnosis spectrum and especially for those with a symptom burden and work ability reduction high enough to necessitate sick-leave.

In an earlier Swedish study, PRIM-CARE RCT (ClinicalTrials.gov Identifier: NCT02378272), a care manager intervention at the primary care centre improved return to work and reduced sick-leave for patients with depression compared to care as usual (10). Consequently, a wide implementation of a care manager organisation at interested PCCs has been going on since 2015 in the Region Västra Götaland, Sweden. The present Co-Work-Care trial was initiated to examine whether a care manager contact combined with early structured enhanced cooperation among the care manager, rehabilitation coordinator and general practitioner (GP) at the PCC plus a person-centred dialogue meeting with the patient’s employer would further increase and shorten RTW for CMD patients on sick-leave compared to those with “usual” care manager contact.

For peer review only - http://bmjopen.bmj.com/site/about/guidelines.xhtml

Objectives

The primary aim was to study whether an early structured enhanced cooperation among the care manager, rehabilitation coordinator and GP combined with workplace cooperation via a person-centered dialogue meeting with the patient's employer can reduce sick-leave time and increase RTW and work ability compared with care manager contact "as usual". A secondary aim was to study lapse of depression, anxiety and stress-related symptoms as well as perceived work ability and quality of life during 12 months. Thus, we regarded it as important also to examine the lapse of mental symptoms to study the effects of the intervention on the patient's mental state. As the intervention was based on structured enhanced cooperation between several staff members within the PCC, randomisation at PCC level was chosen.

Methods

Study setting and context

As described above, an implementation of the care manager function at interested PCCs has been ongoing since 2015 in the Region Västra Götaland, Sweden (inhabitants ~ 1.7 million; ~ 200 PCCs). The first part of the implementation was conducted as a randomised controlled trial (RCT) (ClinicalTrials.gov Identifier: NCT02378272) (10). Since the RCT showed positive effects concerning depression course, quality of life (QoL) and RTW (10, 14, 15), a care manager (mostly a registered nurse) for patients with CMD including stress-related disorder (adjustment disorder + exhaustion disorder) was implemented at most PCCs in the region (at around 160 PCCs). The implementation of a care manager collaborative care organisation for a team-based care was also recommended for national implementation in the Swedish guidelines for care of depression and anxiety syndromes (16).

For peer review only - http://bmjopen.bmj.com/site/about/guidelines.xhtml

In Sweden, primary care is organised in primary care centres (PCCs), which (besides several GPs) also have specially educated nurses, primary care psychologists, counsellors and physiotherapists, often working in team around the patient. The PCCs serve around 2,000-30,000 listed patients (around 1 GP and 2 nurses per 2,000 listed). All PCCs have, for example, a specially educated nurse who works part-time with diabetes patient care together with the GP, preferably via individually adjusted health promotion activities, while also guaranteeing accessibility and continuity for the diabetic patient. In a similar manner, the care manager's assignment is to work part-time with CMD patients to guarantee accessibility and continuity for the CMD patient and promote psycho-pedagogical support to the patient during the illness. A care manager academic education, financed via the Region Västra Götaland county council, is mandatory (Care Manager for CMD, 7.5 higher education credits).

During the same time as the care manager organisation was implemented, an implementation of a function to coordinate the sick-leave and rehabilitation process, i.e. a rehabilitation coordinator, was also carried out at all PCCs in the region. A rehabilitation coordinator was initiated in 2016 via a national project with special governmental funding to the regions to offer coordinating work to patients on sick-leave to promote return to work. A rehabilitation coordinator has an education in social insurance laws and regulations and vocational rehabilitation and serves as a regulation knowledge broker for the PCCs and a support for the patient's RTW process and workplace cooperation. The education standards vary between regions and organisations. In primary care, the rehabilitation coordinator often serves several PCCs in contrast to care managers who are part of the PCCs' ordinary personnel and working part of their duty as care managers. At some PCCs enrolled in this trial (11/28=39 %; 5/14 intervention PCCs, 6/14 control PCCs) the care manager also served as rehabilitation coordinator.

For peer review only - http://bmjopen.bmj.com/site/about/guidelines.xhtml

Trial design

The design was a pragmatic cluster-randomised controlled trial with complex intervention and thus randomisation was performed at PCC level (supplemental Table S1). The study adheres to Consort guidelines with extension to cluster randomized trials and the Swedish Ethics Review Board approved the study.

Participants

Invitation to PCCs to participate in a randomised controlled trial started in September 2017, and 30 PCCs in the Västra Götaland Region with an established organisation consisting of a care manager and affiliated rehabilitation coordinator were invited via the regional primary care R&D support organisation to take part in the CO-WORK-CARE study. Of the invited 30 PCCs, 28 (93%) PCCs accepted the invitation. All PCCs were informed about the trial both via information visits to every PCC before study start and by written confirmation which was obtained from every PCC, both intervention and control PCCs. All PCCs participated in educational activities concerning study protocol (figure 1, upper part). Thus, at all PCCs in the RCT, intervention as well as control, a care manager was working part-time at the PCC and a rehabilitation coordinator was affiliated.

Randomisation and masking

Randomisation was conducted at PCC level. A total of 28 primary care centres were included and divided into two strata, rural (n=14) and urban (n=14). The centres in each stratum were then divided into seven blocks of two centres each. One centre in each block was randomised to the intervention group (n=14) and one to the control group (n=14) by the Primary Health Care R&D Region Västra Götaland, Sweden (figure 1). Cluster randomisation was chosen to avoid the treatment contamination between the intervention and control groups that would

For peer review only - http://bmjopen.bmj.com/site/about/guidelines.xhtml

For peer review only

occur if patients at centres were individually randomised. No masking was possible, either concerning personnel at PCCs or research personnel.

Patients

All newly attending patients at intervention and control PCCs who were judged in need of new sick-leave due to mild/moderate depression (ICD F32, F33), anxiety syndrome (ICD F40, F41, F48) and/or stress-related mental disorder (ICD F43) (excluding post-traumatic stress disorder) were invited by the GP or the care manager to participate in the trial. Diagnosis was established by the GP, who made out the sickness certificate, and the diagnosis was confirmed by use of PRIME-MD, a diagnostic tool for depression and anxiety disorders especially for primary care (17), and by results from the KEDS assessment instrument (not diagnostic) (18,19).

Exclusion criteria

Patients diagnosed with serious depression, bipolar disorder, psychosis, addiction, pregnancy > 1st trimester, cognitive impairment, post-traumatic stress syndrome or not speaking/understanding Swedish were excluded.

Intervention

The CO-WORK-CARE complex intervention consisted of i) early structured cooperation among care manager, rehabilitation coordinator and GP, and ii) a person-centred dialogue meeting between patient and employer (immediate supervisor) with the rehabilitation coordinator as facilitator.

The care for the patient was based on the patient's ongoing sick-leave, the sick-leave diagnosis, the individual care plan (which the care manager and the patient initially drew up together) and the course of the CMD symptoms. In both intervention and control arms the individual care plan was drawn up between care manager and patient, but at intervention PCCs the rehabilitation coordinator was included from start, and the care manager, the

For peer review only - http://bmjopen.bmj.com/site/about/guidelines.xhtml

rehabilitation coordinator and the GP continuously had contact about the lapse of the patient's illness and progress, see table 1. The intervention also consisted of a person-centred dialogue meeting between the employer and employee with the rehabilitation coordinator as the guide and facilitator, effectuated within 3 months from study start, as early as possible based on the patient's condition and opinion.

Table 1. Care Process in the Co-Work-Care trial. Grey parts common for control and intervention PCCs, green parts solely for intervention PCCs.

Actor	GP	Care Manager	Rehabilitation coordinator	Dialogue meeting
**Action**	**Diagnoses CMD**	**Continuous contact from start sick-leave certification**	**Initiates return to work**	**Within 12 weeks**
**First contact**	Sick-leave certification >14 days with CMD diagnosis.<br>Somatic examination<br><br>Information to care manager (and other staff when assessed needed)	Care plan together with patient based on interview and anamnesis.<br><br>Assessment with MADRS-S<br>GAD7<br>KEDS<br><br>Information to other staff when therapy needed	Interview with patient when care manager indicates patient ready to start rehabilitation process.<br><br>Time for dialogue meeting agreed upon with patient.<br>Dialogue meeting decided.	Person-centred dialogue meeting between patient and employer;<br>Rehabilitation coordinator as the dialogue moderator.<br>Patient explains her/his view on return to work process
**Follow up**	Evaluates need of further sick-leave in regular reappointments	Telephone follow-up week 1,2,4,8,12<br><br>When patient ready: work questions discussed	Telephone contact with employer for interview about patient's work situation and information about dialogue meeting scope	
**Ongoing**	*Structured cooperation between GP, care manager and rehabilitation coordinator*			

The purpose of the person-centred dialogue meeting was above all to make it possible for the patient to inform the employer about the patient's own conception of factors that could facilitate a return to work. The dialogue meeting was a simplified dialogue meeting applied to primary care context, adapted from a more extensive protocol by Karlson and Östberg (20). The dialogue meeting was person-centred and focused on the needs of the individual patient and the patient's preferences, needs and values in the return-to-work (RTW) process (21). A person-centred dialogue meeting between the employer and the patient is not utilised in the Swedish workplace rehabilitation. Regulations state that a meeting among the workplace representative, the Social Insurance Agency, the PCC representative (most often GP, sometimes also rehabilitation coordinator) and the patient should be initiated after around 3 months of (full-time) sickness absence. This regulated meeting is not a person-centred meeting where the patient has the opportunity to describe her/his experience of the illness and thoughts about how to return to work at the workplace. In the CO-WORK-CARE study's person-centred dialogue meeting (where only three participants are present), the rehab coordinator was the mentor and guarantee for keeping the patient in the centre.

In all patient care, there was also a cooperation with the general practitioner (GP) and with the PCC's psycho-social team, but the structured and early care manager/rehabilitation coordinator /GP cooperation as well as the person-centred dialogue meeting with the employer was the added value in the intervention.

Procedures

Intervention PCCs

For the intervention PCCs an extended instruction about the complex intervention with early cooperation and person-centred dialogue meeting was conducted (8 hour session for the entire medical care personnel group including rehabilitation coordinator). During the RCT, each intervention PCC was regularly supported by a research team specialised

For peer review only

nurse/counsellor to secure adherence to the complex intervention. The complex intervention with early cooperation and person-centred dialogue meeting was added to the care manager care-as-usual function provided at the control PCCs, which already included engagement of psychologist/psychotherapist as well as other members of the psychosocial team according to national guidelines and focus on the patient's symptom development and preferences. Early cooperation was carried out by i) information from the care manager to the rehabilitation coordinator about the patient's status and care planning concerning workplace contact in conjunction with the care manager's and patient's initial meeting, and ii) in addition to care-as-usual team meetings with the psycho-social team when psychologist/other staff were engaged, regular meetings among the care manager, GP and rehabilitation coordinator when the care manager indicated that the patient was ready to start rehabilitation process (table 1).

Control PCCs

At the control PCCs, care as usual (CAU) consisted of: structured care manager function at the PCC with care manager contact with the patient during 3 months, as described in the PRIM-CARE trial (10). The care manager function included an initial 1 hour meeting between patient and care manager, where a care plan was established adapted to the patient's condition and preferences, and an agreement about the communication between patient and care manager during the next 12 weeks, mainly contacts by telephone, was drawn up (Table 1). The care manager contact was a way of increasing accessibility and continuity for the patient and was provided in addition to other therapeutic or pharmacologic treatment according to the patient's needs (10). This involved the engagement of a psychologist/ psychotherapist in around every second patient, as well as other members of the psychosocial team when needed (10). Contacts with GP and other competencies were provided according to the needs of the individual patient. Contact with rehabilitation coordinator was often only made in case of long sick-list duration (usually >2 months).

For peer review only - http://bmjopen.bmj.com/site/about/guidelines.xhtml

Control PCCs were also supported concerning patient recruitment and follow-up.

Patient and public involvement

Patients and/or the public were not involved in the design, conduct, or reporting, or dissemination plans of this research.

Outcomes

Primary outcomes: Number of sick-leave days (collected via the Micro-Data for Analysis of the Social Insurance System (MiDAS database)) after 12 months at group level (intervention vs control patients) for comparison. Proportion of patients who returned to full time work, time until full RTW, number/proportion of patients on sick-leave at 3, 6 and 12 months.

Secondary outcomes: Patient-related outcome measures with self-assessment instruments MADRS-S (22), GAD-7 (23), KEDS (18), EQ-5D (24) and WAI (25) course. Remission according to MADRS-S (≤12), GAD ≤ 4, KEDS (≤18), frequency of antidepressant therapy, frequency of psychotherapeutic contacts.

Self-assessment instruments used for PROMs

Depression was measured with the *Montgomery Asberg Depression Rating Scale-Self* (MADRS-S), which includes rating of mood, feelings of unease, sleep, appetite, ability to concentrate, initiative, emotional involvement, pessimism and zest for life (22). Every item is rated from 0 to 6 points with a total of 54 points where points 0-12 are classified as no depression, 13-19 points as mild depression, 20-34 points as moderate depression and >34 points as severe depression (22). We included individuals with ≤ 34 points.

For peer review only - http://bmjopen.bmj.com/site/about/guidelines.xhtml

Anxiety was measured by the *General Anxiety Disorders Scale-7* (GAD-7) (23). The GAD-7 consists of 7 items. Each question gives 0-3 points with a total of 21 points, classified as follows: >4 as mild GAD, >9 as moderate GAD and >14 as severe GAD (23).

Exhaustion disorder was measured by the *Karolinska Exhaustion Disorder Scale* (KEDS) (18). KEDS is a self-rating scale which consists of nine items, each ranging from 0 to 6 points with a scale range of 0-54, where points >18 indicate an increased risk for exhaustion disorders (18).

To measure health-related quality of life, the *EuroQoL-5D* (EQ-5D) five-dimensional questionnaire was used, measuring health status with one question for each of the following five dimensions: mobility, self-care, usual activities, pain/discomfort, and anxiety/depression (24).

Work ability was measured by the *Work Ability Index* (WAI) (25). WAI is a summary measure of seven items (range 7-49): current work ability, work ability in relation to the demands of the job, number of current diseases, estimated work impairment, sick-leave during the past year, own prognosis of work ability and mental resources (25).

Statistics

Standard statistical methods were used for descriptive statistics. Continuous variables were analysed by independent sample t-test or Mann-Whitney U test and categorical variables and frequencies by Pearson chi-square test. Linear mixed model analysis with repeated measures was used to compare means of intra-individual change in depressive, anxiety and stress symptoms and QoL scores between the intervention group and the control group. These analyses were adjusted for cluster randomisation of PCC, repeated measures for every person and pre-specified variables age, sex, education, and antidepressants at inclusion. In the model were a repeated fixed effect for ”time” for each ”person” and “center” and a random intercept

For peer review only - http://bmjopen.bmj.com/site/about/guidelines.xhtml

for each "center" included. These analyses to control for correlation in repeated measures for each "person" and "center" and to adjust for cluster randomisation of PCC. Linear mixed model analysis was performed for comparison of total number of 12 months sick-leave days between intervention and control group and were adjusted for cluster randomisation of PCC, age, sex, education and antidepressants at inclusion. A random intercept for each "center" was included in the model adjusting for cluster randomisation of PCC. EMMEANS subcommand from Mixed model in SPSS were used to compare estimated marginal means between the intervention group and the control group for every time in the study. Cox regression analysis was done to study RTW between the intervention and the control group during 12 months of sick-leave with control of the variables age, sex and education with no adjustment for cluster randomisation of PCC.

The statistical analyses were made using statistical software SPSS, version 28 and SAS, version 9.4. Statistical significance was set at $p<0.05$. No multiple adjustments were considered.

Power Calculation

The trial was designed to achieve adequate power for the primary outcome. Based on a recently accomplished RCT study (10) with randomisation at the PCC level, where a significant difference in RTW could be identified in a group of 83 vs 88 patients on sick-leave at baseline in intervention and control groups, we made the assumption that 150 patients in each group would be sufficient for reaching a power of 80% to detect a difference of 3 units between the two groups with a significance level of 10%. The underlying assumption was a standard deviation in the group of 10 units, a within-subject correlation of 0.4, and a within-cluster correlation of 0.1, i.e. a design effect of 1.9 to correct for having a cluster analysis.

For peer review only - http://bmjopen.bmj.com/site/about/guidelines.xhtml

To be able to conduct analyses for the group of patients with stress-related mental disorder (adjustment disorder, exhaustion disorder), patients with F43 as main or contributing diagnosis were oversampled during 2020-02-01 to 2020-06-30.

Results

Inclusion of patients started December 2017 and was completed end of June 2020. In total 499 patients recently on sick-leave (<7 weeks) because of depression (F32, F33), anxiety syndrome (F40, F41, F48) and/or stress-related mental disorder (adjustment disorder, exhaustion disorder) (F43) were invited (Fig 1 flowchart). At the intervention PCCs, 308 patients were initially invited, but 40 did not meet inclusion criteria, 83 declined to participate and 185 agreed to participate (89%). At the control PCCs, 191 patients were initially invited, but 13 did not meet inclusion criteria, 22 declined to participate and 156 accepted to participate (88%). In total, 341 patients participated, 54% in intervention and 46% in control group.

Table 2 shows baseline data for intervention and control groups. There were differences concerning age (lower mean age control group) and percentage of men (higher in control group) as well as sedentary level of leisure time physical activity (higher level sedentary in control group).

At the intervention PCCs, early cooperation among care managers, rehabilitation coordinators and the patients' GPs was carried out. Collaboration with team members such as psychotherapists, medical social workers, physiotherapists and occupational therapists was carried out when this was deemed relevant/needed. Out of the185 intervention group patients, 102 (55%) had a person-centred dialogue meeting with the employer within 3 months, in most cases at the PCC. The reason for not having a dialogue meeting was mainly early return to work. There was a significant difference in mean number of days on sick-leave between

For peer review only - http://bmjopen.bmj.com/site/about/guidelines.xhtml

patients who had and patients who did not have a dialogue meeting: mean number of days on sick-leave, 192 vs 117 (patients with vs patients with no dialogue meeting), p<0.001.

Table 2. Baseline data for intervention (n=185) and control group (n=156) patients, total=341 patients, included in the CO-WORK-CARE trial (December 2017 until June 2020).

	Total patient group		Intervention group		Control group	
	**n**	**%**	**n**	**%**	**n**	**%**
**Women**	275	80.6	157	84.9	118	75.6
**Men**	66	19.4	28	15.1	38	24.4
**Occupation Working**	328	96.8	178	96.2	150	97.4
**Studying**	1	0.3	0	0	1	0.6
**In search of work/other**	10	2.9	7	3.8	3	1.9
**Hours of work Full-time**	299	87.9	164	88.6	135	87.1
**Other (25-75%)**	41	12.1	21	11.4	20	12.9
**Marital status Cohabiting**	250	74.0	142	78	108	69.2
**Single**	88	26.0	40	22	48	30.8
**Born Outside Nordic Country**	32	9.4	14	7.6	18	11.5
**Educational level Primary**	20	5.9	13	7	7	4.5
**Secondary**	194	56.9	105	56.8	89	57.0
**University/ college**	127	37.2	67	36.2	60	38.5
**Physical activity leisure time sedentary**	55	16.3	23	12.6	32	20.8
**Smoking Yes+ sometimes**	77	22.6	36	19.5	41	26.5
**Alcohol high (>- 8 p AUDIT)**	27	9.0	16	9.8	11	8.1
**Low socioeconomic status**	178	58.6	96	57.1	82	60.3
**Antidepressant medication**	110	32.3	63	34.1	47	30.1
**Sick-leave last year, self-reported (Yes)**	132	40.9	78	43.8	54	37.2
**Sick- leave diagnosis Depression (F32, F33)**	64	18.8	39	21.1	25	16
**Anxiety syndrome (F40, F41, F48)**	48	14.1	22	11.9	26	16.7
**Stress related mental disorder (F43)**	200	58.7	111	60.0	89	57.1
	**mean**	**SD**	**mean**	**SD**	**mean**	**SD**
**Age**	41.3	11.2	44.2	10.9	37.9	10.7
**MADRS-S**	22.3	8.0	21.7	8.0	22.9	8.1
**GAD-7**	11.6	4.9	11.2	4.7	12.2	5.1
**KEDS**	28.7	8.7	28.2	8.5	29.3	9.1
**EQ5D**	42.8	17.3	43.8	17.1	41.5	17.6
**WAI**	2.56	2.4	2.43	2.4	2.71	2.4

For peer review only - http://bmjopen.bmj.com/site/about/guidelines.xhtml

Vocational outcomes - primary outcome

Analysis of gross and net number of days on sick-leave for intervention and control groups in the Co-Work-Care trial for CMD diagnoses during12 months follow-up showed that there was no significant difference concerning net number of sick-leave days between intervention and control groups (Table 3). The analyses were conducted both for sick-leave due to CMD and for sick-leave due to all diagnoses (mental as well as somatic) during 12 months. All these analyses showed non-significant results for sick-leave days but with shorter duration for control group patients. We also analysed total number of sick-leave days for patients with mild CMD only (n=57), as well as patients with moderate-high CMD symptoms only (n=284), both of which analyses did not show any significant differences concerning sick-leave days (table 3).

Table 3. Gross and net number of days on sick leave for intervention and control groups in the CO-WORK-CARE trial for CMD diagnose (depression and anxiety disorders + adjustment and exhaustion disorders), all diagnoses, and for net number of days for mild and moderate-high CMD diagnoses, respectively. Mixed model analysis adjusted for clustering and age, sex, education, and antidepressants at inclusion Comparison of total number of 12 months sick leave days between intervention and control group.

	**Intervention**		**Control**		
	Mean	SE	Mean	SE	p
GROSS Total number of days all CMD diagnoses	147.30	16.96	125.96	15.16	0.331
NET Total number of days CMD diagnoses	102.48	13.76	96.29	12.38	0.726

For peer review only - http://bmjopen.bmj.com/site/about/guidelines.xhtml

GROSS Total number of days all diagnoses	153.66	16.24	135.82	14.38	0.391
NET Total number of days all diagnoses)	108.98	13.08	104.99	11.68	0.810
NET Total number of days mild CMD diagnoses (n=57)	82.44	20.55	80.97	21.05	0.959
NET Total number of days moderate-high CMD diagnoses (n=284)	115.12	14.12	110.69	12.63	0.803

Supplemental Figure S1 shows survival plot from Cox regression analysis of cumulative return to work (full time or part time sick-leave to no sick-leave), gross number of days on sick-leave 12 months, controlled for age, gender and education. There was no significant difference between intervention and control groups, hazard ratio (HR)=0.881 with 95% CI 0.688 to 1.128.

Secondary outcomes (PROMs)

There were no significant differences in levels at 3, 6 and 12 months between the intervention and control groups concerning depression (MADRS-S), anxiety (GAD-7), or stress (KEDS) symptoms, WAI or QoL (EQ-5D) (table 4, Supplemental Figure S2). For participants with medium/high levels of depression (MADRS-S) and anxiety (GAD-7) symptoms, there were significant differences at 12 months between intervention and control groups, with lower levels in the intervention group. However, there were no differences at all concerning stress symptoms (KEDS) (table 4, Supplemental Figure S3).

For peer review only - http://bmjopen.bmj.com/site/about/guidelines.xhtml

For peer review only

Table 4. Start to 3, 6 and 12 months follow up of differences of mean values between intervention and control group of MADRS-S, GAD-7, KEDS, EQ-5D, and WAI in the CO-WORK-CARE trial, all patients, and patients with low and moderate high levels, respectively. Mixed model analysis adjusted for clustering, age, sex, education, and antidepressants at inclusion.

	Intervention Mean (SE)	Control Mean (SE)	Mean difference (SE)	95% CI	p
MADRS-S at start	22.14 (0.81)	23.04 (0.81)	-0.896 (1.10)	(-3.126, 1.335)	0.421
MADRS-S 3 months	14.01 (0.86)	15.18 (0.88)	-1.17 (1.18)	(-3.549, 1.199)	0.326
MADRS-S 6 months	12.51 (0.86)	13.61 (0.90)	-1.10 (1.19)	(-3.490, 1.292)	0.361
MADRS-S 12 months	11.03 (0.87)	12.79 (0.89)	-1.76 (1.19)	(-4.158, 0.631)	0.146
GAD-7 at start	11.35 (0.44)	11.87 (0.45)	-0.524 (0.597)	(-1.712, 0.664)	0.383
GAD-7 3 months	6.51 (0.47)	7.61 (0.50)	-1,10 (0.66)	(-2.405, 0.200)	0.096
GAD-7 6 months	5.85 (0.43)	6.37 (0.46)	-0.52 (0.59)	(-1.689, 0.658)	0.385
GAD-7 12 months	5.49 (0.46)	6.62 (0.49)	-1.13 (0.64)	(-2.402, 0.140)	0.081
KEDS at start	28.11 (1.09)	28.93 (1.06)	-0.823 (1.47)	(-3.814, 2.167)	0.579
KEDS 3 months	19.43 (1.15)	20.52 (1.16)	-1.082 (1.59)	(-4.275, 2.110)	0.498
KEDS 6 months	17.54 (1.17)	18.22 (1.18)	-0.679 (1.62)	(-3.926, 2.568)	0.676
KEDS 12 months	15.69 (1.19)	16.78 (1.20)	-1.095 (1.65)	(-4.399, 2.209)	0.509
EQ-5D at start	43.83 (1.89)	40.42 (1.86)	3.41 (2.54)	(-1.748, 8.569)	0.188
EQ-5D 3 months	63.02 (2.10)	60.29 (2.15)	2.73 (2.91)	(-3.086, 8.552)	0.351
EQ-5D 6 months	71.13 (2.08)	66.23 (2.15)	4.90 (2.88)	(-0.866, 10.669)	0.094

For peer review only - http://bmjopen.bmj.com/site/about/guidelines.xhtml

EQ-5D 12 months	72.34 (2.08)	67.92 (2.12)	4.43 (2.87)	(-1.309, 10.168)	0.128
WAI at start	2.53 (0.29)	2.65 (0.28)	-0.12 (0.39)	(-0.901, 0.663)	0.761
WAI 3 months	5.48 (0.32)	5.42 (0.33)	0.06 (0.44)	(-0.833, 0.944)	0.901
WAI 6 months	6.94 (0.31)	6.45 (0.31)	0.49 (0.43)	(-0.360, 1.340)	0.254
WAI 12 months	7.55 (0.31)	7.08 (0.31)	0.47 (0.43)	(-0.376, 1.323)	0.269
MADRS-S>19 at start	26.97 (0.63)	28.19 (0.62)	-1.21 (0.82)	(-2.880, 0.457)	0.150
MADRS-S>19, 3 months	16.16 (0.91)	18.44 (0.96)	-2.28 (1.28)	(-4.822, 0.255)	0.078
MADRS-S>19, 6 months	14.04 (0.96)	16.35 (1.05)	-2.31 (1.37)	(-5.016, 0.396)	0.094
MADRS-S>19, 12 months	11.71 (0.92)	15.30 (1.30)	-3.59 (1.31)	(-6.189, -0.993)	**0.007**
GAD-7>9 at start	14.03 (0.37)	15.13 (0.37)	-1.10 (0.597)	(-2.089, -0.112)	**0.030**
GAD-7>9, 3 months	7.32 (0.58)	8.73 (0.63)	-1,42 (0.83)	(-3.069, 0.230)	0.091
GAD-7>9, 6 months	6.39 (0.53)	7.80 (0.58)	-1.41 (0.76)	(-2.908, 0.094)	0.066
GAD-7>9, 12 months	5.54 (0.58)	7.89 (0.63)	-2.34 (0.83)	(-3.985, -0.696)	**0.006**
KEDS>29 at start	34.88 (0.73)	36.34 (0.70)	-1.46 (0.93)	(-3.365, 0.445)	0.128
KEDS>29, 3 months	23.46 (1.14)	25.00 (1.19)	-1.537 (1.60)	(-4.704, 1.631)	0.339
KEDS>29, 6 months	21.25 (1.23)	22.27 (1.28)	-1.02 (1.73)	(-4.436, 2.404)	0.558
KEDS>29, 12 months	18.56 (1.23)	20.55 (1.30)	-1.99 (1.73)	(-5.428, 1.447)	0.254

Bold figures, p<0.05

For peer review only - http://bmjopen.bmj.com/site/about/guidelines.xhtml

Table 5 shows participants on sick-leave at baseline, 3, 6 and 12 months. At 3 months, there was a significant difference between intervention and control groups with lower percentage of patients on sick-leave in the control group, but no significant differences at 6 and 12 months. Table 5 also shows comparisons between participants in intervention and control groups with mild and moderate-high levels, respectively, concerning depression symptoms, anxiety symptoms and stress symptoms at respective point in time.

Percentage of patients on antidepressants and patients receiving psychotherapy contact did not show any significant differences at 3, 6 or 12 months (Table 5).

Subgroup analyses

Analysis including only patients that participated in a person-centred dialogue meeting (n=102) compared to control group did not reveal different results from comparison of the total intervention and control groups.

Harmful events

No harmful events, suicide attempts or psychiatric hospital care were registered for patients included in the CO-WORK-CARE trial during the 12 months observation time.

For peer review only - http://bmjopen.bmj.com/site/about/guidelines.xhtml

Table 5. Number and percentage of participants in intervention and control groups with mild and moderate-high levels, respectively, of depression symptoms, anxiety symptoms and stress symptoms according to MADRS-S, GAD-7 and KEDS. Levels at baseline, 3, 6, and 12 months, as well as number and percentage of participants on sick-leave at respective point in time, on antidepressants and receiving psychotherapy contact. Bold figures indicate significant difference between intervention and control groups.

	Baseline		3 months		6 months		12 months	
	Intervention	Control	Intervention	Control	Intervention	Control	Intervention	Control
	n (%)	n (%)	n (%)	n (%)	n (%)	n (%)	n (%)	n (%)
On sick-leave	184 (99.5)	156 (100)	**114 (62.0)**	**80 (51.3)**	66 (35.9)	46 (29.5)	28 (15.2)	24 (15.4)
MADRS-S>12	160 (87.4)	143 (91.7)	**72 (46.5)**	**75 (58.6)**	62 (42.5)	54 (48.6)	49 (34.3)	49 (43.4)
MADRS-S>19	116 (63.4)	100 (64.1)	39 (25.2)	35 (27.3)	27 (18.5)	25 (22.5)	**15 (10.5)**	**23 (20.4)**
GAD>4	165 (92.2)	140 (92.1)	91 (59.9)	90 (70.9)	87 (59.6)’	71 (63.4)	73 (51.8)	61 (53.5)
GAD>9	120 (67.0)	99 (65.1)	33 (21.7)	40 (31.5)	**21 (14.4)**	**28 (25.0)**	**19 (13.5)**	**31 (27.2)**
KEDS>18	162 (88.5)	132 (86.5)	82 (52.2)	77 (60.2)	68 (46.6)	52 (46.0)	52 (36.4)	44 (38.9)
KEDS>29	94 (51.4)	83 (54.6)	26 (16.6)	27 (21.1)	17 (11.6)	18 (15.9)	11 (7.7)	12 (10.6)
Anti-depressant use	63 (34.1)	47 (30.1)	60 (38.2)	50 (39.1)	54 (36.5)	41 (36.0)	49 (34.0)	40 (35.1)
Psychotherapy contact	-	-	70 (44.6)	71(55.0)	58 (39.2)	39 (34.2)	28 (19.4)	15 (13.2)

For peer review only - http://bmjopen.bmj.com/site/about/guidelines.xhtml

Discussion

Main findings

The main finding was that there were no differences in sick-leave days between intervention and control groups. A structured early cooperation among care manager, GP and rehabilitation coordinator at the PCC combined with a person-centred dialogue meeting between the sick-listed person and employer, with the rehabilitation coordinator as a moderator, did not lead to shortened sick-leave time in comparison with usual care manager contact only. Patients with CMD diagnoses – both when sick-leave days were studied for CMD diagnoses only as well as total sick-leave for all diagnoses – showed no significant differences concerning sick-leave days.

However, for patients with moderate to high symptom levels of depression and anxiety there were also significant differences in levels of CMD depression and/or anxiety symptoms at 12 months between intervention and control groups, but no difference at all concerning stress symptoms. This indicates that a closer cooperation and a planned person-centred dialogue meeting could be beneficial for the mental well-being of persons with moderate-high symptom levels of depression/anxiety, as indicated by significantly lower levels of depression and anxiety symptoms after 12 months. This is an important finding, as individuals with severe symptoms of depression and anxiety may be the most difficult to help and have been shown to have the longest sick-leave period among patients with CMD (6, 7).

Strengths and weaknesses

The strengths of the present study are several. The trial was accomplished entirely in primary care. All participating PCC personnel as well as research personnel had very good contextual knowledge, and the intervention was adapted to a level possible to conduct in a Swedish

For peer review only - http://bmjopen.bmj.com/site/about/guidelines.xhtml

primary care context with low level of resources for direct workplace intervention and cooperation. The intervention model was person-centred and emphasised the individual's perceptions of workplace conditions facilitating own RTW. We succeeded in achieving the planned sample size of patients, despite the occurrence of the Covid19 epidemic during the last 3 months before the 12 months follow-up was finalised for the latest included participants.

Among limitations, the risk of identification and recruitment bias was a limitation since providers were aware of being in the intervention or control arm. More patients participated in the intervention arm than in the control arm, and there were some differences between the arms in the baseline characteristics of recruited patients. The impact that Covid19 could have had on the last months of follow-up for RTW and 12 months sick-leave duration should also be mentioned. However, this effect should have been comparable in the control group. Another limitation was that only around 55% of patients in the intervention group took part in the person-centred dialogue meeting. A major reason for this was that the dialogue meeting was not deemed relevant or needed for the patients with early return to work in the fast rehabilitation process, and this was mostly due to the patient's own preference. Another limitation was that multiplicity was not accounted for in the analyses of secondary outcomes.

The complexity of the intervention based on the individual patient's preferences, needs and values, a structured cooperation among GP, care manager and rehabilitation coordinator and a person-centred dialogue meeting with employer could be called into question concerning possible biasing factors. However, primary care is a complex clinical context where the handling of care problems is guided by the individual patient's needs as the starting point, and the pragmatic design of the trial compensates at least partly for multiple biasing factors.

For peer review only - http://bmjopen.bmj.com/site/about/guidelines.xhtml

A further limitation was that the trial was only possible to carry out in a primary health care system where a collaborative care model and team-based care were implemented. However, the collaborative care model with a care manager function has also been implemented in several European countries such as the UK, Netherlands, and Italy, in addition to Canada and the US (26). The care manager organisation implemented in the Region Västra Götaland has been judged to represent a collaborative care programme for depression and anxiety disorder that makes possible personalised care planning and shared decision making (27).

Other studies

Several complex interventions, of which some mainly in the primary care context, aiming at increasing RTW and reducing sick-leave time have been performed. Bakker et al already in 2007 published a cluster-randomised controlled educational trial consisting of a minimal intervention for patients with stress-related mental disorders on sick-leave in primary care (MISS) that aimed at reducing sick-leave and preventing chronicity of symptoms (7). No superior effect of MISS was found concerning duration of sick-leave or severity of symptoms compared to care as usual (GP contact at PCCs) after 12 months (7). The latest published RCT on interventions aimed at integrating vocational rehabilitation and mental healthcare in people with depression and anxiety, where an integrated intervention both in healthcare and at the workplace was compared to improved mental care and care as usual, showed no increased RTW, although a higher proportion in work at 12 months was shown for the integrated intervention (28). The authors concluded that the goal of a very fast return to work is not necessarily beneficial, and that integration gave slightly better outcomes, but the clinical significance, i.e. health advantage, was questionable (28).

Interpretation of findings

For peer review only - http://bmjopen.bmj.com/site/about/guidelines.xhtml

The results of the trial indicate that providing more intense contacts with care and the workplace for patients with CMD with a symptom burden and reduction in work ability high enough to cause need of sick-leave did not lead to earlier return to full time work participation compared to the usual level of support to the patient provided by the care manager in a collaborative care organisation together with access to psychologic and other therapeutic competences at the PCC. It seems that it is not possible to hurry up the processes that these patients need. Building a person-centred care process based on accessibility, continuity and close contact with a care manager makes it possible to adjust the care and timing to the individual patent's needs. The level of support and care that the care manager provides for the patient during the initial 3 months of the CMD illness seems 'good enough'. Extra care efforts on top of this, as provided in this intervention, do not seem to improve the patient's state. It is possible that a more thorough dialogue with greater competency is needed or, alternatively, repeated meetings to achieve a faster RTW. The person-centred dialogue meeting may achieve a better understanding on the part of the employer for the worker's health situation but a change in the work situation might not be possible anyway.

RCT trials have shown effectiveness of collaborative care and care management in patients with depression, and the CO-WORK-CARE trial further develops the collaborative care/care manager intervention with structured early cooperation between the PCC competencies, including the GP and rehabilitation personnel, and patients on sick-leave because of depression, anxiety and stress-related mental disorder. Care management was the basis of care in both arms, while the early and structured cooperation among the care manager, rehabilitation coordinator and the GP coupled with the person-centred dialogue meeting with employer the was the added value of the intervention. The effects of the structured cooperation were only discernible on the patient level. Early and structured cooperation coupled with a dialogue meeting can thus be said to be more effective in the long run for

For peer review only - http://bmjopen.bmj.com/site/about/guidelines.xhtml

patients concerning their moderate-serious depression and anxiety symptoms than usual care manager contact, but not at all more effective concerning sick-leave duration. Interview studies of patients' and personnel's perceptions of working according to the CO-WORK-CARE model reveal that patients perceived the person-centred dialogue meeting with the employer as positive (29), and care managers and rehabilitation coordinators appreciated the cooperation (30). The doctors in particular regarded the CO-WORK-CARE model as one that facilitated their often complex and frustrating work situation, and they perceived that they gained better control and support for the care of the patient through the close collaboration (31).

Implications

A good enough way to help the patient back to work in a salutogenic way seems primarily to be to provide accessibility, continuity, close contact and emphasis on person-centred care, to follow the patient continuously and to await start of workplace contact until the patient is ready for this.

Acknowledgements

We want to thank all primary care centres in Västra Götaland who have been engaged in the CO-WORK-CARE study for their engagement and participation.

Data sharing statement

Data are not publicly available due to Swedish law but are available from the authors on reasonable request. The data are stored at the Gothenburg University, Arvid Wallgrens backe 7, 40530 Göteborg, Sweden. Contact details: Cecilia Björkelund Orcid iD:

For peer review only - http://bmjopen.bmj.com/site/about/guidelines.xhtml

For peer review only

0000- 0003- 4083- 7342.

Competing interests

All authors have completed the Unified Competing Interest form (available on request from the corresponding author) and declare: no support from any organisation for the submitted work ;no financial relationships with any organisations that might have an interest in the submitted work in the previous three years; no other relationships or activities that could appear to have influenced the submitted work.

Contributors

CB was the principal investigator and guarantor of the project. CB, E-LP, IS, DH and GH participated in the design of the work. CB, E-LP, IS, DH and NA handled the data and had the main responsibility for conducting the analyses, GH, SN, IJ contributed to conduction of additional analyses. CB, AS, GH, E-LP, IS, DH, IJ, NA, SN, KT, ML, CW and LW were responsible for the writing of the paper. All authors contributed to the interpretation of data and critical text revision and read and approved the final version of this manuscript. The corresponding author attests that all listed authors meet authorship criteria and that no others meeting the criteria have been omitted.

Transparency declaration

The manuscript's guarantor affirms that the manuscript is an honest, accurate, and transparent account of the study being reported; that no important aspects of the study have been omitted; and that any discrepancies from the study as planned and registered have been explained.

Copyright

The Corresponding Author has the right to grant on behalf of all authors and does grant on behalf of all authors, an exclusive licence and/or a non-exclusive licence for contributions from authors who are: i) UK Crown employees; ii) where BMJ has agreed a CC-BY licence shall apply, and/or iii) in accordance with the terms applicable for US Federal Government officers or employees acting as part of their official duties; on a worldwide, perpetual, irrevocable, royalty-free basis to BMJ Publishing Group Ltd ("BMJ") its licensees.

The Submitting Author accepts and understands that any supply made under these terms is made by

For peer review only - http://bmjopen.bmj.com/site/about/guidelines.xhtml

BMJ to the Submitting Author unless you are acting as an employee on behalf of your employer or a postgraduate student of an affiliated institution which is paying any applicable article publishing charge ("APC") for Open Access articles. Where the Submitting Author wishes to make the Work available on an Open Access basis (and intends to pay the relevant APC), the terms of reuse of such Open Access shall be governed by a Creative Commons licence – details of these licences and which licence will apply to this Work are set out in our licence referred to above.

Ethical approval

This study conforms to the principles outlined in the Declaration of Helsinki. All participants were handed written and oral information about the purpose of the study, confidentiality and voluntary nature of participation as well as right to withdraw from the study at any time. Informed consent was obtained from all participants.

Written permission for conduct of study was obtained from the head of the regional primary healthcare authority and all participating primary care centres. Ethical approval was obtained from the Regional Ethical Review Board Gothenburg, Sweden Dnr: 459-17; T745-18.

Funding

This work was supported by Grants from Forte Dnr 2016-07412, Dnr 2018-01266 and grants from the Swedish state under the agreement between the Swedish government and the county councils, the ALF agreement (ALFGBG-722441, ALFGBG-965520**).** The funding organisations have no role in the planning, execution or analyses of the trial.

ORCID iDs

Cecilia Björkelund http://orcid.org/0000-0003-4083-7342

Irene Svenningsson http://orcid.org/0000-0002-7421-8171

Dominique Hange http://orcid.org/0000-0003-1114-444

For peer review only - http://bmjopen.bmj.com/site/about/guidelines.xhtml

References

1. Försäkringskassan. Sjukfrånvaro i psykiatriska diagnoser. En registerstudie av Sveriges arbetande befolkning i åldern 20–69 år. Socialförsäkringsrapport 2020:8. (Swedish Social Insurance Agency. Mental disorder sick leave – A register study of the Swedish working population in ages 20 to 69 years. Social Insurance Report 2020.8. English summary) Available at https://www.forsakringskassan.se/download/18.7fc616c01814e179a9f329/1656660446139/sjukfranvaro-i-psykiatriska-diagnoser-socialforsakringsrapport-2020-8.pdf. (Accessed 27th March 2023)
2. WHO methods and data sources for global burden of disease estimates 2000-2019. WHO Geneva December 2020. https://cdn.who.int/media/docs/default-source/gho-documents/global-health-estimates/ghe2019_daly-methods.pdf. (Accessed 27th March 2023)
3. Bakker IM, Terluin B, van Marwijk HWJ, van der Windt D. et al. A Cluster-Randomised Trial Evaluating an Intervention for Patients with Stress-Related Mental Disorders and Sick Leave in Primary Care. PLOS Clin Trial 2007;2(6):e26.
4. McCrone P, Dhanasiri S, Patel A, Knapp M, Lawton -Smith S. Paying the price. The cost of mental health in England to 2026. Kings Fund 2008. Available at : https://www.kingsfund.org.uk/sites/default/files/Paying-the-Price-the-cost-of-mental-health-care-England-2026-McCrone-Dhanasiri-Patel-Knapp-Lawton-Smith-Kings-Fund-May-2008_0.pdf. (Accessed 27th March 2023)
5. Hassard J, Teoh R H, Visockaite G, Dewe P, Cox T. The cost of work-related stress to society: A systematic review. Journal of Occupational Health Psychology. 2018; 23(1): 1–17.
6. Vingård E. Psykisk ohälsa, arbetsliv och sjukfrånvaro. (Mental Health, working life and sick leave. In Swedish). Forte. ISBN: 978-91-88561-31-2 https://forte.se/app/uploads/2015/04/psykisk-ohalsa-arbetsliv.pdf. (Accessed 27th March 2023)
7. Nystuen P, Hagen KB, Herrin J. Mental health problems as a cause of long term sick leave in the Norwegian workforce. SJPH 2001;29:175-182.
8. Salomonsson S, Santoft F, Lindsäter E, et al. Cognitive-behavioural therapy and return-to-work intervention for patients on sick leave due to common mental disorders: a randomised controlled trial. Occup Environ Med. 2017 Dec;74(12):905-912.
9. Lejtzen N, Sundquist J, Sundquist K, Li X. Depression and anxiety in Swedish primary health care: prevalence, incidence, and risk factors. Eur Arch Psychiatry Clin Neurosci. 2014;264(3):235-45
10. Björkelund C, Svenningsson I, Hange D, et al. Clinical effectiveness of care managers in collaborative care for patients with depression in Swedish primary health care: a pragmatic cluster randomized controlled trial. BMC Family Practice. 2018;19:28.
11. Huffman JC, Niazi SK, Rundell JR, Sharpe M, Katon WJ. Essential articles on collaborative care models for the treatment of psychiatric disorders in medical settings: a publication by the academy of psychosomatic medicine research and evidence-based practice committee. Psychosomatics. 2014;55(2):109-22
12. Nieuwenhuijsen K, Verbeek JH, Neumeyer-Gromen A, Verhoeven AC, Bültmann U, Faber B. Interventions to improve return to work in depressed people. Cochrane Database of Systematic Reviews 2020, Issue 10. Art. No.: CD006237.
13. Arends I, Bruinvels DJ, Redebergen DS, et al. Interventions to facilitate return to work in adults with adjustment disorders. Cochrane Database of Systematic Reviews 2012, Issue 12. Art. No CD006389. https://doi.org/10.1002/14651858.CD006389.pub2 (27th March 2023).
14. Petersson EL, Hange D, Udo C, Björkelund C, Svenningsson I. Long-term effect of a care manager on work ability for patients with depression - the PRIM-CARE RCT. Work. 2022;72(2):601-609.
15. Udo C, Svenningsson I, Björkelund C, Hange D, Jerlock M, Petersson E-L. An interview study of the care manager function – Opening the door to continuity of care for patients with depression in primary care. Nursing Open. 2019;6(3):974-82.

For peer review only - http://bmjopen.bmj.com/site/about/guidelines.xhtml

16. National Board of Health and Welfare. National guidelines for depression and anxiety syndromes. (In Swedish). https://www.socialstyrelsen.se/globalassets/sharepoint-dokument/artikelkatalog/nationella-riktlinjer/2021-4-7339.pdf (Accessed 27th March 202327th).
17. Spitzer RL, Williams JB, Kroenke K, et al. Utility of a new procedure for diagnosing mental disorders in primary care. The PRIME-MD 1000 study. JAMA 1994; 272:1749-1756.)
18. Besèr A, Sorjonen K, Wahlberg K, Peterson U, Nygren Å, Åsberg M. Construction and evaluation of a self rating scale for stress-induced Exhaustion Disorder, the Karolinska Exhaustion Disorder Scale. Scandinavian Journal of Psychology. 2013;55: 72–82.)
19. Wiegner L, Hange D, Björkelund C, Ahlborg Jr G. Prevalence of perceived stress and associations to symptoms of exhaustion, depression and anxiety in a working age population seeking primary care – an observational study. BMC Family Practice 2015 16:38 DOI 10.1186/s12875-015-0252-7.
20. Karlson B, Östberg K. ADA ArbetsplatsDialog för Arbetsåtergång. (Place of work dialogue for return to work. In Swedish) Användarmanual v.1.3. Lund: Avd för arbets- och miljömedicin. Lunds universitet; 2014.
21. Fors A, Ekman I, Taft C, et al. Person-centred care after acute coronary syndrome, from hospital to primary care - A randomised controlled trial. Int J Cardiol. 2015;187:693-9).
22. Montgomery SA, Asberg M. A new depression scale designed to be sensitive to change. The British Journal of Psychiatry. 1979;134:382-9.
23. Spitzer RL, Kroenke K, Williams JB, et al. A brief measure for assessing generalized anxiety disorder: the GAD-7. Arch Intern Med 2006;166(10):1092-7.
24. EuroQol Group, EuroQol - a new facility for the mesurement of health-related quality of life. The EuroQol Group, Health Policy, 16 (19990) 199-208.
25. Ilmarinen J. The Work Ability Index (WAI). Occup Med. 2007;57(2):160.
26. Sighinolfi C, Nespeca C, Menchetti M, Levantesi P, Belvederi Murri M, Berardi D. Collaborative care for depression in European countries: a systematic review and meta-analysis. J Psychosom Res. 2014 Oct;77(4):247-63.
27. Menear M, Girard A, Dugas M, Gervais M, Gilbert M, Gagnon M-P. Personalized care planning and shared decision making in collaborative care programs for depression and anxiety disorders: A systematic review. PLoS One. 2022 Jun 10;17(6): e0268649
28. Hoff A, Poulsen RM, Fisker J, et al. Integrating vocational rehabilitation and mental healthcare to improve the return-to-work process for people on sick leave with depression or anxiety: results from a three-arm, parallel randomised trial. Occup Environ Med. 2022 Feb;79(2):134-142.
29. Saxvik A, Törnbom K, Petersson EL, et al. Experiences of patients with common mental disorders concerning team-based primary care and a person-centered dialogue meeting: An intervention to promote return to work. PLoS One. 2022 Jul 8;17(7):e0271180.
30. Petersson EL, Törnbom K, Hange D, et al. The experiences of care managers and rehabilitation coordinators of a primary care intervention to promote return to work for patients with common mental disorders: a qualitative study. BMC Fam Pract. 2020 Dec 18;21(1):272.
31. Saxvik A, Svenningsson I, Tornbom K, et al. "Gaining control through close collaboration" - GPs' experiences of a Collaborative Care Model for patients with Common Mental Disorders who need sick leave certification. BJGP Open. 2022:BJGPO.2022.0042.

For peer review only - http://bmjopen.bmj.com/site/about/guidelines.xhtml

Figure legends

Figure1. Flow chart for the CO-WORK-CARE trial; inclusion of primary care centres (upper part) and inclusion of patients, subdivided by intervention and control identification of primary care centres (lower part).

Supplemental Figure S1. Survival pattern of cumulative return to work (full or part time sick-leave to no sick-leave), gross number of days on sick-leave 12 months. Cox regression analysis controlled for age, gender and education. No significant difference between intervention (blue) and control (green) group RTW (hazard ratio=0.881, 95% CI 0.688 to 1.128).

Supplemental Figure S2. Course of MADRS-S (a), GAD-7(b), KEDS (c), WAI (d), and EQ-5D (e) from baseline to 12 months follow up (adjusted means). Mixed model analysis, adjusted for clustering, age, sex, education, antidepressants at inclusion.

Supplemental Figure S3. Course of MADRS-S, GAD-7, KEDS for patients with moderate/ high levels (n= 284) from baseline to 12 months follow up (adjusted means). Mixed model analysis, adjusted for clustering, age, sex, education, and antidepressants at inclusion. a) All patients with MADRS-S $\geq$ 20: significant difference at 12 months; b) All patients with GAD$\geq$10: significant difference at 12 months; c) All patients with KEDS$\geq$ 30: no significant differences.

For peer review only - http://bmjopen.bmj.com/site/about/guidelines.xhtml

Fig 1

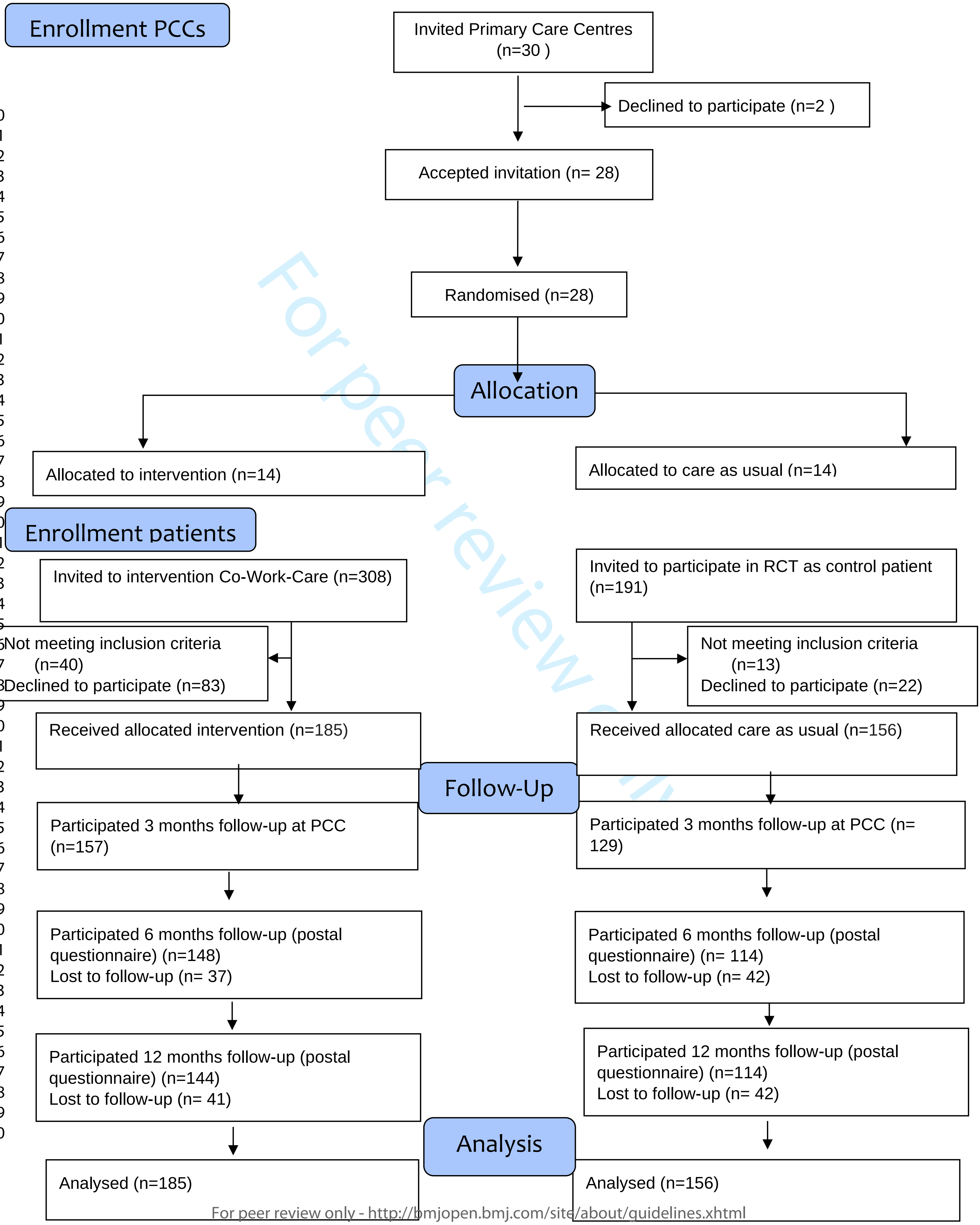

For peer review only - http://bmjopen.bmj.com/site/about/guidelines.xhtml

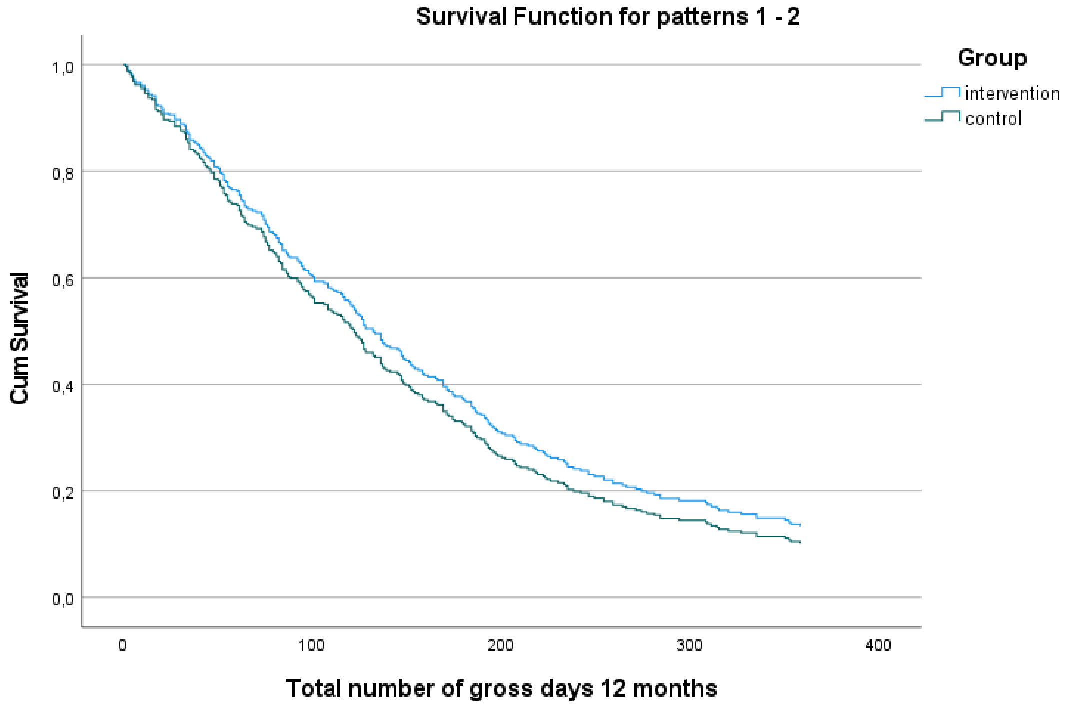

For peer review only

a)

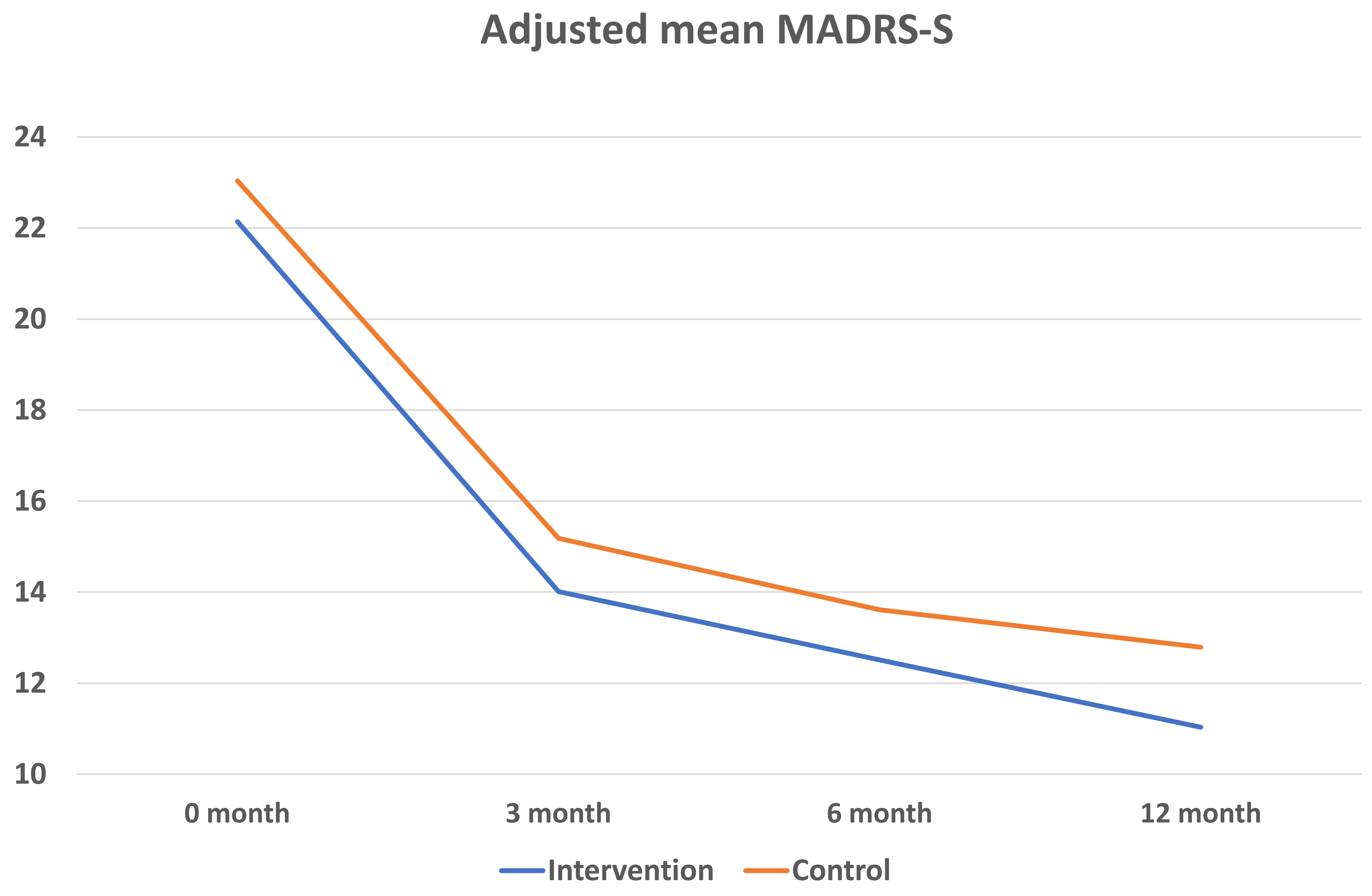
Adjusted mean MADRS-S
24
22
20
18
16
14
12
10
0 month
3 month
6 month
12 month
Intervention
Control

b)

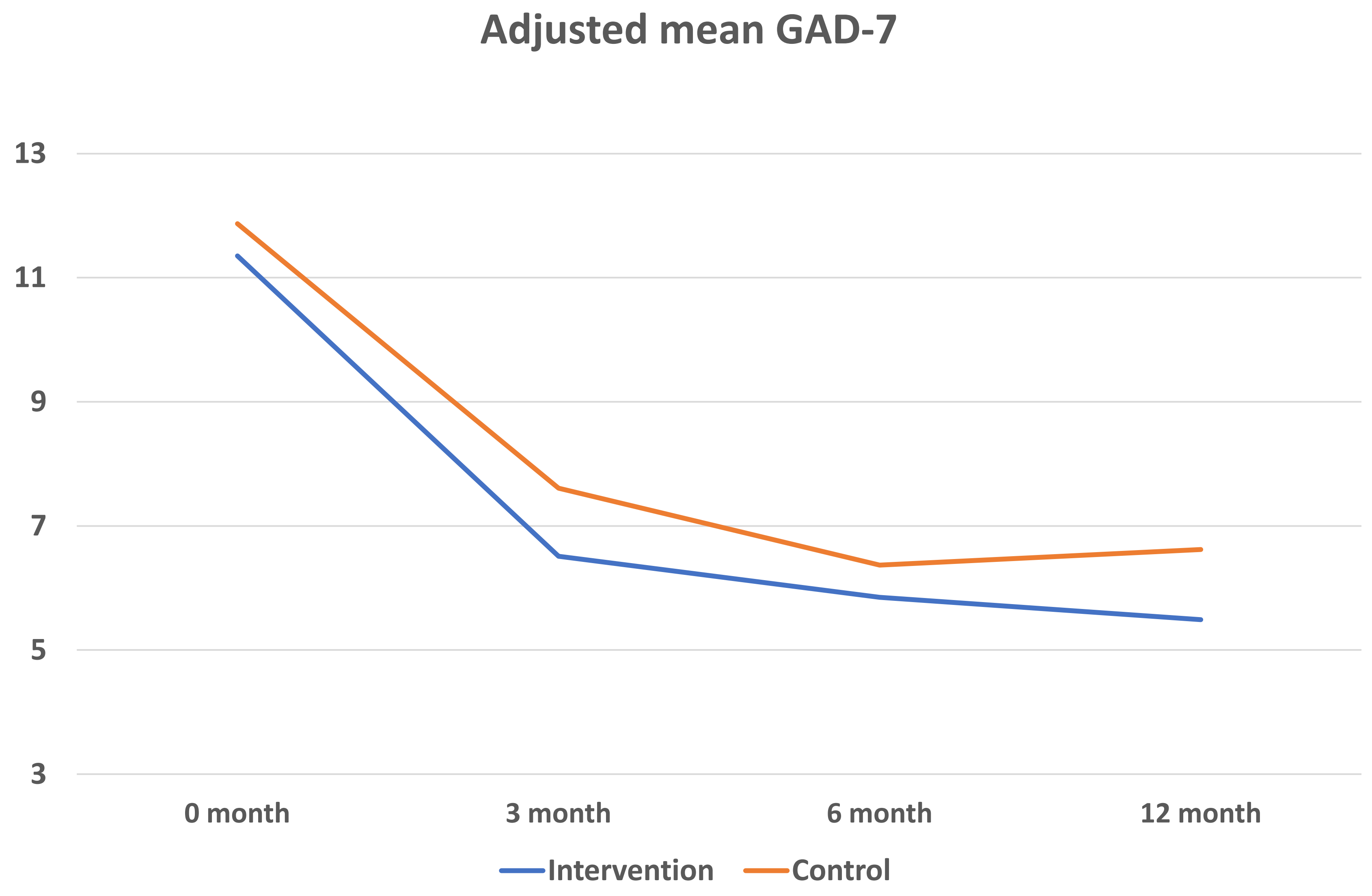
Adjusted mean GAD-7
13
11
9
7
5
3
0 month
3 month
6 month
12 month
Intervention
Control

c)

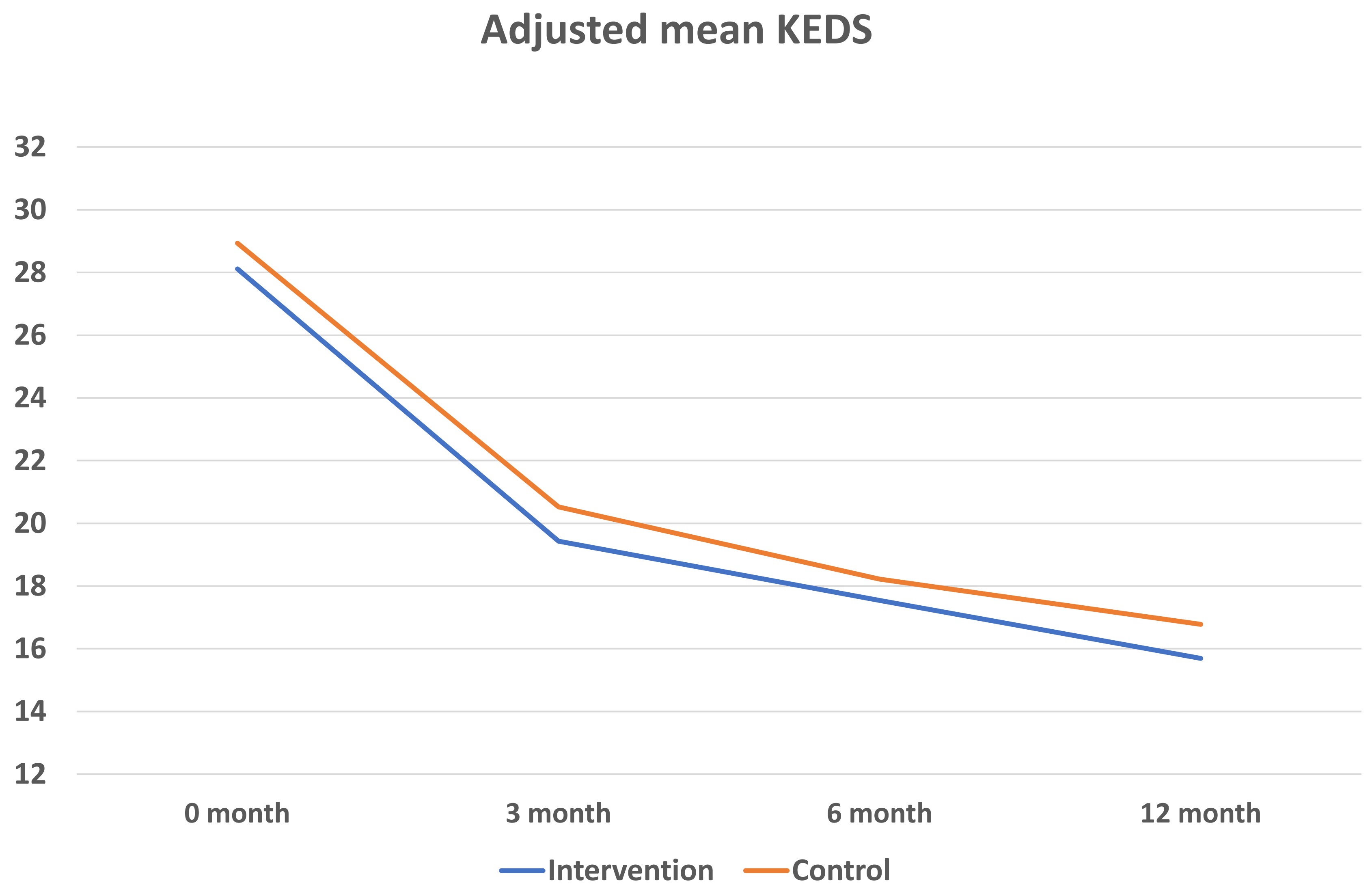
Adjusted mean KEDS
32
30
28
26
24
22
20
18
16
14
12
0 month
3 month
6 month
12 month
Intervention
Control

d)

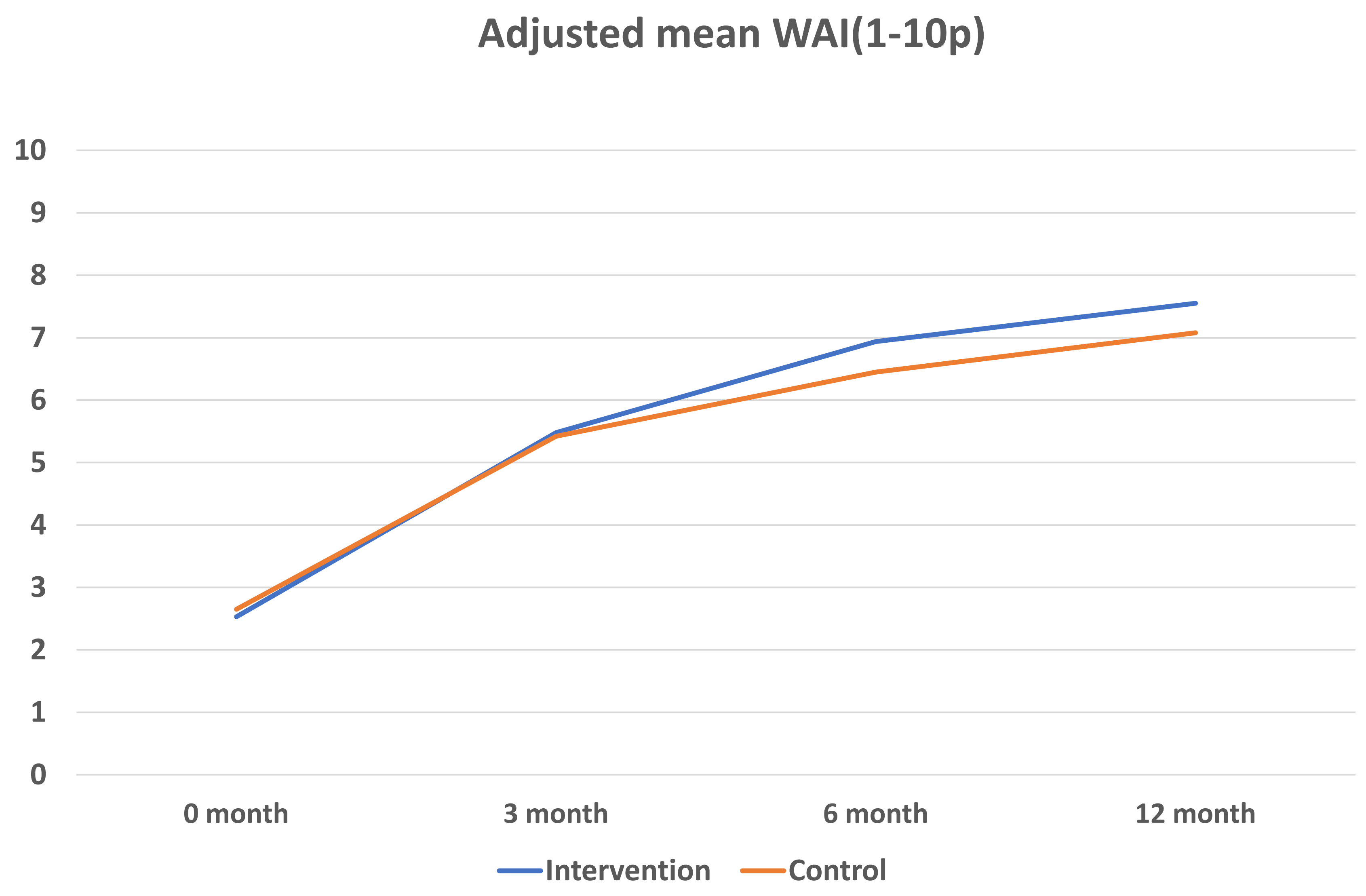
Adjusted mean WAI(1-10p)
10
9
8
7
6
5
4
3
2
1
0
0 month
3 month
6 month
12 month
Intervention
Control

e)

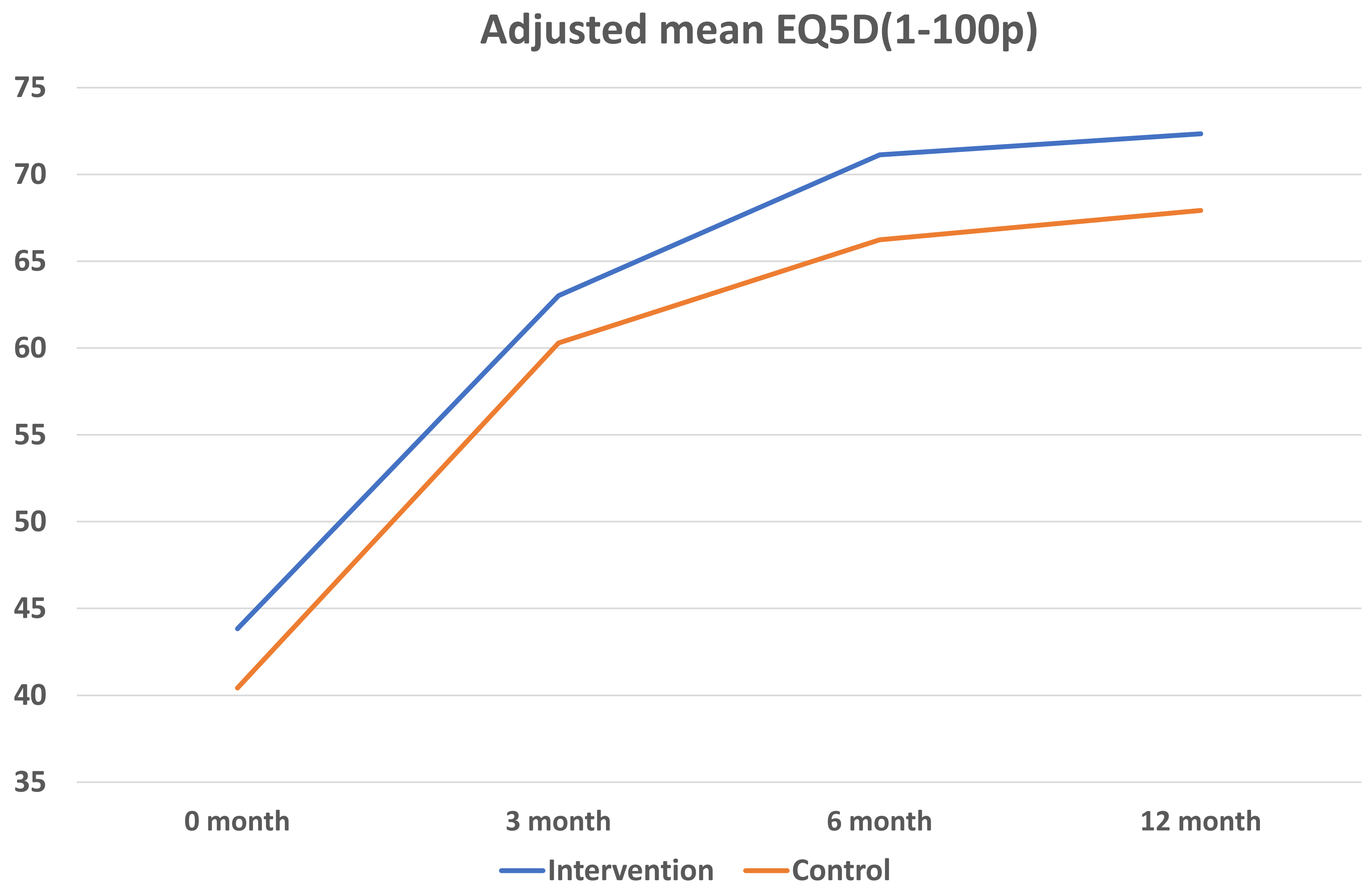
Adjusted mean EQ5D(1-100p)
75
70
65
60
55
50
45
40
35
0 month
3 month
6 month
12 month
Intervention
Control

a)

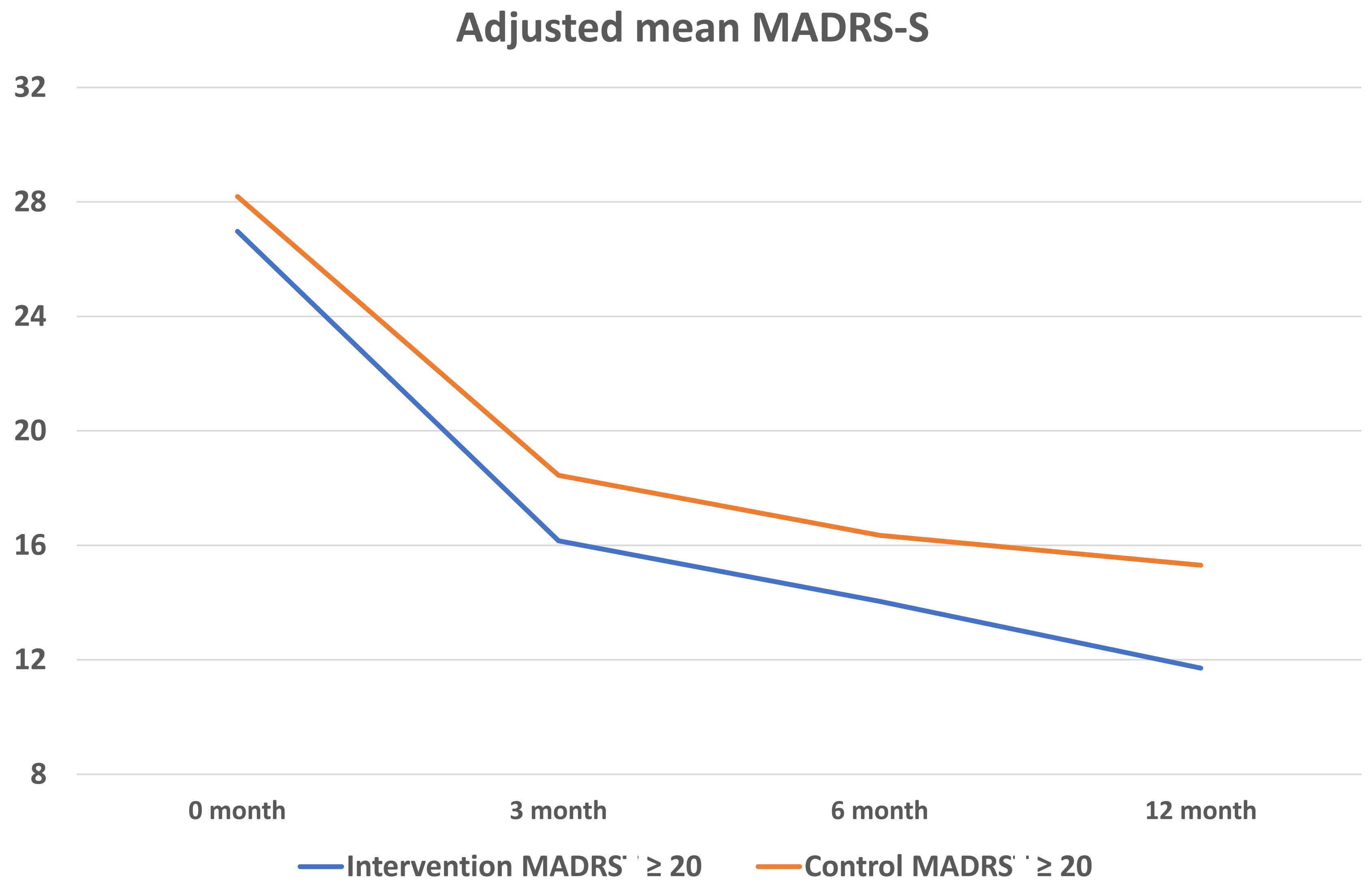
Adjusted mean MADRS-S
32
28
24
20
16
12
8
0 month
3 month
6 month
12 month
Intervention MADRS[illegible] ≥ 20
Control MADRS[illegible] ≥ 20

b)

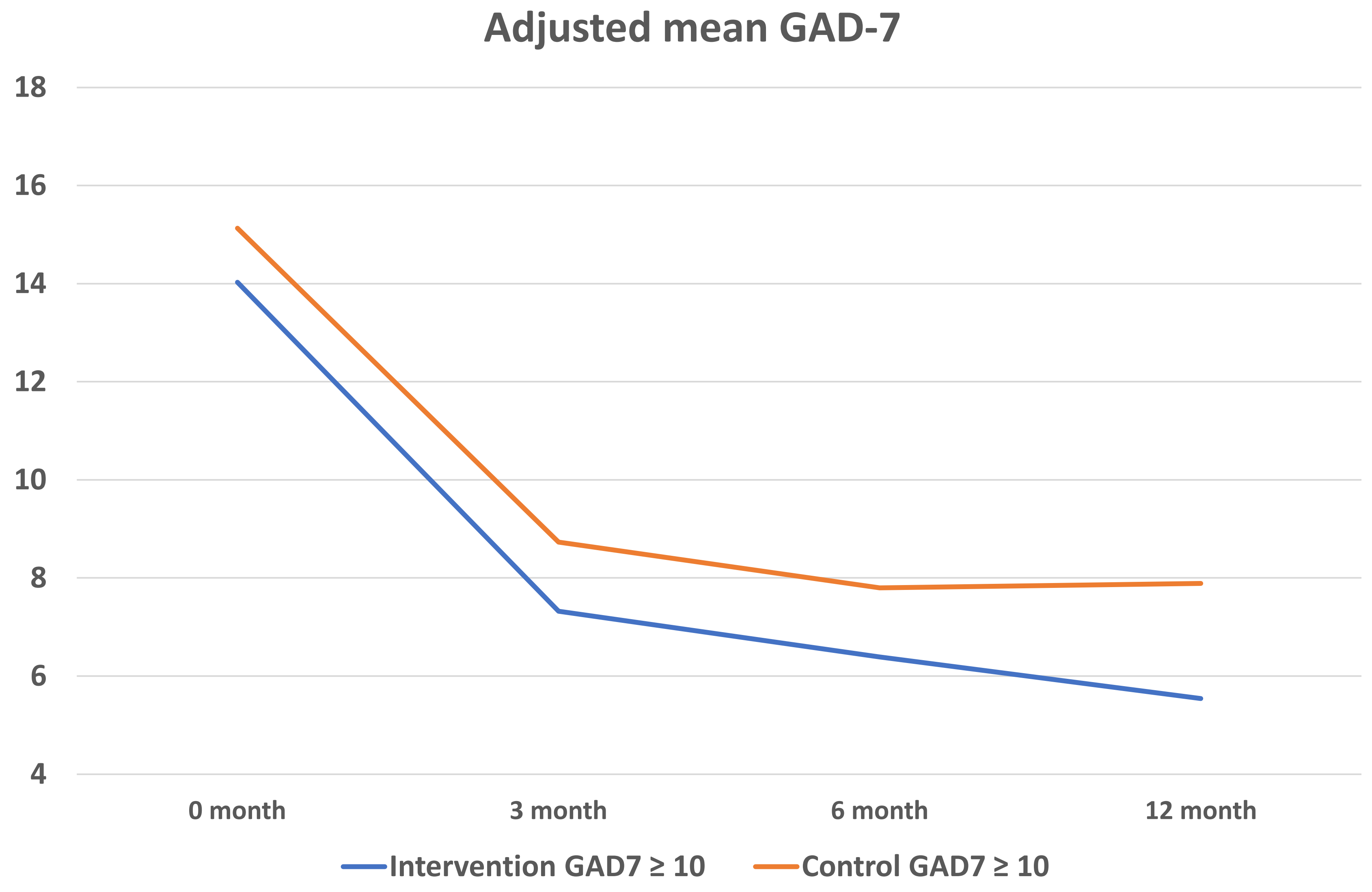
Adjusted mean GAD-7
18
16
14
12
10
8
6
4
0 month
3 month
6 month
12 month
Intervention GAD7 ≥ 10
Control GAD7 ≥ 10

c)

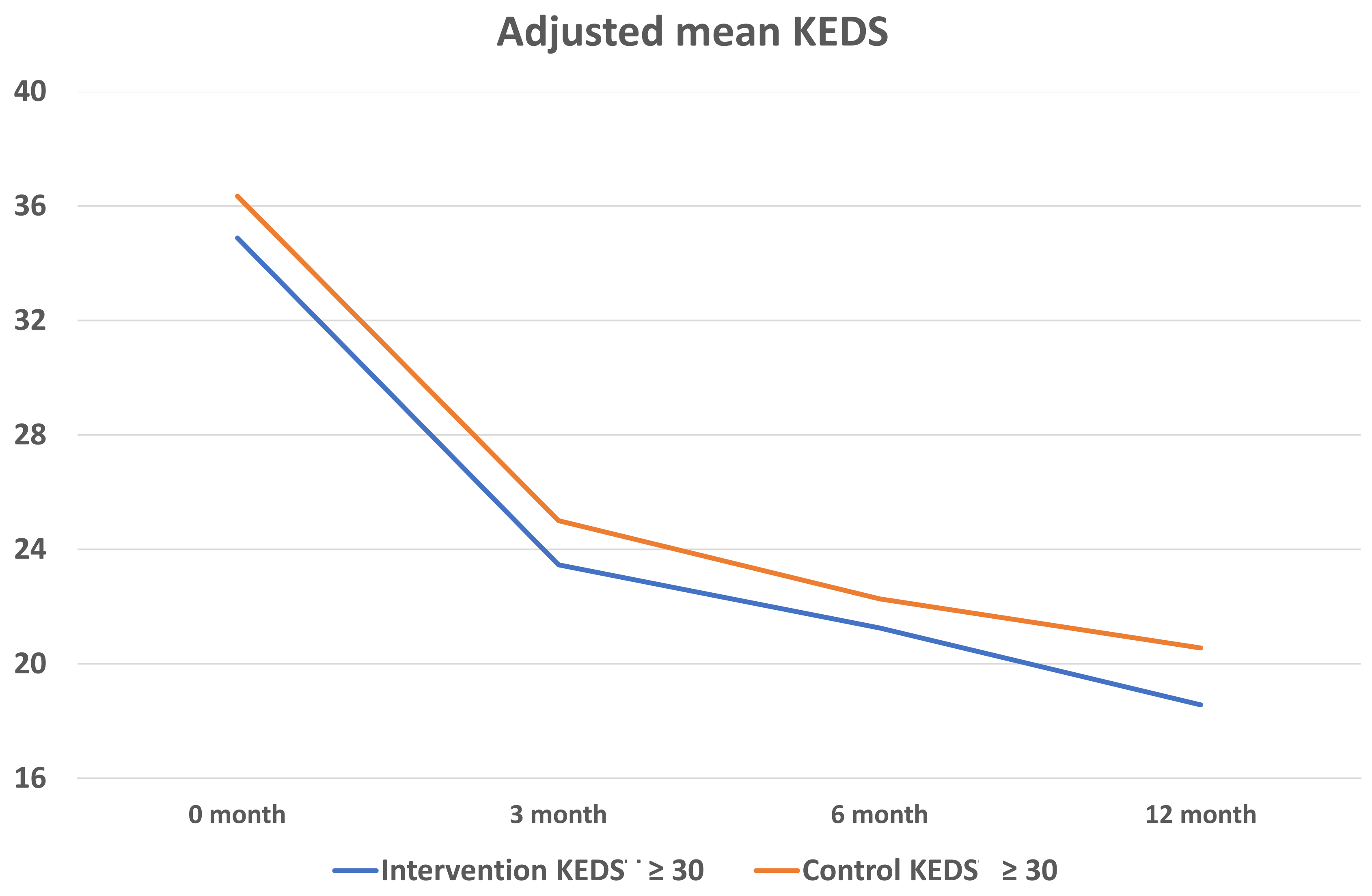
Adjusted mean KEDS
40
36
32
28
24
20
16
0 month
3 month
6 month
12 month
Intervention KEDS ≥ 30
Control KEDS ≥ 30

Supplemental Table S1. Pragmatic characteristics of trial elements in the Co-Work-Care trial

(from https://rethinkingclinicaltrials.org/chapters/pragmatic-clinical-trial/pragmatic-elements-an-introduction-to-precis-2/)

**Trial element**	**Pragmatic characteristics in Co-Work-Care trial**
Research question	Whether enhanced and early cooperation plus early person centred dialogue is more effective in reducing total duration of sick-leave for primary care patients with reduced function because of CMD
Setting	Embedded in the routine primary care setting
Participants	Primary care patients with reduced function because of CMD
Intervention and comparator	Compares two **real-world treatments** using flexible protocols – care manager contact plus enhanced and early cooperation and early person centred dialogue vs care manager contact as usual
Outcomes	Endpoints **clinically relevant** to participants, funders, communities, and healthcare providers
Clinical importance	Purposely designed for making healthcare decisions in settings in which the intervention will be implemented and be used. Important clinical questions can be answered directly from trial results

For peer review only

CONSORT 2010 checklist of information to include when reporting a randomised trial*

Section/Topic	Item No	Checklist item	Reported on page No
**Title and abstract**			
	1a	Identification as a randomised trial in the title	1
	1b	Structured summary of trial design, methods, results, and conclusions (for specific guidance see CONSORT for abstracts)	2,3
**Introduction**			
Background and objectives	2a	Scientific background and explanation of rationale	4-6
	2b	Specific objectives or hypotheses	6
**Methods**			
Trial design	3a	Description of trial design (such as parallel, factorial) including allocation ratio	7-8
	3b	Important changes to methods after trial commencement (such as eligibility criteria), with reasons	
Participants	4a	Eligibility criteria for participants	8
	4b	Settings and locations where the data were collected	8
Interventions	5	The interventions for each group with sufficient details to allow replication, including how and when they were actually administered	8-10
Outcomes	6a	Completely defined pre-specified primary and secondary outcome measures, including how and when they were assessed	13
	6b	Any changes to trial outcomes after the trial commenced, with reasons	
Sample size	7a	How sample size was determined	15
	7b	When applicable, explanation of any interim analyses and stopping guidelines	
Randomisation:			
Sequence generation	8a	Method used to generate the random allocation sequence	8
	8b	Type of randomisation; details of any restriction (such as blocking and block size)	8-9
Allocation concealment mechanism	9	Mechanism used to implement the random allocation sequence (such as sequentially numbered containers), describing any steps taken to conceal the sequence until interventions were assigned	8-9
Implementation	10	Who generated the random allocation sequence, who enrolled participants, and who assigned participants to interventions	8, 11,12
Blinding	11a	If done, who was blinded after assignment to interventions (for example, participants, care providers, those	9

For peer review only

For peer review only - http://bmjopen.bmj.com/site/about/guidelines.xhtml

		assessing outcomes) and how	
	11b	If relevant, description of the similarity of interventions	8, 11,12, Table 1
Statistical methods	12a	Statistical methods used to compare groups for primary and secondary outcomes	14-15
	12b	Methods for additional analyses, such as subgroup analyses and adjusted analyses	14-15
**Results**			
Participant flow (a diagram is strongly recommended)	13a	For each group, the numbers of participants who were randomly assigned, received intended treatment, and were analysed for the primary outcome	16, Fig 1,
	13b	For each group, losses and exclusions after randomisation, together with reasons	Fig 1
Recruitment	14a	Dates defining the periods of recruitment and follow-up	16
	14b	Why the trial ended or was stopped	16
Baseline data	15	A table showing baseline demographic and clinical characteristics for each group	Table 2
Numbers analysed	16	For each group, number of participants (denominator) included in each analysis and whether the analysis was by original assigned groups	Table 2, Table 4
Outcomes and estimation	17a	For each primary and secondary outcome, results for each group, and the estimated effect size and its precision (such as 95% confidence interval)	Fig 2, Table 3 Table 5
	17b	For binary outcomes, presentation of both absolute and relative effect sizes is recommended	
Ancillary analyses	18	Results of any other analyses performed, including subgroup analyses and adjusted analyses, distinguishing pre-specified from exploratory	Supplement. Fig S1
Harms	19	All important harms or unintended effects in each group (for specific guidance see CONSORT for harms)	22
**Discussion**			
Limitations	20	Trial limitations, addressing sources of potential bias, imprecision, and, if relevant, multiplicity of analyses	25-26
Generalisability	21	Generalisability (external validity, applicability) of the trial findings	25
Interpretation	22	Interpretation consistent with results, balancing benefits and harms, and considering other relevant evidence	26,27
**Other information**			
Registration	23	Registration number and name of trial registry	3
Protocol	24	Where the full trial protocol can be accessed, if available	28
Funding	25	Sources of funding and other support (such as supply of drugs), role of funders	30

*We strongly recommend reading this statement in conjunction with the CONSORT 2010 Explanation and Elaboration for important clarifications on all the items. If relevant, we also recommend reading CONSORT extensions for cluster randomised trials, non-inferiority and equivalence trials, non-pharmacological treatments, herbal interventions, and pragmatic trials. Additional extensions are forthcoming: for those and for up to date references relevant to this checklist, see www.consort-statement.org.

For peer review only - http://bmjopen.bmj.com/site/about/guidelines.xhtml

For peer review only

For peer review only - http://bmjopen.bmj.com/site/about/guidelines.xhtml

Table 1

Checklist of items to include when reporting a cluster randomised trial (adaptations from standard guidelines in italic)

Paper section and topic	Item	Descriptor	page
**Title and abstract**			2
Design	1*	How participants were allocated to interventions (eg random allocation, randomised, or randomly assigned), specifying that allocation was based on clusters	2
**Introduction**			
Background	2*	Scientific background and explanation of rationale, including the rationale for using a cluster design	6
**Methods**			
Participants	3*	Eligibility criteria for participants and clusters and the settings and locations where the data were collected	8
Interventions	4*	Precise details of the interventions intended for each group, whether they pertain to the individual level, the cluster level, or both, and how and when they were actually administered	8-10
Objectives	5*	Specific objectives and hypotheses and whether they pertain to the individual level, the cluster level, or both	6
Outcomes	6*	Report clearly defined primary and secondary outcome measures, whether they pertain to the individual level, the cluster level, or both, and, when applicable, any methods used to enhance the quality of measurements (eg multiple observations, training of assessors)	13
Sample size	7*	How total sample size was determined (including method of calculation, number of clusters, cluster size, a coefficient of intracluster correlation (ICC or k), and an indication of its uncertainty) and, when applicable, explanation of any interim analyses and stopping rules	15
Randomisation:			
Sequence generation	8*	Method used to generate the random allocation sequence, including details of any restriction (eg blocking, stratification, matching)	8
Allocation concealment	9*	Method used to implement the random allocation sequence, specifying that allocation was based on clusters rather than individuals and clarifying whether the sequence was concealed until interventions were assigned	8-9
Implementation	10	Who generated the allocation sequence, who enrolled participants, and who assigned participants to their groups	11,12
Blinding (masking)	11	Whether participants, those administering the interventions, and those assessing the outcomes were blinded to group assignment. If done, how the success of blinding was evaluated	9
Statistical methods	12*	Statistical methods used to compare groups for primary outcome(s) indicating how clustering was taken into account, methods for additional analyses, such as subgroup analyses and adjusted analyses	14-15
**Results**			
Participant flow	13*	Flow of clusters and individual participants through each stage (a diagram is strongly recommended). Specifically, for each group report the numbers of clusters and participants randomly assigned, receiving intended treatment, completing the study protocol, and analysed for the primary outcome. Describe protocol deviations from study as planned, together with reasons	Fig 1
Recruitment	14	Dates defining the periods of recruitment and follow up	16

For peer review only - http://bmjopen.bmj.com/site/about/guidelines.xhtml

Paper section and topic	Item	Descriptor	page
Baseline data	15*	Baseline information for each group for the individual and cluster levels as applicable	Table 2
Numbers analysed	16*	Number of clusters and participants (denominator) in each group included in each analysis and whether the analysis was by intention to treat. State the results in absolute numbers when feasible (eg 10/20 not 50%)	Fig 1 Table 2 and 4
Outcomes and estimation	17*	For each primary and secondary outcome, a summary of results for each group for the individual or cluster level as applicable, and the estimated effect size and its precision (eg 95% confidence interval) and a coefficient of intracluster correlation (ICC or k) for each primary outcome.	Fig 2, Table 3 and 5
Ancillary analyses	18	Address multiplicity by reporting any other analyses performed, including subgroup analyses and adjusted analyses, indicating those prespecified and those exploratory	Suppl.fig S-1
Adverse events	19	All important adverse events or side effects in each intervention group	22
**Discussion**			
Interpretation	20	Interpretation of the results, taking into account study hypotheses, sources of potential bias or imprecision and the dangers associated with multiplicity of analyses and outcomes.	25, 26
Generalisability	21*	Generalisability (external validity) to individuals and/or clusters (as relevant) of the trial findings	25
Overall evidence	22	General interpretation of the results in the context of current evidence	27

- ↵* Addition to CONSORT guidelines 20012

For peer review only

For peer review only - http://bmjopen.bmj.com/site/about/guidelines.xhtml
